# Computational Design of Rhenium(I) Carbonyl Complexes for Anticancer Photodynamic Therapy

**DOI:** 10.1021/acs.inorgchem.1c03130

**Published:** 2021-12-16

**Authors:** Daniel Álvarez, M. Isabel Menéndez, Ramón López

**Affiliations:** Departamento de Química Física y Analítica, Facultad de Química, Universidad de Oviedo, C/ Julián Clavería 8, 33006 Oviedo, Spain

## Abstract

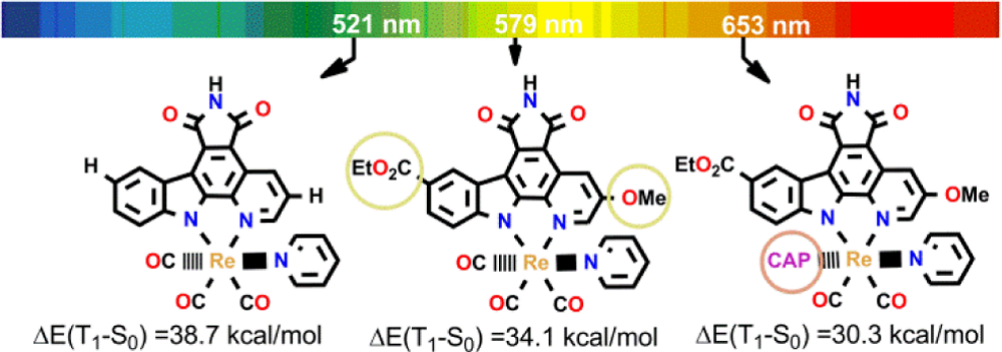

New Re(I) carbonyl complexes are proposed as candidates for photodynamic therapy after investigating the effects of the pyridocarbazole-type ligand conjugation, addition of substituents to this ligand, and replacement of one CO by phosphines in [Re(pyridocarbazole)(CO)_3_(pyridine)] complexes by means of the density functional theory (DFT) and time-dependent DFT. We have found, first, that increasing the conjugation in the bidentate ligand reduces the highest occupied molecular orbital (HOMO)–lowest unoccupied molecular orbital (LUMO) energy gap of the complex, so its absorption wavelength red-shifts. When the enlargement of this ligand is carried out by merging the electron-withdrawing 1*H*-pyrrole-2,5-dione heterocycle, it enhances even more the stabilization of the LUMO due to its electron-acceptor character. Second, the analysis of the shape and composition of the orbitals involved in the band of interest indicates which substituents of the bidentate ligand and which positions are optimal for reducing the HOMO–LUMO energy gap. The introduction of electron-withdrawing substituents into the pyridine ring of the pyridocarbazole ligand mainly stabilizes the LUMO, whereas the HOMO energy increases primarily when electron-donating substituents are introduced into its indole moiety. Each type of substituents results in a bathochromic shift of the lowest-lying absorption band, which is even larger if they are combined in the same complex. Finally, the removal of the π-backbonding interaction between Re and the CO trans to the monodentate pyridine when it is replaced by phosphines PMe_3_, 1,4-diacetyl-1,3,7-triaza-5-phosphabicyclo[3.3.1]nonane (DAPTA), and 1,4,7-triaza-9-phosphatricyclo[5.3.2.1]tridecane (CAP) causes another extra bathochromic shift due to the destabilization of the HOMO, which is low with DAPTA, moderate with PMe_3_, but especially large with CAP. Through the combination of the PMe_3_ or CAP ligands with adequate electron-withdrawing and/or electron-donating substituents at the pyridocarbazole ligand, we have found several complexes with significant absorption at the therapeutic window. In addition, according to our results on the singlet–triplet energy gap, all of them should be able to produce cytotoxic singlet oxygen.

## Abstract 2

Computations are crucial for proposing new Re(I) carbonyl complexes with very interesting features that make them promising compounds for photodynamic therapy.

## Introduction

Since Raab’s pioneering work on the effects of visible light and acridine dye on paramecia,^1^ photodynamic therapy (PDT) has become a significant complementary or alternative well-established approach for cancer treatment.^2−15^ PDT offers a temporal and spatial control of the treatment with minimal side effects, which starts with the administration of a photosensitizer (PS, a photoactivatable molecule), followed by its excitation by light irradiation at a specific wavelength. The light absorption promotes the PS from its singlet ground state (S_0_) to a singlet excited state (S_1_), which is an unstable and short-lived one. As a consequence, the excited PS can return to its ground state by releasing its extra energy as heat or fluorescence. Alternatively, the PS can evolve from the S_1_ state to a long-lived triplet excited state (T_1_) that can transfer its energy by phosphorescence or collide with other molecules to generate chemically reactive species (CRS), which kill cancer cells. The mechanisms for CRS formation in PDT are generally classified into two types. In the so-called type I mechanism, the PS in its T_1_ state typically interacts with biological organic substrates through an electron transfer to form radicals, which then react with ground-state oxygen (^3^O_2_) to generate reactive oxygen species. The mechanism known as type II mostly implies the direct reaction of the PS in its T_1_ state with ^3^O_2_ by energy transfer to yield singlet oxygen (^1^O_2_), which causes cellular damage to death by necrosis or apoptosis. Although both mechanisms are believed to occur simultaneously,^4,16^ it is widely accepted that the type II mechanism is the predominant one in anticancer PDT treatments.^12,17^ As such, the three key elements for the therapeutic efficacy of PDT are the nature of the PS, a light of appropriate wavelength, and the presence of molecular oxygen. To achieve the optimal penetration of light into human body tissues and to generate the PS in its triplet excited state capable of producing cytotoxic singlet oxygen, the wavelength of the light must fall in the therapeutic window (around 620–850 nm).^12,18^ The lower limit of this window is fixed to avoid light absorption by endogenous biomolecules, hence maximizing the depth of penetration into the tissue, whereas the upper limit comes from the fact that the energy gap between the ground and triplet states of the PS must be larger than the energy required for the transformation ^3^O_2_ (^3^Σ_g_^–^) → ^1^O_2_ (^1^Δ_g_) (∼22.5 kcal/mol).^19,20^ The amphiphilic character of the PS is also of interest for its efficacy in PDT: its hydrophilicity facilitates its distribution and its lipophilicity its cellular uptake.^12^

Although PDT has been used against a broad variety of cancers (i.e., bile duct, bladder, brain, esophagus, head, lung, neck, pancreas, prostate, skin, etc.), the number of PSs approved for these treatments is very limited.^12,17,21^ Most of these drugs are based on porphyrinoid structures, including porphyrins, chlorins, bacteriochlorins, phthalocyanines, and related structures.^12,17,21^ Even though these PSs are able to absorb light in the therapeutic window and generate cytotoxic singlet oxygen, they have shown drawbacks such as poor solubility in water, aggregation tendency in physiological liquids, and the requirement of an oxygen-rich environment for the production of singlet oxygen.^22−25^ As a result, there is great interest in modifying the existing PSs or developing new classes of PSs. Among the latter, rhenium(I) tricarbonyl complexes have been explored in recent years as potential candidates for PDT^26−35^ due to their rich photophysical and biochemical properties, including polarized emission, high photostability, large Stokes shifts, triplet states with long lifetimes, and biocompatibility,^36−40^ which can also be tuned by varying the ligands.

**Scheme 1:**
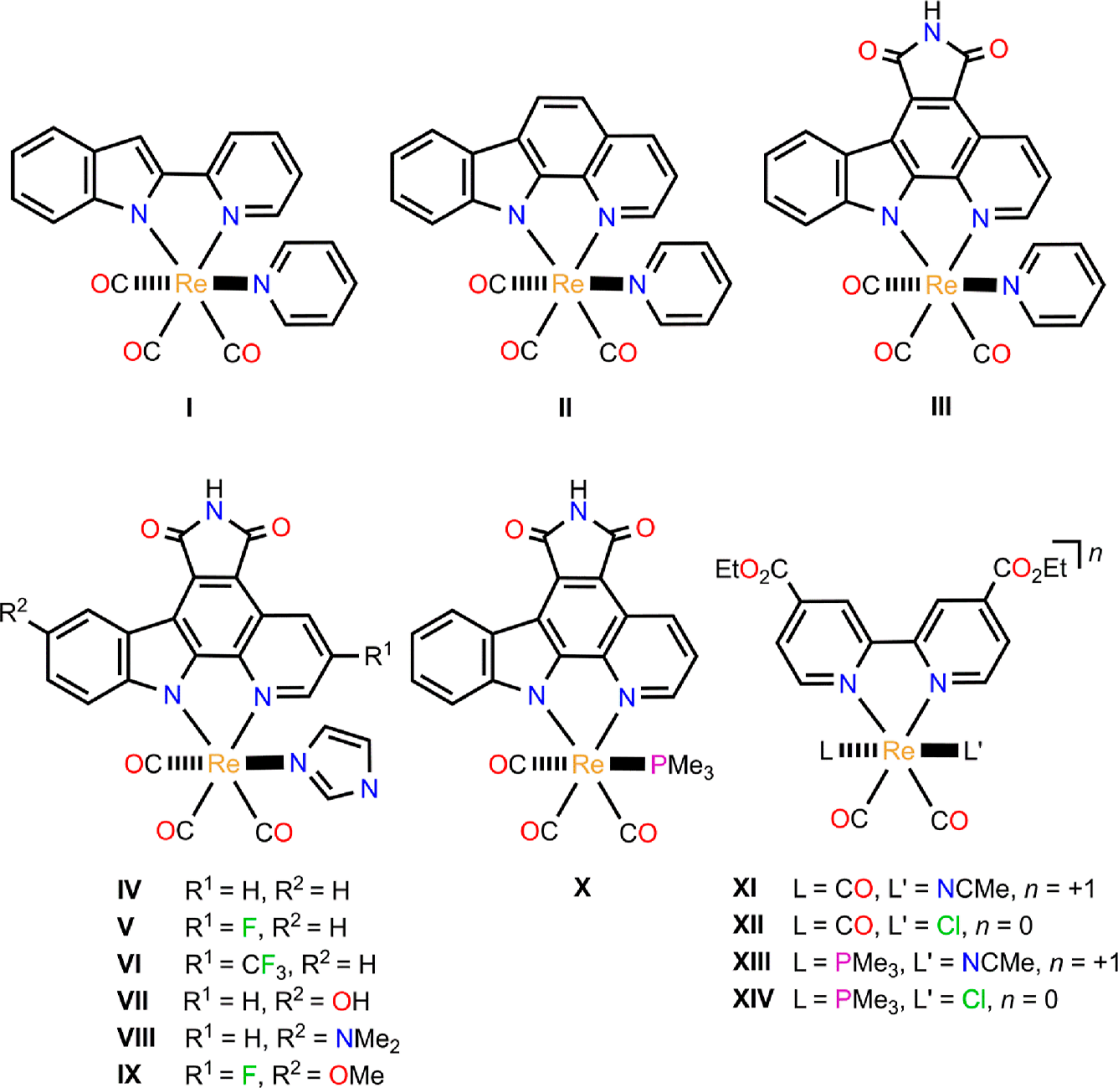
Structure of Rhenium(I) Carbonyl Complexes **I–XIV**

**Scheme 2:**
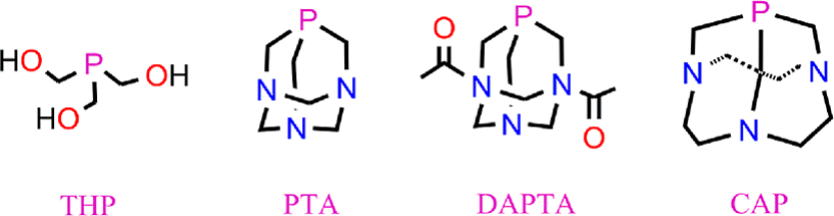
Structure of Phosphines THP, PTA, DAPTA, and CAP

Of particular interest are the structural modifications introduced in the first Re(I) complexes with visible-light-induced anticancer activity reported in 2013.^26,27^ In the starting complexes, rhenium is coordinated to three carbonyl ligands in facial disposition, one pyridine ligand, and one pyridocarbazole-type bidentate ligand (complexes **I–III** in Scheme 1). The expansion of the bidentate ligand of these complexes shifts their light-induced cytotoxicity in cancer cells to longer wavelengths, from λ ≥ 330 nm (complex **I**) to λ ≥ 505 nm (complex **III**), which is desired for PDT to allow a deeper tumor penetration.^26^ This correlates well with the increase in the maximum wavelength (λ_max_) of the lowest-lying absorption band from 373 nm for complex **I** to 512 nm for **III**, as measured in dimethylsulfoxide (DMSO). It was also found that the production of ^1^O_2_ by these complexes is crucial to induce photocytotoxicity. Aiming at obtaining new Re(I) pyridocarbazole complexes for PDT with improved chemical stability and red-shifted visible-light-induced anticancer activity, derivatives of complex **III** were also investigated (complexes **IV–IX** in Scheme 1).^27^ The replacement of the weak π-accepting pyridine ligand in **III** with the strong σ-donating imidazole one (complex **IV** in Scheme 1) does not practically change the value of λ_max_ (Δλ_max_ = 1 nm) in DMSO. On the other hand, the presence of electron-withdrawing substituents in the 3-position of the pyridine moiety (R^1^) of the modified pyridocarbazole ligand in **IV**, complexes **V** (R^1^ = F) and **VI** (R^1^ = CF_3_) in Scheme 1, red-shifts λ_max_ in DMSO. A similar trend was also obtained when electron-donating substituents are introduced into the 5-position of the indole moiety (R^2^) of the modified pyridocarbazole ligand in **IV**, complexes **VII** (R^2^ = OH) and **VIII** (R^2^ = NMe_2_) in Scheme 1. Nonetheless, the latter complexes do not exhibit photocytotoxicity, whereas those containing electron-withdrawing groups at the pyridine moiety do. When both types of substituents are simultaneously present at the pyridocarbazole ligand (R^1^ = F and R^2^ = OMe, complex **IX** in Scheme 1), λ_max_ increases from 513 nm (complex **IV**) to 542 nm (complex **IX**) in DMSO, retaining the photoinduced cytotoxic effect at λ ≥ 620 nm. On the other hand, the substitution of the pyridine ligand in **III** with the strong σ-donating PMe_3_, complex **X** in Scheme 1, hardly affects λ_max_ (Δλ_max_ = 3 nm), as in the replacement of pyridine by imidazole. In contrast, a greater effect on λ_max_ was found when PMe_3_ replaces the CO trans to the monodentate ligand in Re(I) carbonyl complexes containing a substituted bipyridine ligand instead of a pyridocarbazole one. Specifically, red shifts of 100 and 123 nm were reported when going from complexes [Re(deeb)(CO)_3_(NCMe)]^+^ and [Re(deeb)(CO)_3_Cl] (deeb = 4,4′-diethylester-2,2′-bipyridine) to [Re(deeb)(CO)_2_(PMe_3_)(NCMe)]^+^ and [Re(deeb)(CO)_2_(PMe_3_)Cl], respectively (complexes **XI–XIV** in Scheme 1).^41^ As the photochemical properties of Re(I) tricarbonyl complexes are mainly controlled by the metal-to-ligand charge transfer (MLCT) transition, that is, the energy difference between the filled Re d orbitals and the empty π* orbitals of the bidentate ligand, it was reasoned that the replacement of the CO ligand by the strong σ-donating and weak π-accepting PMe_3_ ligand destabilizes the occupied Re d orbitals [highest occupied molecular orbital (HOMO) of the complex], thus explaining the increase in λ_max_ observed.^41^ In this regard, the use of water-soluble phosphine ligands, such as tris-(hydroxymethyl)phosphine (THP), 1,3,5-triaza-7-phosphaadamantane (PTA), 1,4-diacetyl-1,3,7-triaza-5-phosphabicyclo[3.3.1]nonane (DAPTA), and 1,4,7-triaza-9-phosphatricyclo[5.3.2.1]tridecane (CAP)^33,42^ (see Scheme 2), could be useful in obtaining new improved PDT agents based on Re carbonyl complexes. Thus, for instance, Re(I) tricarbonyl complexes bearing bipyridine- or phenanthroline-type bidentate ligands and phosphine ligands THP, PTA, and DAPTA have been reported to exhibit water solubility and certain photocytotoxicity against cancer cells, mainly those containing DAPTA.^33^ In addition, CAP is one of the strongest known electron-donating phosphines with a low steric effect that is capable of promoting the oxidative dissolution of elemental gold to gold(I) and displacing the strongly bonded cyanide ligand of the gold(I) complex [Au(CN)_2_]^−^ to give rise to [Au(CAP)_3_]^+^.^42^ In this scenario, we wondered if the substitution of the CO trans to the pyridine ligand in complex **III** with PMe_3_, DAPTA, and CAP could improve the photocytotoxicity of Re(I) carbonyl pyridocarbazole complexes.

Taking the aforementioned in mind along with the fact that computational chemistry is a reliable tool to obtain and rationalize the photophysical and photochemical properties of transition-metal compounds, we undertook a theoretical investigation on the electronic absorption spectra of a series of rhenium(I)-pyridocarbazole scaffolds taking as the reference complex **III** in Scheme 1. Aiming at providing valuable information for the design of novel improved Re-based PSs for PDT, several aspects will be analyzed: the degree of conjugation, the effect of substituents at the pyridocarbazole ligand, and the replacement of a carbonyl ligand by phosphine ones.

## Results and Discussion

**Scheme 3:**
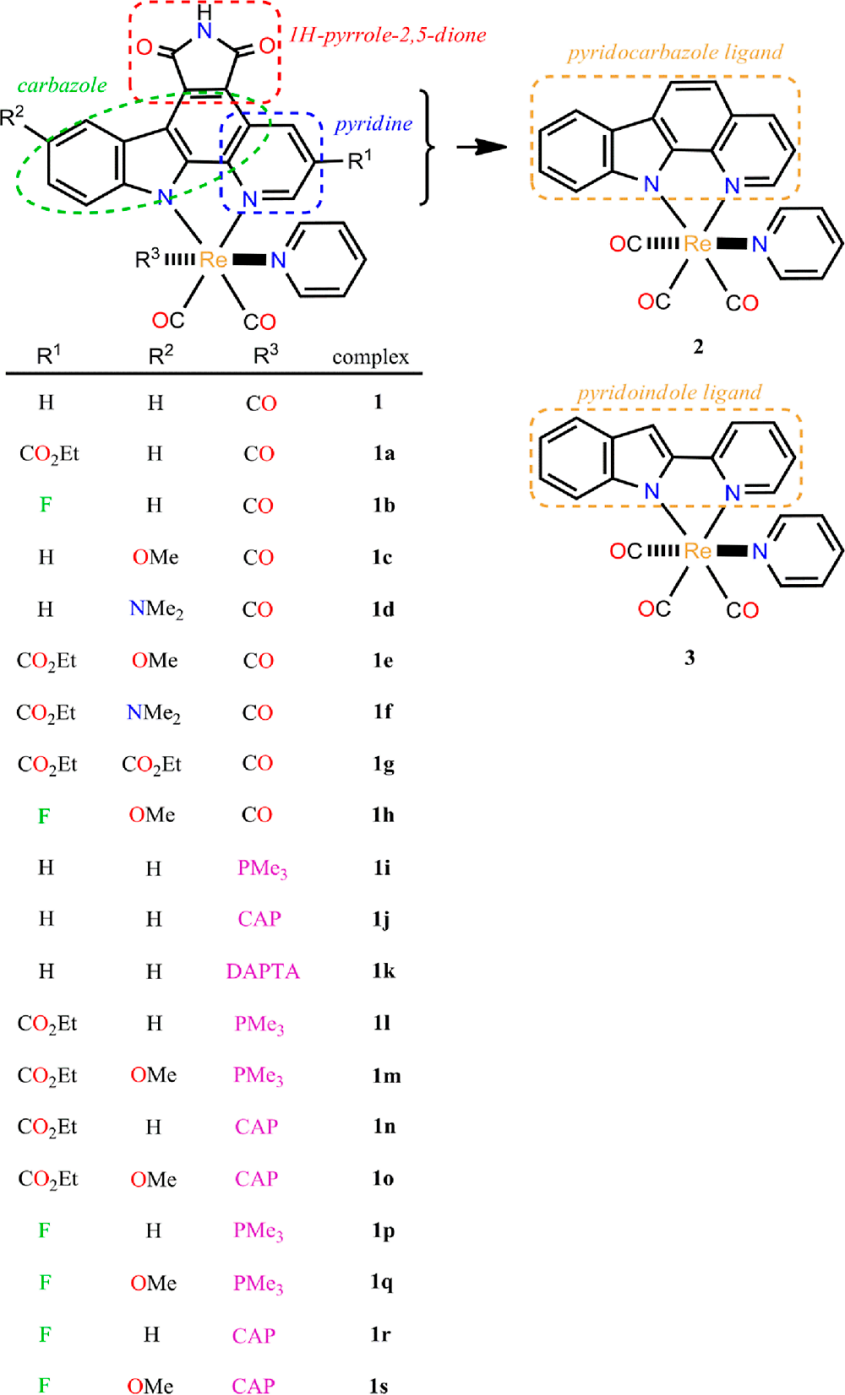
Structure and Notation of the Re(I) Carbonyl Complexes Investigated in This Work

Scheme 3 collects the schematic representation of the whole set of Re(I) carbonyl complexes investigated in the present work along with the acronyms used for them in this section. The geometry of all these species was optimized both in their singlet ground states and in their triplet excited states at the B3LYP-D3/6-31+G(d)-LANL2DZ level of theory, whereas the electronic absorption spectra were obtained at the PCM-TD-M06/6-31+G(d)-LANL2DZ//B3LYP-D3/6-31+G(d)-LANL2DZ level of theory in the DMSO solution (see the Computational Details section for more details). The above computational protocols have been chosen after a thorough discussion based on the validation calculations of the geometry and absorption spectra collected in the Supporting Information (see Discussions 1 and 2 together with Figures S1–S4 and Tables S1–S7). Unless otherwise stated, the complex with the {Re(CO)_3_(pyridine)}^+^ fragment bound to the enlarged pyridocarbazole ligand pyrido[2,3-*a*]pyrrolo[3,4-*c*]carbazole-5,7(6*H*)-dione (**1** in Scheme 3) will be taken as a reference to study the effect of the variation of the size of the pyridocarbazole-type ligand and the addition of substituents to its rings as well as that of the replacement of the carbonyl ligand trans to the pyridine monodentate ligand by phosphine ones on the spectroscopic and photocytotoxic properties of this type of Re(I) complexes. As seen in Scheme 3, all the complexes investigated have two pyridine rings: one is either fused to the carbazole (complexes **1**, **2**, and **1a–1s**) or attached to the indole (complex **3**) of the bidentate ligand and the other is acting as a monodentate ligand in cis disposition to the former one. To avoid some confusion between these two rings, in the following, the pyridine in the bidentate ligand will be referred to as fused or indole-bound pyridine, while the other will be cited as plain pyridine.

### Influence of the Size of the Bidentate Ligand

**Figure 1:**
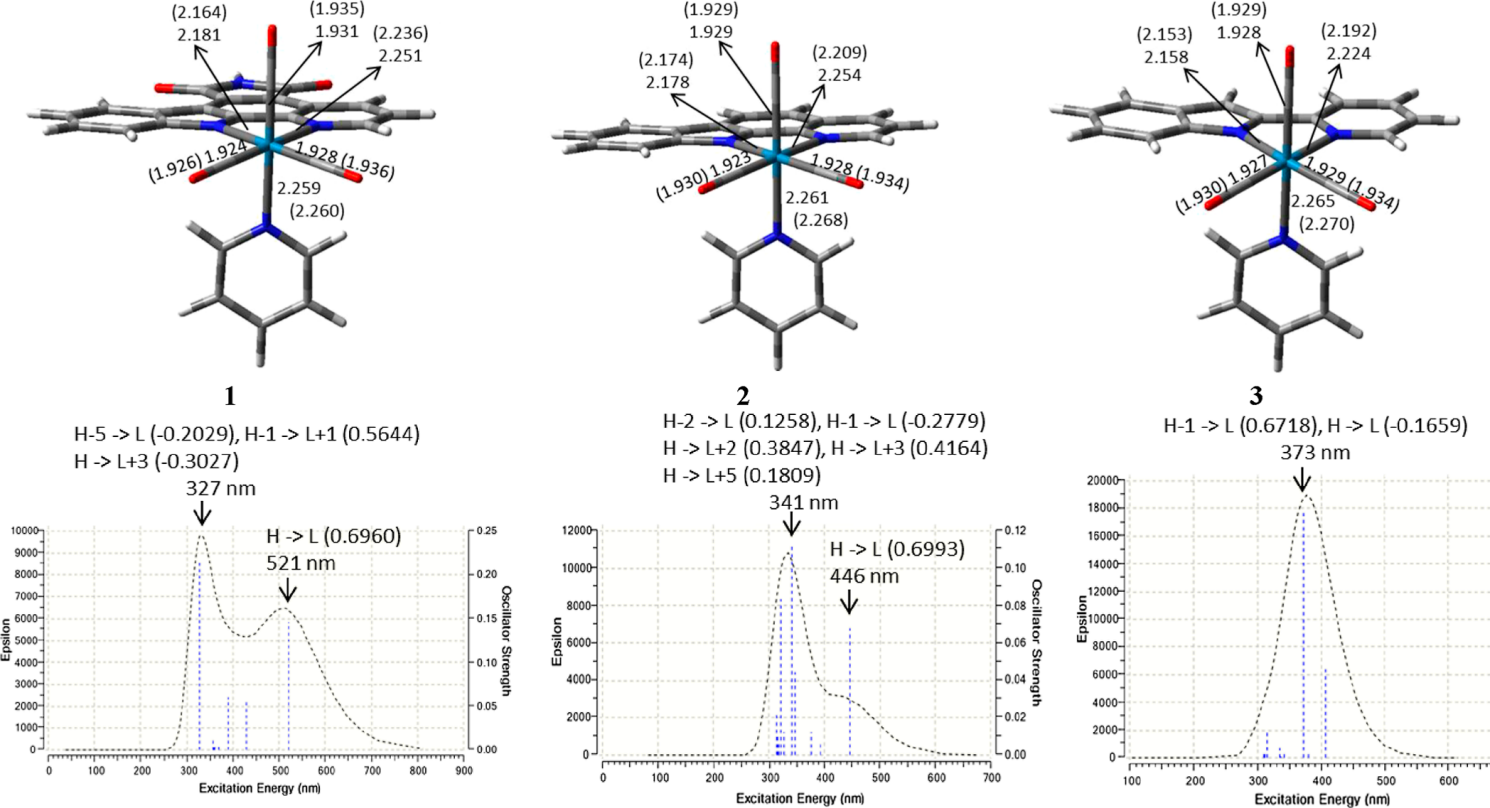
Singlet optimized structures and computed electronic absorption spectra in DMSO obtained for Re(I) tricarbonyl pyridyl complexes **1–3**. Some significant bond distances in Å are collected. Data obtained for triplet optimized structures are shown in parenthesis. The dominant orbital excitations (coefficients in parenthesis) involved in the main absorption transitions are also displayed.

Complexes **1–3** in Scheme 3 have been considered to examine the effect of the degree of expansion of the bidentate ligand on the absorption properties and the photogeneration of cytotoxic singlet oxygen of these Re(I) complexes. The {Re(CO)_3_}^+^ fragment in complex **1** is attached both to a pyridine (py) ligand in trans disposition to a carbonyl ligand and to an enlarged pyridocarbazole (epycb) ligand, which contains heterocycles carbazole, pyridine, and also the 1*H*-pyrrole-2,5-dione ring. The bidentate ligand at complexes **2** and **3** are unsubstituted pyridocarbazole and pyridoindole, respectively. Figure 1 shows the optimized structures and the absorption spectra obtained for complexes **1–3** in their singlet ground states. Concerning the geometry, complexes **1** and **2** present similar coordination distances around the Re center, whereas a moderate shortening of the bond distances between Re and the two N atoms of the bidentate ligand (about 0.025 Å) is observed when going from **1** to **3** (see Table S8 for more details). As seen in Figure 1, the electronic absorption spectrum computed for complex **1** presents two clear absorption bands at λ_max_ values of 327 and 521 nm, in good agreement with the two most intense bands experimentally detected for this compound.^26^ Actually, the λ_max_ value of the most red-shifted band only differs by 9 nm from the experimental one. A third absorption band of lower intensity and located between these two has also been reported experimentally.^26^ Although the theoretical absorption spectrum obtained for **1** does not properly reflect that intermediate band (Figure 1), its third most intense excitation appears at 390 nm (Table S9), which falls in the region of the least intense absorption band detected experimentally. In accordance with the experimental findings, the absorption spectrum obtained for complex **2** shows two absorption bands at λ_max_ values of 341 and 446 nm, as displayed in Figure 1. The latter value fully coincides with the most red-shifted λ_max_ reported experimentally.^26^ In the case of complex **3**, the electronic absorption spectrum displayed in Figure 1 shows only one absorption band at 373 nm, which is also equal to the experimental value.^26^

**Figure 2:**
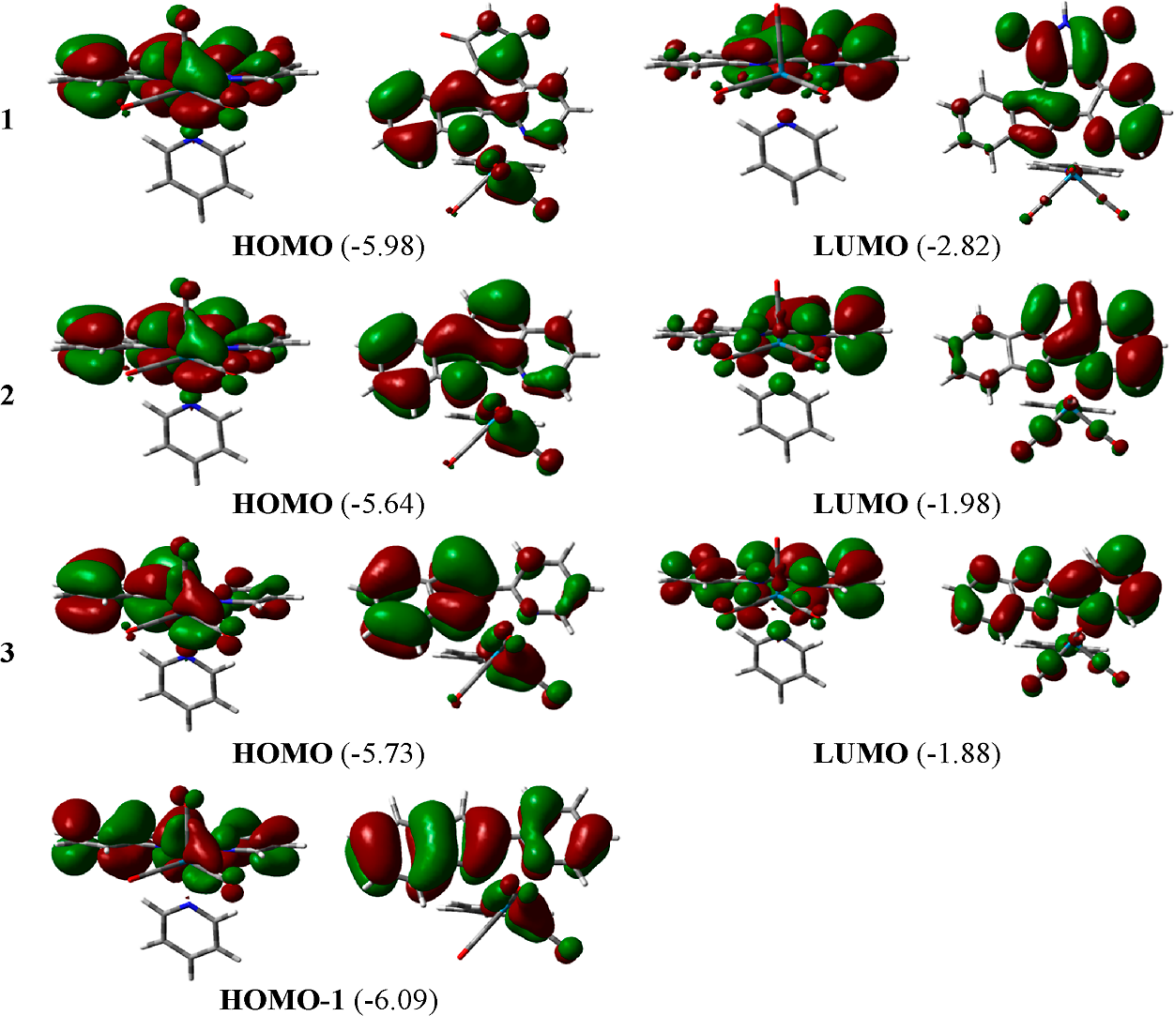
Contour maps of the frontier KSOs involved in the dominant orbital excitations of the lowest-lying absorption band found for Re(I) tricarbonyl pyridyl complexes **1–3**. Orbital energies in electronvolts are shown in parentheses. Two views are given for each orbital.

Looking at the most red-shifted absorption band obtained for complexes **1–3**, which is the most interesting one for a potential PS for PDT, we see that the decrease in the size of the bidentate ligand when going from complex **1** to **3** results in a hypsochromic shift from 521 to 373 nm, as experimentally found by Meggers and co-workers.^26^ The Kohn–Sham orbitals (KSOs) involved in that band give us information about such a shift. For both **1** and **2** complexes, the most red-shifted band is mainly described as a HOMO → LUMO transition, whereas the main contribution is a HOMO – 1 → LUMO transition in the case of complex **3**, with a minor participation of the HOMO → LUMO one. The shape and the energy of these KSOs are displayed in Figure 2. In complexes **1–3**, the HOMOs are quite similar in composition but with destabilizations of 0.34 and 0.25 eV for **2** and **3** relative to **1**, respectively. Thus, it is mainly composed of a Re d orbital combined with π* orbitals of two CO ligands, one in trans and one in cis to the py ligand and a π orbital located in the bidentate one, primarily in the indole moiety. The HOMO – 1 in complex **3** lies 0.11 eV below the HOMO of complex **1** and is built with the participation of a Re d orbital combined with two CO π* orbitals and a π orbital extended over the whole pyridoindole ligand. It is expected that an increase in the conjugation will cause a destabilization of the HOMO, as observed when going from **3** to **2**. In the case of complex **1**, there are extra features associated to the presence of the 1*H*-pyrrole-2,5-dione heterocycle since it acts as an electron-withdrawing group decreasing the electron density of the epycb ligand, thus stabilizing its HOMO. This effect prevails over the destabilization produced by the ligand expansion and causes the observed stability of the HOMO of complex **1**.

On the other hand, the lowest unoccupied molecular orbital (LUMO) is formed by a π* bidentate ligand orbital in the three complexes, with a larger contribution of the fused or indole-bound pyridine ring than that of the indole one. In the case of complex **1**, it is noteworthy that the 1*H*-pyrrole-2,5-dione heterocycle also contributes to its LUMO. As a consequence, the energy of the LUMO is greatly affected by the number of rings at this bidentate ligand. Hence, the LUMO at complex **1** is the most stable one, with an energy of −2.82 eV, and this value increases to −1.98 and −1.88 eV when moving to complexes **2** and **3**, respectively. With respect to complex **1**, the presence of the electron-withdrawing 1*H*-pyrrole-2,5-dione heterocycle contributes to provide large stability to its LUMO.

Comparing complexes **2** and **3**, it is clear that an increase in the conjugation at the bidentate ligand reduces the HOMO–LUMO energy gap by destabilizing the HOMO and stabilizing the LUMO. This fact, along with the larger contribution of the HOMO – 1 than that of the HOMO to the lowest-lying absorption band of complex **3**, explains the observed bathochromic shift at complex **2** relative to that of **3**. For complex **1**, both the HOMO and the LUMO undergo a stabilizing effect, which is much more pronounced in the LUMO, and thus, the gap between the HOMO and the LUMO decreases compared to that of the other compounds, leading, in turn, to the longest λ_max_ among the three complexes. The shape of the HOMO (or HOMO – 1) and LUMO orbitals indicates that the most red-shifted absorption band for complexes **1–3** has a mixed metal-to-ligand charge transfer (^1^MLCT, *d*π_Re(CO)_2__ → π*_bidentate_) and intraligand charge transfer (^1^ILCT, π_bidentate_ → π*_bidentate_) character.

**Figure 3:**
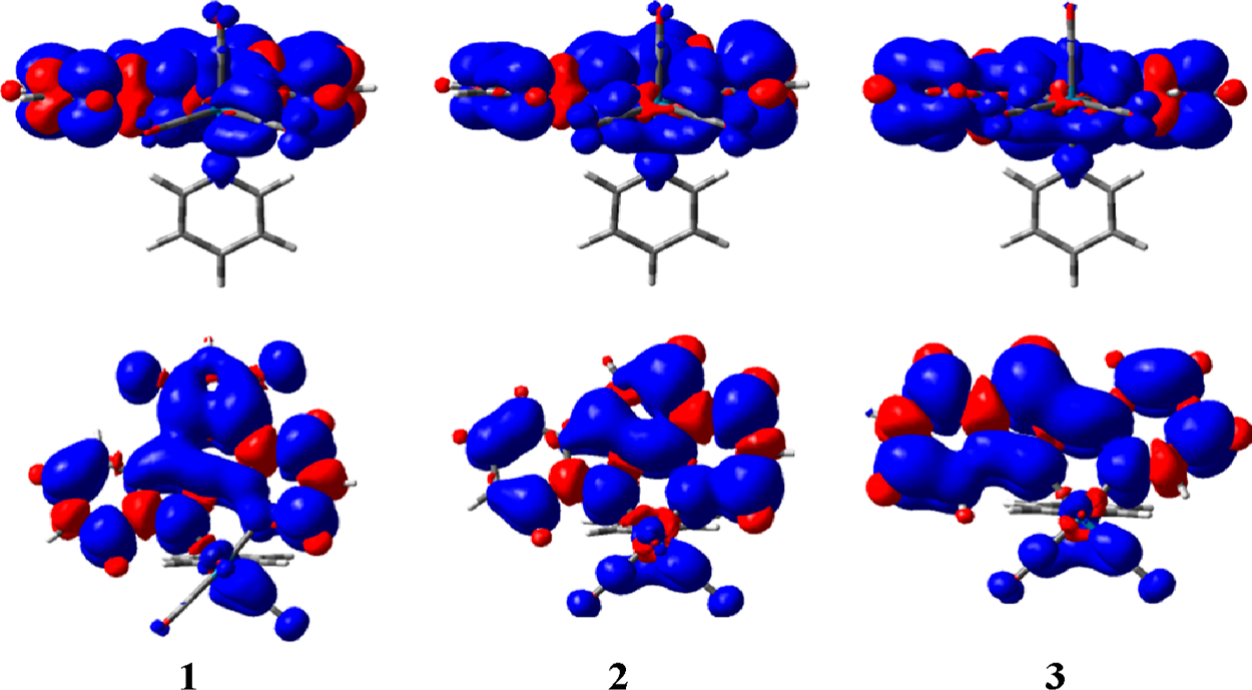
Spin density plots (an isovalue of 0.0004) of the optimized triplet states for complexes **1–3**. For clarity, two views are given for each species.

Although complexes **1–3** do not show absorption bands within the therapeutic window (∼620–850 nm), which limits their use as PSs in PDT, Meggers and co-workers have found that they are capable of producing singlet oxygen when irradiated with light of the appropriate wavelength.^26^ Thus, only complex **1** is able to yield singlet oxygen after irradiation with λ ≥ 505 nm due to the lack of absorption of the other two complexes in this region of the spectrum, whereas all of them did generate such a cytotoxic species upon irradiation with λ ≥ 330 nm. Aiming at examining the ability of complexes **1–3** to produce singlet oxygen, we optimized their structures in their triplet excited states (T_1_), obtained spin density plots to characterize their nature, and computed the energy gap between T_1_ and the singlet ground state (S_0_), Δ*E*_ST_ (Tables S10 and S11, respectively). Overall, the singlet and triplet optimized structures obtained for each complex are very similar (Figure 1 and Table S12). For complex **1**, when passing from S_0_ to T_1_, the bond distances between Re and the N atoms of the indole (N_indole_) and fused pyridine (N_pyridine_) moieties of the bidentate ligand show the largest variations as they shorten by 0.017 and 0.015 Å, respectively (Figure 1). The remaining metal–ligand bond distances only lengthen in the range between 0.001 and 0.008 Å. For complexes **2** and **3**, upon going from S_0_ to T_1_, the most significant variation was detected for the Re–N_pyridine_ bond distance, which notably shortens by 0.045 and 0.032 Å, respectively. The Re–N_indole_ bond length slightly shortens by 0.004 Å (complex **2**) and 0.005 Å (complex **3**), whereas the remaining metal–ligand distances lengthen in a range similar to that found for complex **1** (0.001–0.007/0.005 Å for **2**/**3**). The spin density distributions of the optimized triplet state for complexes **1–3** are localized on the bidentate ligand and the Re atom with CO ligands (see Figure 3). These plots suggest a mixed ^3^MLCT–^3^ILCT character of the excited state in these three complexes, which resembles the one found for the corresponding singlet ground states. The energy difference Δ*E*_ST_ values obtained for complexes **1**, **2**, and **3** are 38.7, 47.1, and 49.1 kcal/mol, respectively. All these values are notably larger than the minimum energy required, 22.5 kcal/mol,^19,20^ to transform ^3^O_2_ into ^1^O_2_, which is in consonance with the singlet oxygen production reported experimentally for complexes **1–3**.^26^

### Effect of the Substituents at the Enlarged Pyridocarbazole Ligand

**Figure 4:**
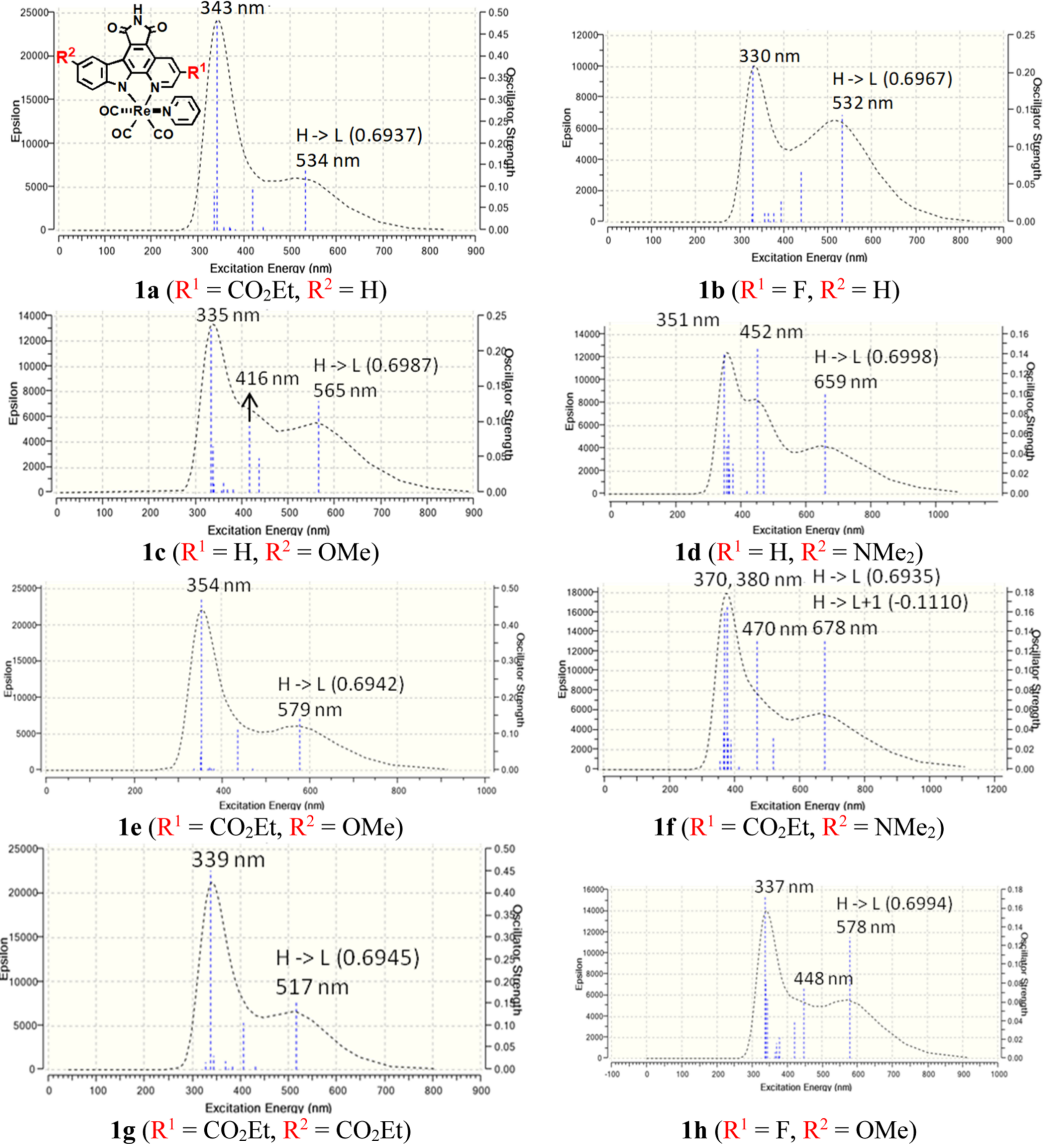
Computed electronic absorption spectra in DMSO for Re(I) tricarbonyl pyridyl complexes bearing substituted epycb ligands **1a–1h**. The dominant orbital excitations (coefficients in parentheses) involved in the most red-shifted absorption transition are also displayed. General structures of complexes **1a–1h** are also given at the top of the left column.

Inspired by the interesting spectroscopic properties shown by Re(I) carbonyl complexes containing imidazole and substituted pyridocarbazoles,^27^ which displayed photocytotoxicity upon irradiation of light with λ ≥ 620 nm, we decided to investigate the influence of adding electron-donating and electron-withdrawing groups to the bidendate ligand of **1** in eight Re(I) substituted epycb complexes (**1a–1h** in Figure 4). Particularly, taking into account the shape of the KSOs involved in the lowest-lying absorption band, we have explored the effect of adding the electron-withdrawing CO_2_Et (**1a**) and F (**1b**) substituents at the C3 atom of the fused pyridine ring of the epycb ligand (R^1^ substitution), as well as that of introducing electron-donating OMe (**1c**) and NMe_2_ (**1d**) groups at the C5 atom of its indole moiety (R^2^ substitution). Finally, we have also considered complexes where both types of substituents are present simultaneously at the diimine ligand (**1e–1h**), with the exception of complex **1g**, which has CO_2_Et groups at both the indole and the fused pyridine moieties of the epycb ligand.

The optimized geometries of complexes **1a–1h** in their singlet ground states are collected in the Supporting Information (Figure S5 and Table S8). The introduction of substituents into the epycb ligand hardly produces any geometrical changes relative to **1** (Table S13). For example, the Re–ligand bond distances in **1a–1h** compared to the analogous ones in **1** only vary in the range between −0.004 and 0.003 Å, whereas variations between −0.8 and 2.0° were found for the most relevant bond and dihedral angles. As experimentally found for complex **1** and analogous Re(I) epycb complexes bearing the imidazole monodentate ligand instead of the py one,^26,27^ the shortest wavelength absorption band obtained for complexes **1a–1h** is the most intense one and ranges from 330 to 380 nm (see Figure 4). The second most intense absorption band has the longest wavelength, with λ_max_ ranging from 517 to 678 nm. When detected, the intermediate absorption band is the least intense one by far, and it varies between 416 and 470 nm. Focusing on the potential use of these complexes as PSs for PDT, we turn our attention to the variation of the λ_max_ values of the lowest-lying absorption band obtained for complexes **1a–1h**, taking as a reference the one obtained for complex **1**.

The electronic absorption spectra of the complexes **1a–1d**, which have only one substituent at the epycb ligand, are shown in the first part of Figure 4 and Table S14. The complexes bearing an electron-withdrawing group present a similar absorption band, with λ_max_ values of 534 and 532 nm for **1a** and **1b**, respectively. Thus, the bathochromic shift with respect to complex **1** (λ_max_ = 521 nm) is slightly more pronounced for the CO_2_Et group than that for the F substituent. On the other hand, the introduction of electron-donating substituents, as in **1c** and **1d**, shifts λ_max_ to 565 and 659 nm, respectively. It is noteworthy that the strong electron-donating effect of NMe_2_ induces a red shift that is 94 nm longer than the one produced by OMe. Besides, these electron-donating substituents at the indole moiety generate a larger red shift than the electron-withdrawing ones at the fused pyridine moiety of the epycb ligand. A similar, although less pronounced, trend in Re-substituted epycb complexes bearing the imidazole ligand was found by Meggers and co-workers.^27^

Then, we investigated the effect of combining the relatively best electron-withdrawing group CO_2_Et at the fused pyridine ring of the epycb ligand with the OMe (**1e**) and NMe_2_ (**1f**) substituents at the indole moiety, as well as with a CO_2_Et group (**1g**), to explore the effect of adding two electron-withdrawing substituents at the bidentate ligand. A complex with F instead of CO_2_Et and OMe (**1h**) has also been considered for comparison purposes. The electronic absorption spectra of those complexes are displayed in the last part of Figure 4 and Table S14.

Comparing complexes **1e** and **1f** with the corresponding ones containing only one of the substituents at the epycb ligand, it is clear that the combination of an electron-withdrawing group at the fused pyridine ring with an electron-donating one at the indole moiety provokes the strongest bathochromic shift of the lowest-lying absorption band. Hence, the band of **1c** (λ_max_ = 565 nm) is shifted to 579 nm when the CO_2_Et group is added (complex **1e**). That lengthening of λ_max_ (14 nm) is very similar to the one produced in complex **1** when the same electron-withdrawing group is the only one added at its fused pyridine ring (13 nm when going from **1** to **1a**). This is also the case of the complex with the other electron-withdrawing substituent, (R^1^ = F at **1h**), where the longest λ_max_ increases by 13 nm when compared to **1c**. Similarly, the λ_max_ of **1d** (659 nm), with the NMe_2_ substituent, is shifted to 678 nm for **1f**, making **1f** the complex with the most red-shifted absorption. Finally, regarding complex **1g**, a hypsochromic shift of the lowest-lying absorption band is observed, with a λ_max_ value of 517 nm. This confirms that the introduction of electron-withdrawing groups at the indole ring of the epycb ligand provokes the opposite effect to that of the electron-donating ones at this position.

**Figure 5:**
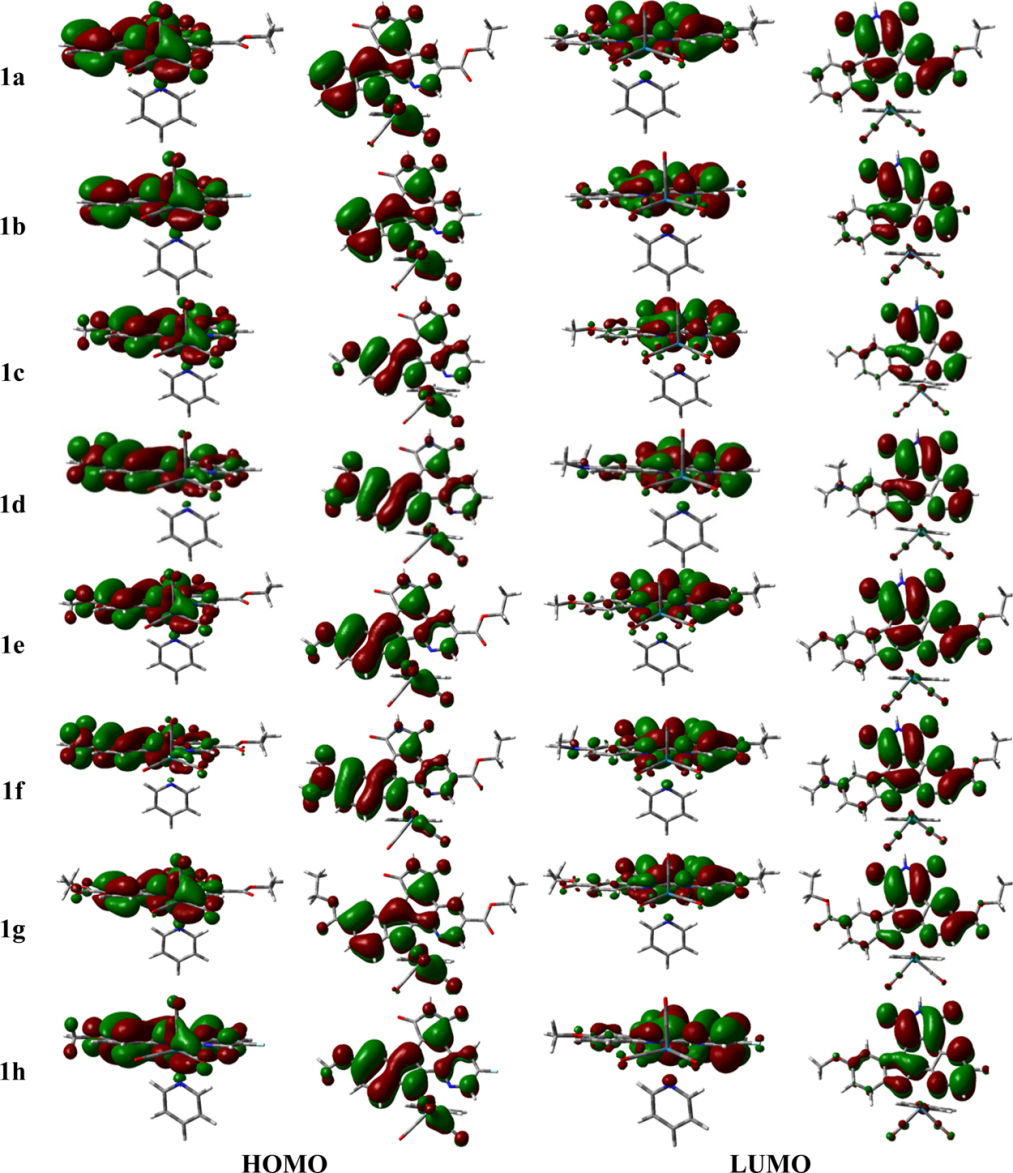
Contour maps of the main frontier KSOs implied in the dominant orbital excitations of the lowest-lying absorption band found for Re(I) complexes with a substituted epycb ligand **1a–1h**. Two views are given for each orbital.

As for complex **1**, the lowest-lying absorption band of Re(I) compounds **1a–1h** is mainly attributed to the HOMO–LUMO transition (Figure 4). The HOMO of all complexes, except those containing the NMe_2_ substituent (**1d** and **1f**), is essentially the same as that previously described for complex **1**, that is, a mixture of a *d*π_Re(CO)_2__ orbital with a π_epycb_ orbital, which is mainly centered on the indole moiety (Figure 5). In contrast, the HOMO of complexes **1d** and **1f** has a negligible contribution of the *d*π_Re(CO)_2__ orbital. The LUMO of all the complexes is primarily a π_epycb_^*^ orbital, expanded across all the epycb ligand but the benzene ring of the indole moiety (see Figure 5). Therefore, on the whole, the HOMO → LUMO transition has a mixed ^1^MLCT and ^1^ILCT (*d*π_Re(CO)_2__ + π_epycb_ → π_epycb_^*^) character for all the complexes except **1d** and **1f**, for which the orbital transition primarily exhibits a ^1^ILCT (π_epycb_ → π_epycb_^*^) character.

**Figure 6:**
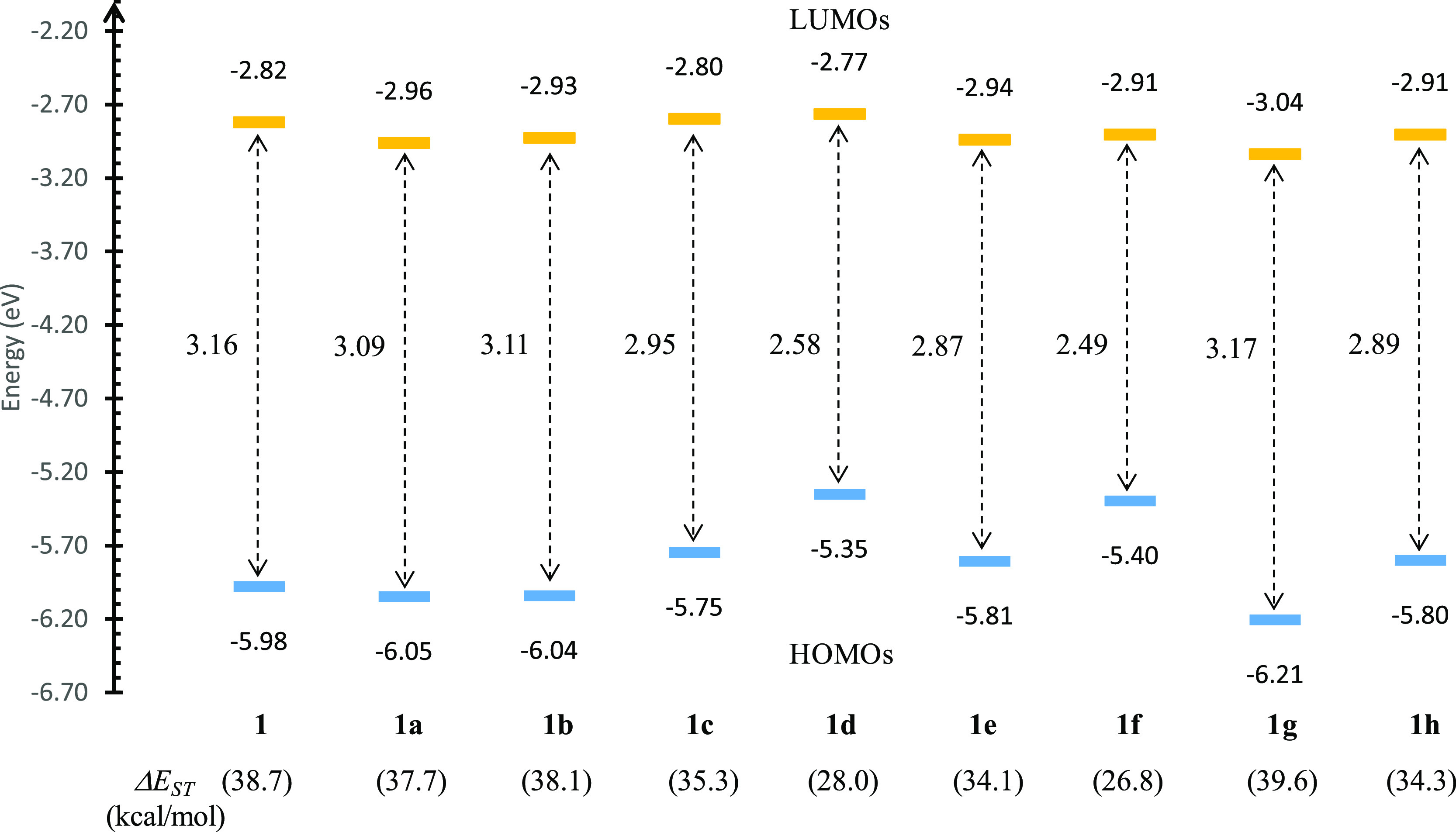
Energy diagram of the most relevant KSOs for Re(I) complexes **1a–1h** in their singlet ground states along with their corresponding HOMO–LUMO energy gaps. The difference in electronic energy between their triplet excited states (T_1_) and their singlet ground states (S_0_), Δ*E*_ST_, is given in parentheses. For comparison purposes, data obtained for complex **1** are also included.

To gain insights into the effect of the substituents in λ_max_, Figure 6 reflects the energy of the HOMO and LUMO orbitals in complexes **1a–h**. In all cases, the lowest-lying absorption band is mainly described by a HOMO → LUMO transition, so the energy gap between these orbitals (Δ*E*_H→L_) is also included.

According to our results, the relationship between λ_max_ and Δ*E*_H→L_ is confirmed as large energy gaps are always associated to low λ_max_ values. In this sense, the energy of the HOMO is mostly affected by the introduction of donor substituents in the indole moiety of the bidentate ligand. Thus, the energies of the HOMO in **1a** and **1b** (−6.05 and −6.04 eV, respectively) are very close to that of complex **1** (−5.98 eV), whereas this energy increases to, approximately, −5.8 eV with the inclusion of the OMe group (**1c**, **1e**, and **1h**) and to −5.4 eV with NMe_2_ (**1d** and **1f**), demonstrating again the stronger effect of this last electron-donating group. Accordingly, in the case of complex **1g**, where the CO_2_Et substituent at the indole ring has an electron-withdrawing effect, the HOMO is stabilized to −6.21 eV. Therefore, an electron-rich indole moiety induces a bathochromic shift on the longest-wavelength absorption band through the destabilization of the HOMO. This is a consequence of the large contribution of the indole π orbital to the HOMO of these Re complexes. On the other hand, the energy of the LUMO mainly varies upon the addition of the substituents at the fused pyridine ring of the epycb ligand. Since all the groups introduced in that ring are electron-withdrawing, the LUMO always undergoes a stabilization to, approximately, −2.9 eV, whereas it remains close to the −2.82 eV value of compound **1** in the complexes without such substituents (**1c** and **1d**). Compared to the variations in the energy of the HOMO, which are as large as 0.63 eV (comparing **1** and **1d**), the energy of the LUMO is much less affected by the substituents, with the maximum variation from **1** to **1g** of 0.22 eV. This is in accordance with the larger bathochromic shifts computed upon the addition of the electron-donating groups at the indole ring.

Now, we turn our attention to the capacity of generating singlet oxygen of complexes **1a–1h**. To that end, we optimize the geometry of such complexes in their respective triplet excited states (Table S10). Comparing the triplet optimized structures of complexes **1a–1h** to their analogous singlet ones, the geometrical variations obtained are similar to those found in the case of complex **1** (Table S12). That is, the change from the singlet ground state to the triplet excited one of the substituted epycb complexes gives rise to a moderate shortening of the Re–N_indole_ and Re–N_pyridine_ bond lengths, ranging from 0.009 to 0.023 and from 0.016 to 0.019 Å, respectively. The remaining Re–ligand bond distances show small variations between −0.003 and 0.010 Å. A similar conclusion was also found when comparing the relevant bond and dihedral angles (Table S12). The spin density plots obtained for the triplet optimized geometries highlight a mixed ^3^MLCT–^3^ILCT (*d*π_Re(CO)_2__ + π_epycb_ → π_epycb_^*^) character for all the substituted epycb complexes, as that obtained for complex **1**, except **1d** and **1f**, which primarily exhibit a ^3^ILCT (π_epycb_ → π_epycb_^*^) character (Figure S6). Concerning the singlet–triplet energy gap, all complexes **1a–1h** show Δ*E*_ST_ values greater than 22.5 kcal/mol, ranging from 26.8 kcal/mol for complex **1f** to 39.6 kcal/mol for complex **1g** (Figure 6), which is enough to produce singlet oxygen. However, it has been experimentally reported that the formation of singlet oxygen is suppressed for complexes containing the strong electron-donating NMe_2_ substituent in the indole moiety.^27^ Looking at Figure 5, we reason that it is due to the fact that the contribution of the Re d orbital to the HOMO of complexes **1d** and **1f** is practically negligible, changing the nature of the first singlet excited state from a mixed ^1^MLCT–^1^ILCT character to just a ^1^ILCT one. In this sense, it has been reported that the intersystem conversion to the triplet state of the PS, which is the one that reacts with ^3^O_2_, is enhanced if the singlet excited state has a significant contribution from the metal center due to the heavy atom effect.^43,44^ This is not the case for complexes containing the NMe_2_ substituent (**1d** and **1f**). Despite this, taking into account that **1d** and **1f** have the lowest Δ*E*_ST_ values (28.0 and 26.8 kcal/mol, respectively), we do not rule out the possibility that other factors, such as several high-frequency vibrational oscillations, may also lead to the deactivation of these complexes in its triplet state before reacting with ^3^O_2_. In any case, we will no longer consider complexes with that substituent in the following studies.

### Effect of Replacing the CO trans to the Pyridine Ligand by Phosphines

**Figure 7:**
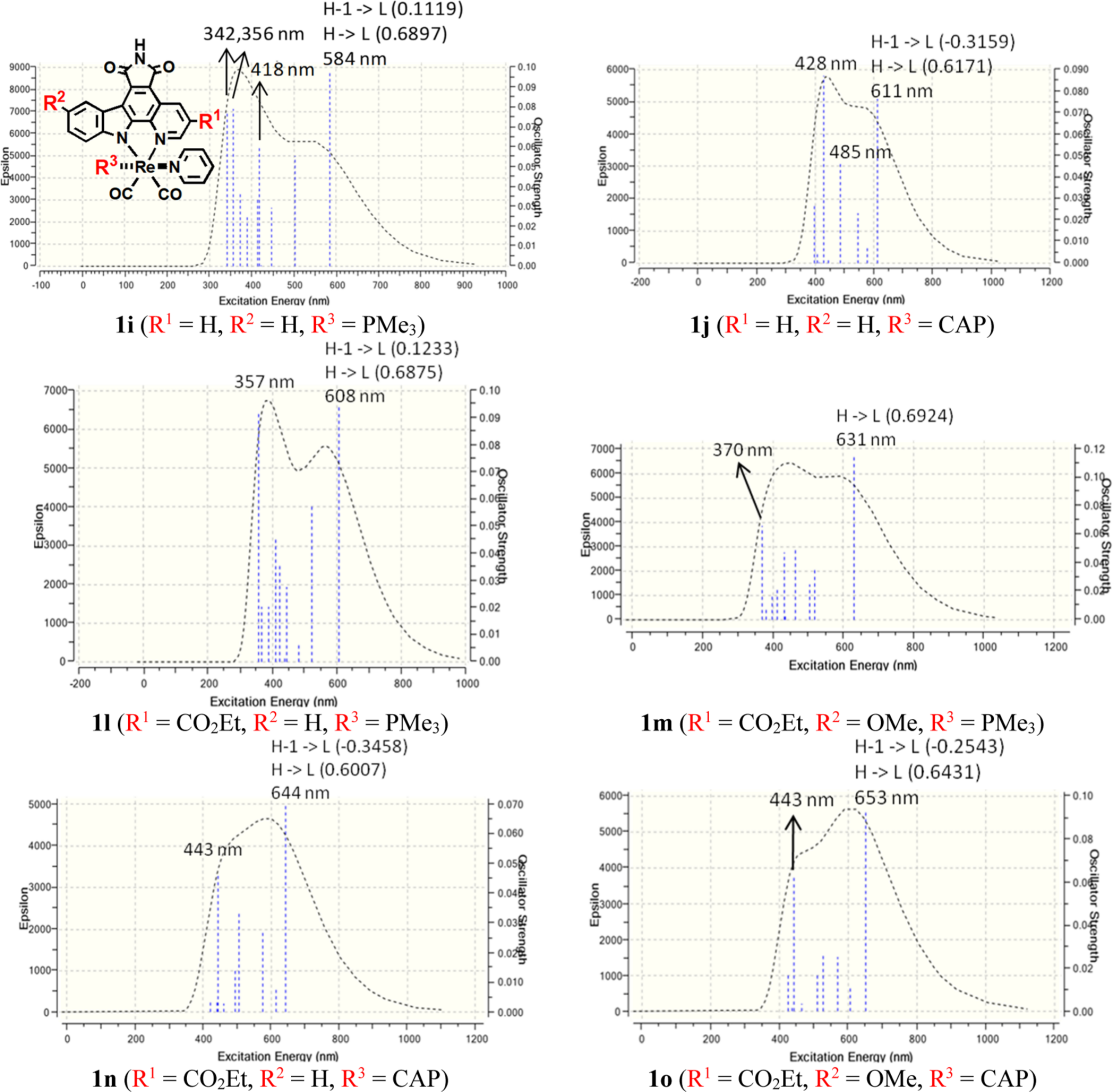
Computed electronic absorption spectra of some representative Re(I) epycb complexes bearing phosphine ligands PMe_3_ (**1i**, **1l**, and **1lm**) and CAP (**1j**, **1n**, and **1o**). The dominant orbital excitations (coefficients in parentheses) involved in the most red-shifted absorption transition are also displayed. General structures of complexes **1i–1s** are also given at the top of the left column.

Aiming at shifting the lowest-lying absorption band to even higher λ_max_ values than those of the previous complexes and thus improving their potential light-induced cytotoxicity, following the work of Dempsey et al.,^41^ we decided to study the 11 new complexes obtained by replacing the CO ligand in trans (CO_trans_) to the py one by phosphines PMe_3_, CAP, and DAPTA (complexes **1i–1s** in Figure 7 and Scheme 3). First, we replaced the CO_trans_ of complex **1** by PMe_3_ (**1i**), CAP (**1j**), and DAPTA (**1k**). Then, we introduced the well-behaved substituents tested in the previous subsection in the epycb ligand of these complexes (complexes **1l–1s**).

The optimized geometries of complexes **1i–1s** in their singlet ground states are collected in the Supporting Information (Figure S5 and Table S8). As expected, substitution of CO_trans_ with PMe_3_, CAP, or DAPTA significantly modifies the geometry of complex **1**. The least affected metal–ligand bond distances are those between Re and the N atoms of the fused pyridine and indole moieties of the bidentate ligand, which leads to increases in the gaps of 0.006–0.012 and 0.010–0.019 Å, respectively (Table S15). Substitution of the π-accepting CO_trans_ ligand with a strong σ-donating and weak π-accepting one, PMe_3_, CAP, or DAPTA, alleviates the electron deficiency of Re, thus strengthening its bond with the py ligand, which shortens to the range between 0.032 and 0.042 Å, and the well-known backbonding interactions between the filled Re d orbitals and π* orbitals of the two remaining CO ligands of complexes **1i–1s** and, consequently, shortening their Re–CO bond distances (from 0.021 to 0.026 Å).

Focusing initially on complexes **1i** (with PMe_3_) and **1j** (with CAP), their computed absorption spectra are displayed in Figure 7. As expected, the lowest-lying absorption band suffers a significant bathochromic shift when compared to that of **1**, going from a λ_max_ of 521 nm for **1** to λ_max_ values of 584 and 611 nm for **1i** and **1j**, respectively. It is remarkable that these values are even larger than the best ones obtained from the substitutions in the epycb ligand while keeping three CO ones (excluding the NMe_2_ substituents). A bathochromic shift was also observed for complex **1k** (with DAPTA) but much smaller than that of **1i** and **1j** since λ_max_ is now 559 nm, only 38 nm larger than that of **1** (Figure S7 and Table S11).

**Figure 8:**
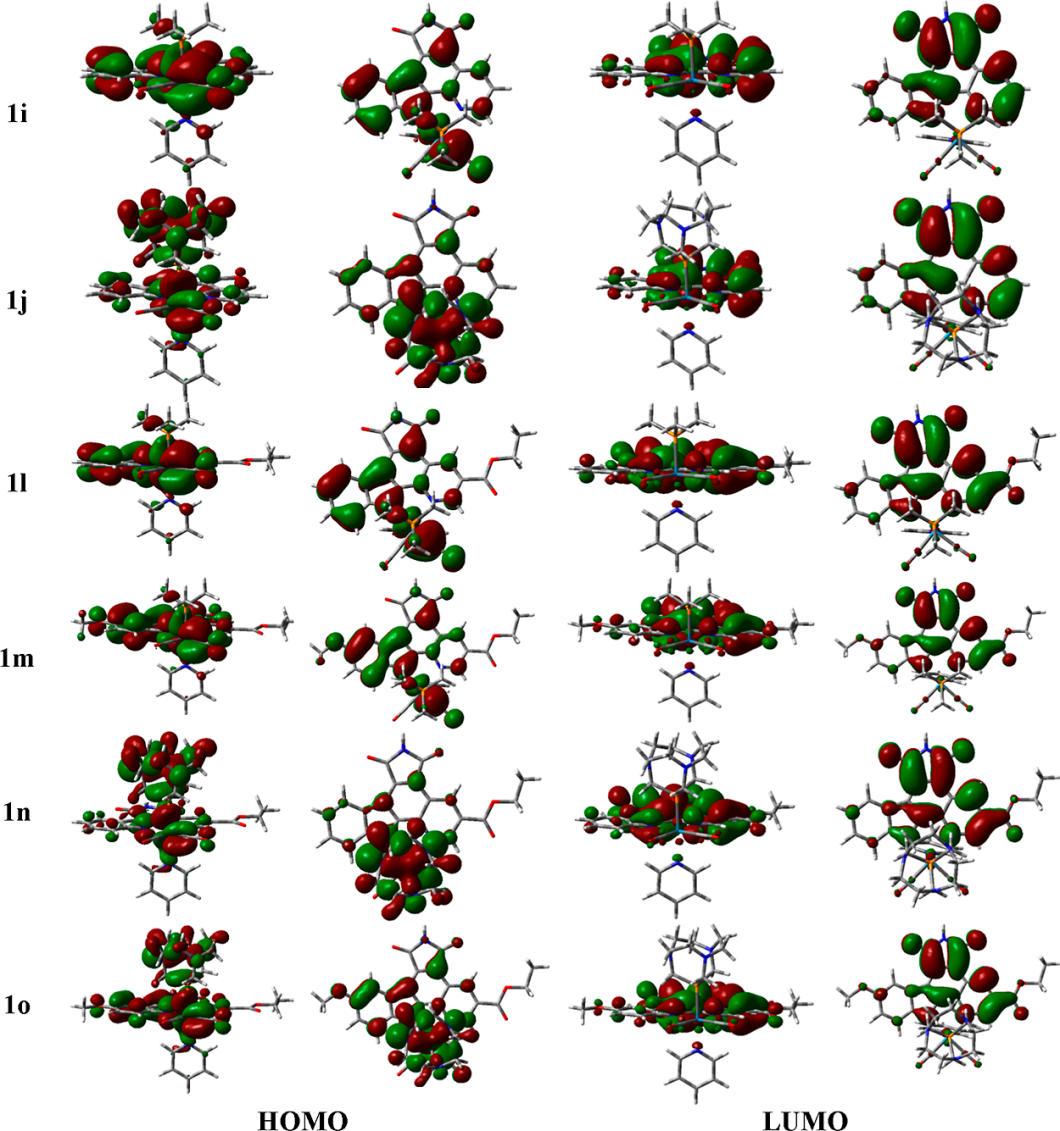
Contour maps of the frontier KSOs involved in the main orbital transition of the lowest-lying absorption band found for some representative Re(I) dicarbonyl pyridyl complexes containing phosphine ligands PMe_3_ (**1i**, **1l**, and **1m**) and CAP (**1j**, **1n**, and **1o**). Two views are given for each orbital.

**Figure 9:**
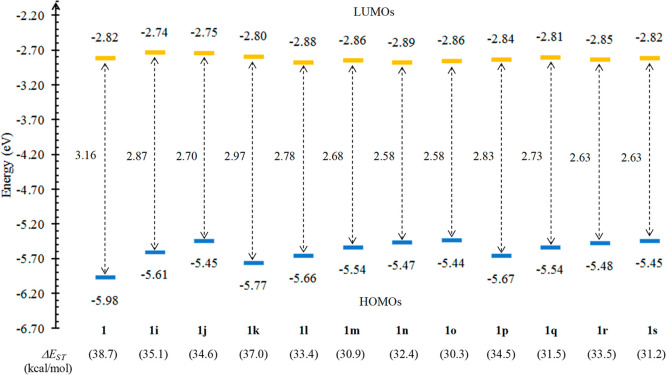
Energy diagram of the most relevant KSOs for complexes containing phosphine ligands **1i–1s** together with their corresponding HOMO–LUMO energy gaps. The difference in electronic energy between their triplet excited states (T_1_) and their singlet ground states (S_0_), Δ*E*_ST_, is given in parentheses. For comparison purposes, data obtained for complex **1** are also included.

To clarify the effect of replacing the CO_trans_ by PMe_3_, CAP, or DAPTA in the lowest-lying absorption band, which is mainly described as a HOMO → LUMO transition with certain participation of the HOMO – 1 → LUMO transition in the three complexes (Figure S7 and Table S11), the shapes of the most relevant frontier KSOs of **1i** and **1j** are shown in Figure 8 whereas the one of **1k**, which is similar to that of **1i**, is depicted in Figure S8. The energies of all these orbitals along with the HOMO–LUMO energy gap are collected in Figure 9. On the one hand, the energy and composition of the LUMO in complexes **1i–1k** are very similar to those of complex **1**, which is mainly described by a π* orbital of the epycb ligand. Therefore, the effect of the phosphine ligand in this orbital is minimal. On the other hand, the HOMO, where the replaced CO_trans_ had a relevant contribution, suffers significant changes. In the case of complexes **1i** and **1k**, it is described as a combination of a Re d orbital with a π* orbital of one of the CO ligands and a π orbital belonging to the bidentate ligand, mainly centered at the indole moiety, with no participation of the PMe_3_ and DAPTA fragments, respectively. The absence of the π* orbital of the CO_trans_ ligand, which had a stabilizing effect, increases the energy of the HOMO by 0.37 eV (complex **1i**) and 0.21 eV (complex **1k**) when compared to complex **1**. Regarding complex **1j**, its HOMO is now formed by the combination of a Re d orbital with a π* orbital of one CO ligand, a very small contribution from a π orbital of the epycb ligand, and a large one from an orbital centered on the CAP ligand. Thus, the energy of the HOMO increases to −5.45 eV (an increase of 0.53 eV with respect to that of complex **1**). As a consequence, the HOMO–LUMO transition associated with the lowest-lying absorption band of complex **1j** is now mainly described as a mixture of ^1^MLCT and ligand-to-ligand charge transfer (^1^LLCT), that is, from Re to the bidentate ligand and from the CAP one to the same bidentate ligand. In the case of complexes **1i** and **1k**, that band corresponds to a mixed ^1^MLCT–^1^ILCT (*d*π_Re(CO)_ + π_epycb_ → π_epycb_^*^) transition.

Then, complexes **1i** and **1j** were modified by varying the electron density of their epycb ligand through the addition of adequate substituents at their fused pyridine and indole moieties (complexes **1l–1s** in Figure 7 and Scheme 3). This has not been considered for complex **1k** as the effect of the DAPTA ligand on the shape of the HOMO and the LUMO is similar to that of the PMe_3_ ligand. In addition, the destabilization of the HOMO of the DAPTA complex (**1k**) is even lower than that of the PMe_3_ one (**1i**), thus inducing a lower reduction of the HOMO–LUMO energy gap (Figure 9) and, consequently, a reduced bathochromic shift. In all the complexes **1l–1s**, the main transition of the lowest-lying absorption band is HOMO → LUMO, so we pay special attention to those orbitals, although the HOMO – 1 → LUMO transition has a moderate contribution in the complexes bearing the CAP ligand (**1n**, **1o**, **1r**, and **1s**), slightly larger than that in those bearing the PMe_3_ ligand (**1l**, **1p**, and **1q**). The shapes of the HOMOs and the LUMOs are collected in Figures 8 and S8, whereas their energies are shown in Figure 9. First of all, it can be observed that the trend for the complexes bearing the electron-withdrawing substituent CO_2_Et (**1l–1o**) is the same as that for the ones with a F group (**1p–1s**), although the effect of the former substituent on λ_max_ is slightly larger due to a larger stabilization of the LUMOs, in accordance with the results from the previous subsection. Therefore, for the sake of simplicity, we focus our discussion on complexes **1l–1o**.

Regarding the complexes with the PMe_3_ ligand, the effect of adding one CO_2_Et group to the fused pyridine ring of the epycb ligand (complex **1l**) is, as expected, a stabilization of the LUMO of around 0.14 eV, similar to the one observed when comparing complex **1** with **1a** (the corresponding complex with a CO ligand instead of PMe_3_). However, the increase in the energy of the HOMO upon the introduction of the electron-donating OMe substituent in the indole moiety (complex **1m**) is only 0.07 eV when comparing complexes **1m** and **1i**, quite smaller than the difference between the HOMO energy of complexes **1** and **1e** (0.17 eV). This is mainly due to the lower contribution of the π_epycb_ orbital to the HOMO in the complexes with PMe_3_. Nevertheless, the red shift induced to the absorption band in both complexes is significant, reaching λ_max_ values greater than 600 nm.

For complexes bearing the CAP ligand (**1n** and **1o**), the energies of their LUMOs are identical to those of the analogous PMe_3_ complexes, as the LUMO in all of them is always a π* orbital of the epycb ligand. Again, the energy of the HOMO does not suffer variations of more than 0.02 eV when comparing complex **1j** with **1n** and **1o**. The reason for that is again the almost negligible contribution of the epycb ligand, where substituents are placed, to their HOMOs. Therefore, the main reason for the 20 and 33 nm lengthening of the longest λ_max_, with respect to **1j**, in complexes **1n** and **1o**, respectively, is the stabilization of the LUMO caused by the CO_2_Et group, although the minor effect of the OMe group on the HOMO (and the HOMO – 1) also contributes to make the red shift even larger.

As for complexes **1–3** and **1a–1h**, we have also examined the ability of phosphine complexes **1i–1s** to promote the formation of singlet oxygen. To accomplish this task, we optimized the geometry of such complexes in their respective triplet excited states (Table S10). Comparing the triplet optimized structures of complexes **1i–1s** to their corresponding singlet ones (Table S12), we found a notable shortening of the distance between Re and the N atom of the py ligand from 0.050 to 0.079 Å, whereas a moderate lengthening (0.017–0.034 Å) was obtained for the distance between Re and the CO located on the same side as that of the fused pyridine moiety of the epycb ligand. A small shortening (0.009–0.015 Å) was found for the distance between Re and the N atom of the bidentate pyridine moiety. The remaining metal–ligand bond lengths of the phosphine complexes hardly change when going from the singlet species to the triplet ones. As for the complexes investigated in the previous subsections, the nature of the triplet state for complexes **1i–1s** on the basis of the spin density distributions obtained (Figure S6) is similar to that of the corresponding singlet ground state, that is, a mixed ^3^MLCT–^3^ILCT character for PMe_3_ and DAPTA complexes and a mixed ^3^MLCT–^3^LLCT character for CAP complexes. Yet again, as displayed in Figure 9, all the phosphine complexes show Δ*E*_ST_ values greater than 22.5 kcal/mol, ranging from 30.3 kcal/mol for complex **1o** to 35.1 kcal/mol for complex **1i**. This, together with the fact that complexes **1m** (R^1^ = CO_2_Et, R^2^ = OMe, and R^3^ = PMe_3_), **1n** (R^1^ = CO_2_Et, R^2^ = H, and R^3^ = CAP), **1o** (R^1^ = CO_2_Et, R^2^ = OMe, and R^3^ = CAP), **1q** (R^1^ = F, R^2^ = OMe, and R^3^ = PMe_3_), **1r** (R^1^ = F, R^2^ = H, and R^3^ = CAP), and **1s** (R^1^ = F, R^2^ = OMe, and R^3^ = CAP) present light absorption in the therapeutic window (∼620–850 nm), leads us to propose them as the preferred PS candidates for their use in PDT. Nonetheless, it is also likely that the remaining phosphine complexes as well as the substituted epycb complexes investigated in the previous subsection, **1a–1h**, except those containing the NMe_2_ substituent, could be used as PSs for PDT since they absorb at λ_max_ values longer than that observed for complex **1**, whose photocytotoxicity was detected experimentally at λ ≥ 505 nm.^26^

## Conclusions

Starting from complex [Re(epycb)(CO)_3_(py)] (epycb = pyrido[2,3-*a*]pyrrolo[3,4-*c*]carbazole-5,7(6*H*)-dione, py = pyridine), several issues have been analyzed through the density functional theory (DFT) and time-dependent DFT (TD-DFT) methodologies to rationalize their effect on the spectroscopic and photocytotoxic properties of this complex. These include the size of the pyridocarbazole-type bidentate ligand epycb and the addition of substituents to its rings as well as of the replacement of the carbonyl ligand trans (CO_trans_) to the py one by phosphines. First, we have found that, similar to the closely related Re(I) complexes previously reported, the greater the number of conjugated rings in the bidentate ligand is, the greater the bathochromic shift is. This is caused by a reduction in the energy gap corresponding to the HOMO (or HOMO – 1) → LUMO transition, which has a mixed ^1^MLCT–^1^ILCT (*d*π_Re(CO)_2__ + π_bidentate_ → π_bidentate_^*^) character. More interestingly, the electron-withdrawing 1*H*-pyrrole-2,5-dione heterocycle added to the bidentate ligand plays an important role by enhancing even more the stabilization of the LUMO. Second, we have seen that, taking into account the shape of the KSOs involved in the lowest-lying absorption band of the reference complex, the introduction of electron-withdrawing substituents into the fused pyridine ring of the epycb ligand mainly stabilizes the LUMO, whereas the HOMO destabilizes primarily with electron-donating substituents in the epycb indole moiety. Both types of substituents, in an isolated fashion, result in a bathochromic shift of the most red-shifted absorption band of the reference complex, which is even larger if they are combined in the same compound. Yet again, such bathochromic shifts are due to a diminution in the energy gap of the HOMO → LUMO transition characterized as ^1^MLCT–^1^ILCT (*d*π_Re(CO)_2__ + π_bidentate_ → π_bidentate_^*^) for all the substituted epycb complexes except those containing the NMe_2_ substituent, which only show a ^1^ILCT character (π_bidentate_ → π_bidentate_^*^). This fact, along with a low Δ*E*_ST_ value, could explain the absence of photocytotoxicity experimentally found for analogous complexes (with the NMe_2_ group) bearing imidazole ligands. Finally, we have found that, except for complexes with the NMe_2_ substituent, the replacement of CO_trans_ by PMe_3_ or CAP induces a greater bathochromic shift than the introduction of substituents into the epycb ligand due to the loss of one Re–CO backbonding interaction. Although this interaction is also absent when CO_trans_ is replaced by DAPTA, this phosphine renders a lower bathochromic shift. The extra red shift found for CAP complexes compared to the PMe_3_ ones is a consequence of the participation of CAP in the HOMO. Consequently, the HOMO → LUMO transition, mostly responsible for the bathochromic shifts found in phosphine complexes, goes from a ^1^MLCT–^1^ILCT character (*d*π_Re(CO)_ + π_bidentate_ → π_bidentate_^*^) for PMe_3_ and DAPTA complexes to a ^1^MLCT–^1^LLCT character (*d*π_Re(CO)_ + CAP → π_bidentate_^*^) for CAP complexes. By combining the CAP ligand with electron-withdrawing and/or electron-donating substituents at the epycb ligand, we have found several complexes with significant absorption at the therapeutic window. In addition, the singlet–triplet energy gap obtained for those complexes is greater than 30.0 kcal/mol, clearly above the minimum energy required, 22.5 kcal/mol, to transform triplet oxygen into singlet oxygen, indicating their capacity to photosensitize cytotoxic oxygen. Therefore, these new complexes present very interesting features that make them promising compounds for PDT.

## Computational Details

The TD-DFT methodology^45,46^ has proven to be reliable for studying UV–vis absorption spectra of Re(I) carbonyl complexes with α-diimine-type ligands.^33,47−76^ A variety of DFT methods together with different basis sets and without or with including solvent effects have been used for that purpose. As TD-DFT computations require the geometry optimization of the stable species first and then the measurement of their corresponding electronic absorption spectra,^77,78^ we describe and justify the levels of theory employed in the present work below.

### Geometry Optimization

The ground-state geometry of all the Re(I) complexes investigated in this work was optimized in the gas phase using the popular hybrid B3LYP functional^79−82^ and corrected with Grimme’s D3 dispersion^83^ in conjunction with Pople’s 6-31+G(d) basis set for nonmetal atoms^84^ and the valence double-ζ basis set LANL2DZ plus the effective core potential of Hay and Wadt for the Re atom.^85^ The location of the critical points on the potential energy surface was carried out using a modified Schlegel algorithm.^86−88^ The nature of the optimized species as global minima was corroborated by means of an analytical calculation of harmonic vibrational frequencies. B3LYP has often been used in conjunction with double-ζ quality basis sets in order to obtain successful optimized geometries of rhenium carbonyl complexes aiming at carrying out TD-DFT calculations.^47−55,57,67,69,70,74,89−91^ The inclusion of dispersion correction is also important to improve the performance of the B3LYP/6-31+G(d)-LANL2DZ level in yielding better optimized geometries. Despite all of this, we compared the B3LYP-D3/6-31+G(d)-LANL2DZ optimized geometry of a Re(I) complex closely related to those investigated in this work, a N-benzylated derivative of the Re(I) tricarbonyl complex containing a pyridine ligand and another pyrido[2,3-*a*]pyrrolo[3,4-*c*]carbazole-5,7(6*H*)-dione ligand, with that reported by X-ray diffraction data.^26^ The differences between both geometries show a mean absolute deviation (root mean square deviation) in the bond distances and bond angles involving non-hydrogen atoms of 0.011 Å (0.014 Å) and 0.65° (1.03°), respectively. These relatively small discrepancies corroborate the computational protocol chosen (see the Discussion 1 section in the Supporting Information).

The B3LYP-D3/6-31+G(d)-LANL2DZ level of theory was also employed to optimize all the Re(I) complexes in the first triplet excited state, aiming at determining the difference in the electronic energy between the triplet excited state and the singlet ground state. To that, the singlet and triplet B3LYP-D3/6-31+G(d)-LANL2DZ energies obtained for each complex were refined at the level used in the TD-DFT computations (see below).

### Electronic Absorption Spectrum

The electronic absorption properties of all the Re(I) complexes were investigated by performing PCM-TD-M06/6-31+G(d)-LANL2DZ calculations on the B3LYP-D3/6-31+G(d)-LANL2DZ optimized geometries. That is, TD-DFT computations were carried out using the hybrid meta-GGA M06^92^ along with the same basis set as the one used for geometry optimization. Solvent effects of DMSO, the one used in the UV–vis absorption spectra of the Re(I) complexes **1–3** and derivatives (see Scheme 1),^26^ were simulated by means of the polarizable continuum model (PCM) taking into account the electrostatic, cavitation, dispersion, and repulsion terms for the evaluation of the total energy in solution.^93−99^ Only the first 10 lowest excitation energies were considered in these computations as we are mainly interested in the lowest-lying absorption band. Apart from the PCM-TD-M06/6-31G(d)-LANL2DZ level, we also considered other computational levels in the TD-DFT computations by replacing M06 by GGA (PBE^100,101^), meta-GGA (TPSS^102^ and wB97x^103^), hybrid GGA (B3LYP-D3,^79−83^ PBE0,^104^ and wB97xD^105^), hybrid meta-GGA (M05,^106^ MN15,^107^ and TPSSh^102,108^), and long-range separate (CAM-B3LYP^109^) functionals. These 11 functionals have been chosen after an extensive revision of the computational protocols commonly used to theoretically investigate the UV–vis properties of Re(I) carbonyl complexes. As explained in the Discussion 2 section in the Supporting Information, our results show that M06 is the most satisfactory functional to reproduce the experimental absorption spectra reported for complexes **1–3**. Thus, a similar behavior should be expected when dealing with Re(I) carbonyl pyridylcarbazole complexes containing small alterations.

All the quantum chemical calculations were performed with the Gaussian 16 (G16) suite of programs.^110^

## Supporting Information Available

Full DFT and TD-DFT geometry and energy details on all the Re(I) complexes investigated and the discussion on the validation of the computational protocol used in this work (PDF)
