# Supplementary material for: Computational Design of Rhenium(I) Carbonyl Complexes for Anticancer Photodynamic Therapy
Source: Inorg Chem. 2021 Dec 16;61(1):439–55. doi: 10.1021/acs.inorgchem.1c03130 (PMC8753654; doi:10.1021/acs.inorgchem.1c03130)
Supplement: Supplementary file 1 — ic1c03130_si_001.pdf [file ic1c03130_si_001.pdf]

Supporting Information

Computational Design of Rhenium(I) Carbonyl Complexes for Anticancer Photodynamic Therapy

Daniel Álvarez,[†] M. Isabel Menéndez,[†] and Ramón López[*†]

[†]Departamento de Química Física y Analítica, Facultad de Química, Universidad de Oviedo, C/ Julián Clavería 8, 33006 Oviedo, Spain

***Corresponding author**

Email address: rlopez@uniovi.es – Tel. +34 985 10 29 67

Discussion 1. Validating the computational protocol for geometry optimizations. S4
Discussion 2. Validating the computational protocol for TD-DFT calculations. S6
Table S1. Cartesian coordinates of the X-ray and B3LYP-D3/6-31+G(d)-LANL2DZ structures for the Re(I) complex $\mathbf{1}^{\mathbf{ref}}$. S9
Figure S1. Two comparative views for the X-ray and B3LYP-D3/6-31+G(d)-LANL2DZ structures of the Re(I) complex $\mathbf{1}^{\mathbf{ref}}$. S10
Table S2. X-Ray and B3LYP-D3/6-31+G(d)-LANL2DZ bond distances of the Re(I) complex $\mathbf{1}^{\mathbf{ref}}$ along with absolute difference and square of the absolute difference of these distances. S11
Table S3. X-Ray and B3LYP-D3/6-31+G(d)-LANL2DZ bond angles of the Re(I) complex $\mathbf{1}^{\mathbf{ref}}$ together with absolute difference and square of the absolute difference of these angles. S13
Table S4. Excitation energies and oscillator strengths of the first ten lowest-lying singlet-singlet electron transitions calculated for complex **1**. S15
Table S5. Excitation energies and oscillator strengths of the first ten lowest-lying singlet-singlet electron transitions calculated for complex **2**. S16
Table S6. Excitation energies and oscillator strengths of the first ten lowest-lying singlet-singlet electron transitions calculated for complex **3**. S17
Figure S2. Electronic spectra computed for complex **1**. S18
Figure S3. Electronic spectra computed for complex **2**. S20
Figure S4. Electronic spectra computed for complex **3**. S22
Table S7. Excitation energy of the most red-shifted absorption band found for complexes **1**-**3** along with the difference in absolute value between the computed excitation energies and the experimental value and the total error obtained. S24
Table S8. B3LYP-D3/6-31+G(d)-LANL2DZ relevant optimized geometry data for Re(I) complexes **1**-**3** and **1a**-**1s** in their singlet ground states. S25
Table S9. PCM-TD-M06/6-31+G(d)-LANL2DZ//B3LYP-D3/6-31+G(d)-LANL2DZ excitation energies and oscillator strengths of the first ten lowest-lying singlet-singlet electron transitions for Re(I) complexes **1**-**3**. S26
Table S10. B3LYP-D3/6-31+G(d)-LANL2DZ relevant optimized geometry data for Re(I) complexes **1**-**3** and **1a**-**1s** in their triplet excited states. S27
Table S11. PCM-M06/6-31+G(d)-LANL2DZ//B3LYP-D3/6-31+G(d)-LANL2DZ energies in the singlet ground state and triplet excited state and difference between them, excitation energy, oscillator strength, dominant orbital excitations for the lowest-lying absorption band, HOMO and LUMO energies, and difference between them for Re(I) complexes **1**-**3** and **1a**-**1s**. S38

Table S12. B3LYP-D3/6-31+G(d)-LANL2DZ variations of some relevant bond distances, bond angles, and dihedral angles when going from the singlet ground state to the corresponding first excited state of Re(I) complexes **1**-**3** and **1a**-**1s**. S39

Figure S5. B3LYP-D3/6-31+G(d)-LANL2DZ singlet ground-state optimized geometries of Re(I) complexes **1a**-**1s**. S30

Figure S6. Contour maps of the spin density distribution for the triplet excited state of the Re(I) complexes **1a**-**1s**. For clarity, two views are given for each species. S35

Table S13. B3LYP-D3/6-31+G(d)-LANL2DZ variations of relevant bond distances, bond angles, and dihedral angles between complexes **1a-1h** and **1**. S40

Table S14. PCM-TD-M06/6-31+G(d)-LANL2DZ//B3LYP-D3/6-31+G(d)-LANL2DZ excitation energies and oscillator strengths of the first ten lowest-lying singlet-singlet electron transitions for Re(I) complexes **1a**-**1h**. S41

Tables S15. B3LYP-D3/6-31+G(d)-LANL2DZ variations of relevant bond distances, bond angles, and dihedral angles between complexes **1i-1s** and **1**. S42

Table S16. PCM-TD-M06/6-31+G(d)-LANL2DZ//B3LYP-D3/6-31+G(d)-LANL2DZ excitation energies and oscillator strengths of the first ten lowest-lying singlet-singlet electron transitions for Re(I) complexes **1i**-**1s**. S43

Figure S7. PCM-TD-M06/6-31+G(d)-LANL2DZ//B3LYP-D3/6-31+G(d)-LANL2DZ electronic absorption spectra of Re(I) complexes bearing the phosphine ligands DAPTA (**1k**), PMe_3 (**1p** and **1q**), and CAP (**1r** and **1s**). S44

Figure S8. Contour maps of the frontier Kohn-Sham orbitals involved in the main orbital transition of the lowest-lying absorption band found for Re(I) complexes containing the phosphine ligands DAPTA (**1k**), PMe_3 (**1p** and **1q**) CAP (**1r** and **1s**). S45

Table S17. B3LYP-D3/6-31+G(d)-LANL2DZ cartesian coordinates of the optimized structures in the singlet ground state of Re(I) complexes **1**-**3** and **1a**-**1s**. S46

Table S18. B3LYP-D3/6-31+G(d)-LANL2DZ cartesian coordinates of the optimized structures in the triplet excited state of Re(I) complexes **1**-**3** and **1a**-**1s**. S56

References related to Discussion 1. S66

References related to Discussion 2. S70

Discussion 1. Validating the computational protocol for geometry optimizations.

The geometry of all the Re(I) carbonyl complexes investigated in this work was optimized in the gas phase at the B3LYP-D3/6-31+G(d)-LANL2DZ level of theory. Although this computational level was chosen on the basis of numerous theoretical investigations on the photophysical and spectroscopic properties of Re(I) carbonyl complexes,[1-39] we checked its performance for geometry optimizations by comparing the X-ray structure of a *N*-benzylated derivative of the Re(I) tricarbonyl complex containing pyridyl and pyridocarbazol ligands (**1**^{ref} in Scheme S1)[40] with its B3LYP-D3/6-31+G(d)-LANL2DZ optimized structure. Complex **1**^{ref} is closely related to the Re(I) carbonyl indolato complexes studied in the present investigation and furthermore its X-ray structure is available.[40] Cartesian coordinates of both structures are collected in Table S1.

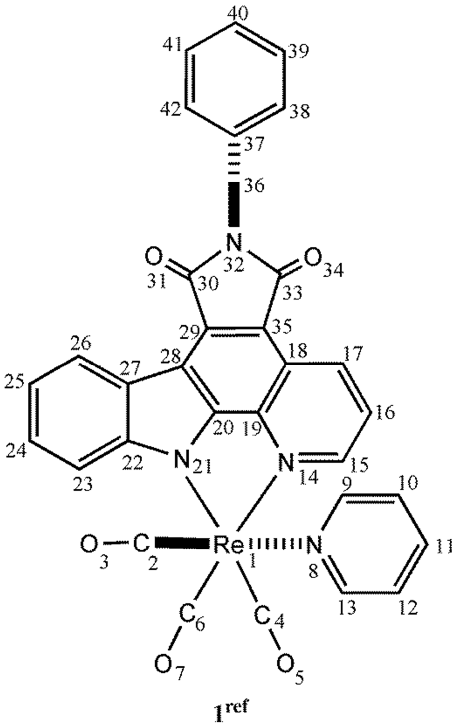

1^{ref}

Scheme S1. *N*-benzylated derivative of the Re(I) tricarbonyl complex containing a pyridine ligand and another pyrido[2,3-*a*]pyrrolo[3,4-*c*]carbazole-5,7(6*H*)-dione ligand considered in the validation calculations of the B3LYP-D3/6-31+G(d)-LANL2DZ level for geometry optimizations. Atom numbering of the non-hydrogen atoms, the most relevant ones, is included.

Figure S1 shows that the main difference between X-ray (in blue colour) and B3LYP-D3/6-31+G(d)-LANL2DZ (in red colour) structures lies in the orientation of the benzyl

group attached to the nitrogen atom N32 of the pyridocarbazol ligand (Scheme S1), which can be ascribed to the crystal packing of $\mathbf{1}^{\mathbf{ref}}$. Specifically, as collected in Table S2, we note that the absolute difference between X-ray and B3LYP-D3 bond distances involving non-hydrogen atoms in $\mathbf{1}^{\mathbf{ref}}$ varies from 0.0002 Å (C27-C28 bond distance) to 0.048 Å (Re1-N8 bond distance). The mean absolute deviation (MAD) and the root mean square deviation (RMSD) between all those X-ray and B3LYP-D3 bond distances are 0.011 and 0.014 Å, respectively. When comparing X-ray and B3LYP-D3 bond angles involving non-hydrogen atoms in $\mathbf{1}^{\mathbf{ref}}$ (see Table S3), the absolute discrepancy between them ranges from 0.020º (Re1-N21-C20 bond angle) to 3.9º (C2-Re1-N8 bond angle). MAD of all the differences in those bond angles is 0.65º, whereas the RMSD value is about 1.0º. The relatively small RMSD value obtained for both bond distances (less than 0.02 Å) and bond angles (less than 2º)[41-43] confirms the adequacy of using the B3LYP-D3/6-31+G(d)-LANL2DZ optimized geometries to carry out Time-Dependent Density Functional Theory (TD-DFT) calculations.

Discussion 2. Validating the computational protocol for TD-DFT calculations.

Electronic excitation energies and oscillator strengths of the Re(I) carbonyl complexes were computed by performing TD-DFT calculations on the B3LYP-D3/6-31+G(d)-LANL2DZ optimized geometries. To predict a reliable level of theory for studying the excited-state properties of the Re(I) complexes investigated in this work, different density functionals (B3LYP-D3,[1-4] CAM-B3LYP,[5] M05,[6] M06,[7] MN15,[8] PBE,[9,10] PBE0,[11] TPSS,[12] TPSSh,[12,13] wB97x,[14] wB97xD)[15] were employed along with the same basis set (6-31+G(d)-LANL2DZ) as the one used for geometry optimizations in gas phase. Bulk solvent effects (dimethyl sulfoxide, DMSO, ε = 46.826) were taken into account with the polarizable continuum model (PCM) developed by Tomasi and coworkers[16-22] using the integral equation formalism variant (IEF)[20] and the universal force field (UFF) radii.[23] In these PCM-TD-DFT computations, the solute-solvent electrostatic interaction energy and the non-electrostatic terms due to the solute cavitation energy[24] as well as to the solute-solvent dispersion[25,26] and repulsion[25,26] interaction energies were considered.

Among the TD-DFT computational levels mentioned above, the most reliable was chosen by comparing the theoretical electronic spectra obtained for complexes **1-3** (see Scheme S2) with those measured experimentally in DMSO for the same complexes.[27] Tables S4-S6 collect excitation energies (both in eV, *E*, and in nm, λ) and oscillator strengths (*f*) of the first ten lowest-lying singlet-singlet electron transitions for complexes **1-3**, respectively. Figures S2-S4 display the corresponding simulated absorption spectra obtained through TD-DFT calculations and, for comparison purposes, the respective experimental spectra reported are also included.[27]

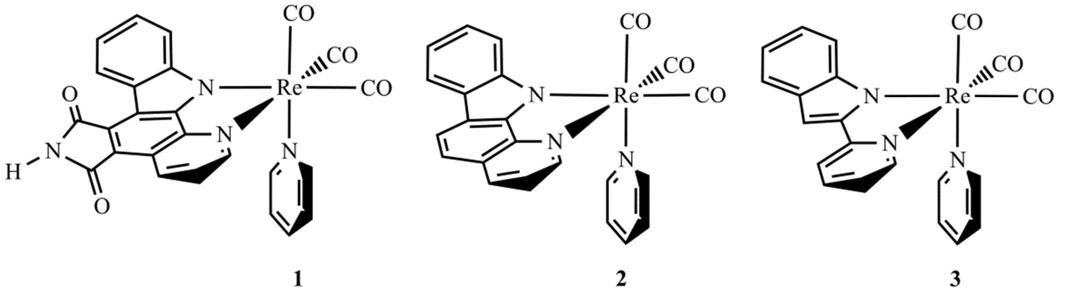

Scheme S2. Rhenium(I) tricarbonyl complexes used for validating the computational protocol employed in the TD-DFT computations.

As seen in Figure S2, B3LYP-D3, PBE, TPSS, and TPSSh provide UV/Vis absorbance spectra for **1** in Scheme S2 that differ greatly from the experimental one. The B3LYP-D3 spectrum shows three absorption bands as in the experimental one, but the sequence of the first and third intensities is erroneously predicted. Specifically, the most red-shifted absorption band is more intense than the least one, while the reverse trend was found experimentally. For PBE, TPSS, and TPSSh, the simulated UV/Vis spectra do not clearly show the presence of three absorption bands, nor at least the two most intense ones. However, the general shape of the UV/Vis spectrum of **1** is acceptably well-reproduced with the remaining functionals (CAM-B3LYP, M05, M06, MN15, PBE0, wB97x, wB97xD), although we note that the intermediate absorption band, the experimentally less intense, does not reproduce sharply. For complexes **2** and **3** in Scheme S2 (see Figures S3 and S4, respectively), the UV/Vis spectra predicted by all the functionals fit reasonably well the main features of those reported experimentally.

In addition to the aforementioned and since we are interested in Re(I) carbonyl complexes that absorb in the red region of Vis spectrum, let us turn our attention to the excitation energy of the most red-shifted absorption band. Table S7 collects the excitation energy of the most red-shifted absorption band found for complexes **1**-**3** at the levels of theory PCM-TD-DFT/6-31+G(d)-LANL2DZ//B3LYP-D3/6-31+G(d)-LANL2DZ (DFT = B3LP-D3, CAM-B3LYP, M05, M06, MN15, PBE, PBE0, TPSS, TPSSh, wB97x, wB97xD). For each computational level and each complex, the choice of that excitation energy is based on the position and intensity of the most red-shifted absorption wavelength that fits better the one found experimentally. In addition, we also show in Table S7 the absolute error obtained when comparing the computed excitation energies with the respective experimental value for each complex. For **1** (sixth column in Table S7), the excitation energy obtained with M05, PBE, PBE0, M06, and TPSS only differs, in absolute value, 0.01, 0.03, 0.03, 0.04, and 0.04 eV from the experimental one, respectively. B3LYP-D3 and MN15 give similar absolute errors (0.15 and 0.17 eV, respectively) and clearly larger than the previous functionals. The remaining DFT methods provide discrepancies larger than 0.25 eV, thus preventing their use to predict reliable UV/Vis spectra. In the case of **2** (seventh column in Table S7), we found that M06, PBE0, and M05 with absolute discrepancies of 0.003, 0.04, and 0.06 eV are the most adequate to fit the excitation energy of the experimental most red-shifted absorption band. We note, however, that M06 behaves better than PBE0 and M05. It

then follows B3LYP-D3 that only differs by 0.09 eV (in absolute value) from the experimental excitation energy. All the others functionals investigated present absolute errors larger than 0.23 eV. Concerning **3** (eighth column in Table S7), the absolute error in the excitation energy predicted by M06 (0.003 eV), MN15 (0.01 eV), B3LYP-D3 (0.02 eV), M05 (0.07 eV), PBE0 (0.09 eV), and TPSSh (0.09 eV) is very small. Yet again, M06 presents the best behavior. The remaining functionals provide absolute errors larger than 0.20 eV. Therefore, looking at the Re(I) complexes **1**-**3**, M06 is the best functional in predicting the shortest excitation energy (longest maximum absorption wavelength) by far. In fact, M06 shows a total absolute error of 0.04 eV (tenth column in Table S7). M05 and PBE0 are the following functionals in level of accuracy with total absolute errors of 0.14 and 0.16 eV, respectively. By contrast, wB97xD and wB97x present the largest total absolute errors, 1.18 and 1.80 eV, respectively. In addition, M06 is also one of the best functionals in reproducing the general shape of the UV/Vis absorption spectrum for complexes **1**-**3**. So, we conclude that M06 presents the best performance in order to investigate the spectroscopic properties of the Re(I) pyridocarbazole complexes investigated in the present work.

Table S1. Cartesian coordinates in Å of the X-ray and B3LYP-D3/6-31+G(d)-LANL2DZ structures of the Re(I) complex **1**^{ref} in Scheme S1.

X-ray

Re	3.038420	-0.058888	-0.265847
C	3.535776	0.375848	-2.075615
O	3.863610	0.645517	-3.135962
C	4.495904	-1.306106	-0.211826
O	5.350855	-2.081941	-0.173836
C	4.191813	1.350855	0.320948
O	4.855635	2.233905	0.657825
N	2.428574	-0.602756	1.788922
C	2.518414	-1.871854	2.223074
C	2.140792	-2.268715	3.491806
C	1.652408	-1.306611	4.364653
C	1.574323	-0.003425	3.943065
C	1.961428	0.313853	2.644454
N	1.498240	-1.570341	-0.781402
C	1.639880	-2.851993	-1.067144
C	0.532435	-3.679133	-1.332490
C	-0.739324	-3.180912	-1.291864
C	-0.931463	-1.818743	-1.004949
C	0.227993	-1.052108	-0.749190
C	0.117958	0.326991	-0.491826
N	1.201537	1.103941	-0.278939
C	0.714524	2.401197	-0.186289
C	1.423197	3.590294	-0.031499
C	0.711488	4.781917	-0.022246
C	-0.685170	4.797676	-0.147791
C	-1.405002	3.632018	-0.296708
C	-0.712055	2.413394	-0.324791
C	-1.093932	1.038582	-0.530372
C	-2.246571	0.264046	-0.788660
C	-3.675936	0.663008	-0.908696
O	-4.169751	1.758226	-0.817358
N	-4.377231	-0.509930	-1.161323
C	-3.526526	-1.619820	-1.242270
O	-3.900552	-2.755761	-1.469383
C	-2.163174	-1.106733	-0.995779
C	-5.814301	-0.547212	-1.383646
C	-6.640685	-0.283952	-0.149343
C	-7.993106	-0.001801	-0.296284
C	-8.795407	0.181166	0.822572
C	-8.261417	0.099215	2.088714
C	-6.929343	-0.156893	2.244442
C	-6.114215	-0.353377	1.131402
H	2.859334	-2.528070	1.628513
H	2.213474	-3.175802	3.759096
H	1.376394	-1.547931	5.241992
H	1.259553	0.675539	4.530310
H	1.890964	1.215297	2.354408
H	2.513668	-3.226291	-1.094246
H	0.671612	-4.595521	-1.542236
H	-1.484548	-3.747302	-1.455918
H	-2.350217	3.654546	-0.379227
H	-1.145370	5.628422	-0.129216
H	1.180063	5.602143	0.070207
H	2.367972	3.585813	0.065012
H	-6.058105	-1.437095	-1.743489
H	-8.369752	0.067115	-1.164519
H	-9.721092	0.363121	0.712911
H	-8.815780	0.222642	2.850195
H	-6.555720	-0.202060	3.116720
H	-5.188754	-0.535302	1.251886
H	-6.045687	0.127377	-2.070046

B3LYP-D3/6-31+G(d)-LANL2DZ

Re	3.040103	-0.242807	-0.178800
C	3.669845	0.001476	-1.987624
O	4.025991	0.146154	-3.081539
C	4.316317	-1.657436	0.119553
O	5.044850	-2.547094	0.310089
C	4.275556	1.080273	0.473025
O	4.997925	1.891145	0.888496
N	2.099165	-0.514888	1.857485
C	1.921324	-1.740486	2.392311
C	1.281818	-1.945750	3.610858
C	0.804432	-0.840493	4.317730
C	0.991635	0.430061	3.772191
C	1.638909	0.551652	2.545791
N	1.384582	-1.640745	-0.790725
C	1.408909	-2.960767	-0.987918
C	0.244939	-3.698915	-1.281459
C	-0.984563	-3.062745	-1.373131
C	-1.048287	-1.662671	-1.179767
C	0.181435	-0.998904	-0.894885
C	0.197913	0.398307	-0.707694
N	1.330822	1.085036	-0.443334
C	0.941905	2.415625	-0.329574
C	1.740037	3.534037	-0.054229
C	1.114164	4.776614	0.031234
C	-0.279024	4.917168	-0.153690
C	-1.082574	3.813327	-0.429946
C	-0.477051	2.549891	-0.518697
C	-0.963162	1.216903	-0.772528
C	-2.163334	0.543719	-1.037727
C	-3.559937	1.069740	-1.158649
O	-3.956617	2.217358	-1.035352
N	-4.361547	-0.034274	-1.448507
C	-3.609548	-1.226815	-1.485731
O	-4.086502	-2.334348	-1.692332
C	-2.208880	-0.838652	-1.234787
C	-5.819527	0.014248	-1.544490
C	-6.487940	-0.149211	-0.192797
C	-6.810970	-1.426076	0.287006
C	-7.395947	-1.580027	1.547138
C	-7.660734	-0.457987	2.339405
C	-7.336015	0.818116	1.867287
C	-6.751433	0.971553	0.606950
H	2.302890	-2.573579	1.813327
H	1.163166	-2.955912	3.989503
H	0.298927	-0.967138	5.270833
H	0.639605	1.323007	4.278496
H	1.788111	1.520371	2.084848
H	2.378521	-3.442779	-0.913017
H	0.328466	-4.770219	-1.434252
H	-1.894517	-3.611886	-1.592809
H	-2.154101	3.909218	-0.575034
H	-0.728402	5.903951	-0.081770
H	1.714187	5.658776	0.240095
H	2.812828	3.432183	0.078584
H	-6.117710	-0.785548	-2.227302
H	-6.591474	-2.296739	-0.326124
H	-7.646196	-2.574406	1.908711
H	-8.118903	-0.577141	3.318155
H	-7.538917	1.693922	2.478711
H	-6.485796	1.960781	0.242434
H	-6.076038	0.977394	-1.993678

Figure S1. Two comparative views for the X-ray and B3LYP-D3/6-31+G(d)-LANL2DZ structures (blue and red colours, respectively) of the *N*-benzylated derivative of the Re(I) tricarbonyl complex containing pyridine and pyridocarbazole ligands (**1**[ref] in Scheme S1).

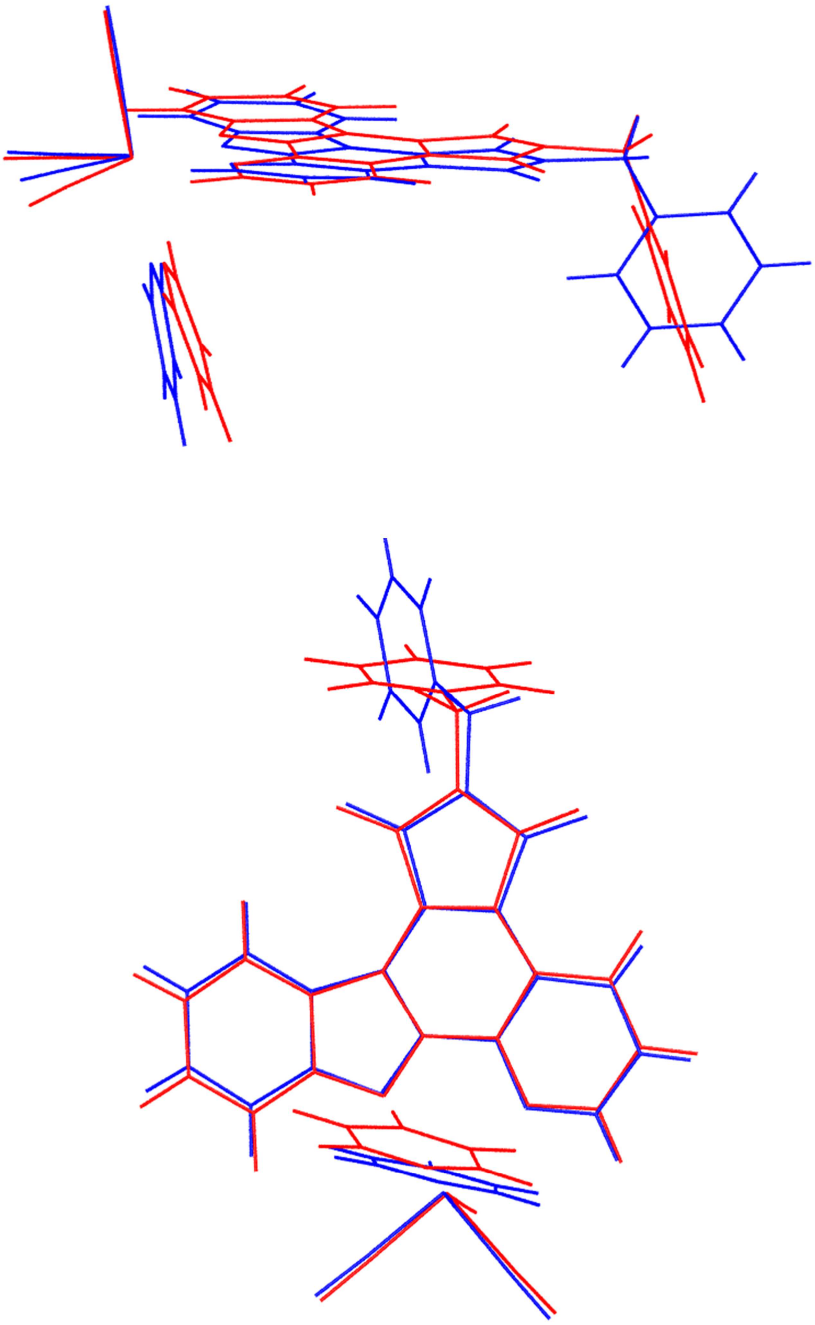

Table S2. X-Ray and B3LYP-D3/6-31+G(d)-LANL2DZ bond distances ($r_{X\text{-}Ray}$ and $r_{B3LYP\text{-}D3}$, respectively) of the Re(I) tricarbonyl complex **1**[ref] in Scheme S1. Absolute difference and square of the absolute difference of the X-Ray and B3LYP-D3/6-31+G(d)-LANL2DZ distances are also given for each bond. Only bond distances involving non-hydrogen atoms (the most relevant ones) are considered.

bond distance	$r_{X\text{-}Ray}$ (Å)	$r_{B3LYP\text{-}D3}$ (Å)	$\lvert r_{X-Ray} - r_{B3LYP-D3}\rvert$ (Å)	$\lvert r_{X-Ray} - r_{B3LYP-D3}\rvert^2$ (Å^2)
Re(1) - C(2)	1.92656	1.93083	0.00427	0.0000182
C(2) - O(3)	1.14216	1.15949	0.01733	0.0003003
Re(1) - C(4)	1.91904	1.92845	0.00941	0.0000885
C(4) - O(5)	1.15512	1.16557	0.01045	0.0001092
Re(1) - C(6)	1.91364	1.92400	0.01036	0.0001073
C(6) - O(7)	1.15496	1.16273	0.00777	0.0000604
Re(1) - N(8)	2.21128	2.25961	0.04833	0.0023358
N(8) - C(9)	1.34431	1.34898	0.00467	0.0000218
C(9) - C(10)	1.38195	1.39139	0.00944	0.0000891
C(10) - C(11)	1.38781	1.39612	0.00831	0.0000691
C(11) - C(12)	1.37191	1.39534	0.02343	0.0005490
C(12) - C(13)	1.39173	1.39205	0.00032	0.0000001
N(8) - C(13)	1.33803	1.35002	0.01199	0.0001438
Re(1) - N(14)	2.21866	2.25154	0.03288	0.0010811
Re(1) - N(21)	2.17397	2.18049	0.00652	0.0000425
N(14) - C(15)	1.32074	1.33489	0.01415	0.0002002
C(15) - C(16)	1.40748	1.40920	0.00172	0.0000030
C(16) - C(17)	1.36647	1.38737	0.02090	0.0004368
C(17) - C(18)	1.40526	1.41480	0.00954	0.0000910
C(18) - C(19)	1.41332	1.42617	0.01285	0.0001651
C(19)-N(14)	1.37225	1.36760	0.00465	0.0000216
C(19) - C(20)	1.40722	1.40979	0.00257	0.0000066
C(20) - N(21)	1.35023	1.35091	0.00068	0.0000005
N(21) - C(22)	1.38875	1.39092	0.00217	0.0000047
C(22) - C(23)	1.39289	1.40131	0.00842	0.0000709
C(23) - C(24)	1.38801	1.39392	0.00591	0.0000349
C(24) - C(25)	1.40238	1.41242	0.01004	0.0001008
C(25) - C(26)	1.37808	1.39301	0.01493	0.0002229
C(26) - C(27)	1.40214	1.40385	0.00171	0.0000029
C(27) - C(28)	1.44159	1.44140	0.00019	0.0000000
C(28) - C(20)	1.40589	1.42211	0.01622	0.0002631
C(28) - C(29)	1.41251	1.40140	0.01111	0.0001234
C(29) - C(30)	1.48885	1.49727	0.00842	0.0000709
C(30) - O(31)	1.20486	1.22049	0.01563	0.0002443
C(30) - N(32)	1.38975	1.39479	0.00504	0.0000254
N(32) - C(33)	1.40075	1.41033	0.00958	0.0000918
C(33) - O(34)	1.21731	1.22344	0.00613	0.0000376
C(33) - C(35)	1.47742	1.47497	0.00245	0.0000060
C(18) - C(35)	1.42273	1.42443	0.00170	0.0000029
C(29)-C(35)	1.38885	1.39713	0.00828	0.0000686
N(32) - C(36)	1.45464	1.46194	0.00730	0.0000533

C(36) - C(37)	1.50855	1.51676	0.00821	0.0000674
C(37) - C(38)	1.38933	1.40177	0.01244	0.0001548
C(38) - C(39)	1.38889	1.39780	0.00891	0.0000794
C(39) - C(40)	1.37658	1.39885	0.02227	0.0004960
C(40) - C(41)	1.36538	1.39885	0.03347	0.0011202
C(41) - C(42)	1.39352	1.39776	0.00424	0.0000180
C(37) - C(42)	1.38647	1.40183	0.01536	0.0002359

$$Mean\ Absolute\ Deviation\ (MAD): \left(\frac{1}{48}\right)\sum\left|r_{X-Ray} - r_{B3LYP-D3}\right| = \mathbf{0.01068}$$

$$\sum\left|r_{X-Ray} - r_{B3LYP-D3}\right|^2 = \mathbf{0.00954}$$

$$Root\ Mean\ Square\ Deviation\ (RMSD): \sqrt{\left(\frac{1}{48}\right)\sum\left|r_{X-Ray} - r_{B3LYP-D3}\right|^2} = \mathbf{0.01429}$$

Table S3. X-Ray and B3LYP-D3/6-31+G(d)-LANL2DZ bond angles in degrees ($\theta_{X\text{-}Ray}$ and $\theta_{B3LYP\text{-}D3}$, respectively) of the Re(I) tricarbonyl complex $\mathbf{1}^{\mathbf{ref}}$ in Scheme S1. Absolute difference and square of the absolute difference of the X-Ray and B3LYP-D3/6-31+G(d)-LANL2DZ angles are also given for each bond angle (in degrees and degrees squared, respectively). Only bond angles involving non-hydrogen atoms (the most relevant ones) are considered.

bond angle	θ_{X-Ray}	$\theta_{B3LYP-D3}$	$\lvert\theta_{X-Ray}-\theta_{B3LYP-D3}\rvert$	$\lvert\theta_{X-Ray}-\theta_{B3LYP-D3}\rvert^2$
Re(1) - C(2) - C(3)	178.131	178.843	0.712	0.507
Re(1) - C(4) - C(5)	178.311	177.246	1.065	1.134
Re(1) - C(6) - C(7)	177.580	178.307	0.727	0.529
Re(1) - N(8) - C(9)	120.921	121.438	0.517	0.267
Re(1) - N(8) - C(13)	121.462	120.413	1.049	1.100
Re(1) - N(14) - C(15)	129.562	129.845	0.283	0.080
Re(1) - N(14) - C(19)	112.325	112.095	0.230	0.053
Re(1) - N(21) - C(20)	111.792	111.812	0.020	0.000
Re(1) - N(21) - C(22)	142.704	142.353	0.351	0.123
C(2) - Re(1) - N(14)	96.584	93.655	2.929	8.579
C(2) - Re(1) - N(21)	95.264	93.725	1.539	2.369
C(2) - Re(1) - C(4)	88.684	91.255	2.571	6.610
C(2) - Re(1) - C(6)	88.064	91.200	3.136	9.834
C(2) - Re(1) - N(8)	178.354	174.427	3.927	15.421
C(4) - Re(1) - N(14)	95.222	94.198	1.024	1.049
C(4) - Re(1) - N(21)	171.686	169.811	1.875	3.516
C(4) - Re(1) - C(6)	90.710	91.552	0.842	0.709
C(4) - Re(1) - N(8)	91.344	92.741	1.397	1.952
C(6) - Re(1) - N(14)	172.534	172.387	0.147	0.022
C(6) - Re(1) - N(21)	96.722	97.220	0.498	0.248
C(6) - Re(1) - N(8)	93.582	92.573	1.009	1.018
N(8) - Re(1) - N(14)	81.774	82.183	0.409	0.167
N(8) - Re(1) - N(21)	84.500	81.736	2.764	7.640
N(8) - C(9) - C(10)	123.322	122.809	0.513	0.263
C(9) - C(10) - C(11)	118.326	118.933	0.607	0.368
C(10) - C(11) - C(12)	119.031	118.492	0.539	0.291
C(11) - C(12) - C(13)	119.174	119.102	0.072	0.005
C(12) - C(13) - N(8)	122.517	122.590	0.073	0.005
C(13) - N(8) - C(9)	117.617	118.073	0.456	0.208
N(14) - C(15) - C(16)	121.782	122.255	0.473	0.224
C(15) - C(16) - C(17)	120.825	120.369	0.456	0.208
C(16) - C(17) - C(18)	119.127	118.990	0.137	0.019
C(17) - C(18) - C(19)	116.781	116.682	0.099	0.010
C(17) - C(18) - C(35)	127.214	127.148	0.066	0.004
C(18) - C(19) - N(14)	123.388	123.734	0.346	0.120
C(18) - C(19) - C(20)	120.036	119.859	0.177	0.031
C(19) - N(14)- C(15)	118.087	117.962	0.125	0.016
C(19) - C(20) - C(28)	123.947	123.703	0.244	0.060
C(20) - C(28) - C(29)	115.524	115.552	0.028	0.001
C(28) - C(29) - C(35)	121.297	121.999	0.702	0.493

C(29) - C(35) - C(18)	123.153	122.709	0.444	0.197
C(35) - C(18) - C(19)	115.951	116.170	0.219	0.048
C(19) - C(20) - N(21)	122.006	122.656	0.650	0.423
C(20) - N(21) - C(22)	105.462	105.534	0.072	0.005
N(21) - C(22) - C(27)	110.520	110.764	0.244	0.060
N(21) - C(22) - C(23)	128.788	128.339	0.449	0.202
C(22) - C(23) - C(24)	118.214	117.890	0.324	0.105
C(23) - C(24) - C(25)	121.313	121.572	0.259	0.067
C(24) - C(25) - C(26)	121.358	121.071	0.287	0.082
C(25) - C(26) - C(27)	118.631	118.482	0.149	0.022
C(26) - C(27) - C(22)	119.824	120.093	0.269	0.072
C(27) - C(22) - C(23)	120.655	120.892	0.237	0.056
C(26) - C(27) - C(28)	134.490	134.265	0.225	0.051
C(27) - C(28) - C(20)	104.504	104.416	0.088	0.008
C(27) - C(28) - C(29)	139.923	140.030	0.107	0.011
C(28) - C(20) - N(21)	113.882	113.641	0.241	0.058
C(28) - C(29) - C(30)	130.634	130.192	0.442	0.195
C(29) - C(30) - O(31)	129.120	128.707	0.413	0.171
O(31) - C(30) - N(32)	125.040	125.343	0.303	0.092
C(29) - C(30) - N(32)	105.840	105.949	0.109	0.012
C(30) - N(32) - C(33)	111.887	111.613	0.274	0.075
C(30) - N(32) - C(36)	123.225	124.092	0.867	0.752
N(32) - C(33) - O(34)	124.303	124.198	0.105	0.011
N(32) - C(33) - C(35)	105.999	106.221	0.222	0.049
C(33) - C(35) - C(29)	108.194	108.380	0.186	0.035
C(33) - C(35) - C(18)	128.611	128.910	0.299	0.089
C(33) - N(32) - C(36)	124.809	123.924	0.885	0.783
O(34) - C(33) - C(35)	129.698	129.581	0.117	0.014
C(35) - C(29) - C(30)	108.065	107.808	0.257	0.066
N(32) - C(36) - C(37)	114.309	112.173	2.136	4.562
C(36) - C(37) - C(42)	122.620	120.337	2.283	5.212
C(36) - C(37) - C(38)	118.826	120.315	1.489	2.217
C(37) - C(38) - C(39)	120.256	120.354	0.098	0.010
C(38) - C(39) - C(40)	120.600	120.097	0.503	0.253
C(39) - C(40) - C(41)	119.638	119.778	0.140	0.020
C(40) - C(41) - C(42)	120.398	120.102	0.296	0.088
C(41) - C(42) - C(37)	120.575	120.349	0.226	0.051
C(42) - C(37) - C(38)	118.521	119.319	0.798	0.637

$$\textit{Mean Absolute Deviation (MAD)}: \left(\frac{1}{78}\right)\sum\left|\theta_{X-Ray} - \theta_{B3LYP-D3}\right| = \mathbf{0.6519}$$

$$\sum\left|\theta_{X-Ray} - \theta_{B3LYP-D3}\right|^2 = \mathbf{82.112}$$

$$\textit{Root Mean Square Deviation (RMSD)}: \sqrt{\left(\frac{1}{78}\right)\sum\left|\theta_{X-Ray} - \theta_{B3LYP-D3}\right|^2} = \mathbf{1.026}$$

Table S4. Excitation energies, *E*, in eV (in parentheses absorption wavelengths, *λ*, in nm) and oscillator strengths (*f*) of the first ten lowest-lying singlet-singlet electron transitions calculated at the PCM-TD-DFT/6-31+G(d)-LANL2DZ//B3LYP-D3/6-31+G(d)-LANL2DZ level for Re(I) complex **1**.

	DFT										
	B3LYP-D3	CAM-B3LYP	M05	M06	MN15	PBE	PBE0	TPSS	TPSSh	wB97x	wB97xD
E (*λ*)	2.27 (547)	2.73 (454)	2.41 (514)	2.38 (521)	2.59 (479)	1.88 (660)	2.39 (519)	1.94 (640)	2.14 (579)	2.97 (418)	2.79 (445)
f	0.1344	0.2056	0.1514	0.1459	0.1802	0.0896	0.1466	0.0960	0.1142	0.2331	0.2080
E (*λ*)	2.81 (441)	3.36 (369)	2.94 (421)	2.89 (428)	3.19 (389)	2.39 (519)	2.95 (420)	2.46 (504)	2.68 (462)	3.62 (342)	3.43 (362)
f	0.0699	0.0206	0.0573	0.0577	0.0343	0.0723	0.0629	0.0766	0.0750	0.0021	0.0116
E (*λ*)	3.08 (403)	3.67 (338)	3.24 (383)	3.18 (390)	3.45 (359)	2.56 (485)	3.21 (386)	2.66 (467)	2.93 (424)	4.04 (307)	3.74 (331)
f	0.0382	0.1676	0.0668	0.0606	0.1206	0.0025	0.0561	0.0020	0.0198	0.2763	0.1949
E (*λ*)	3.25 (381)	4.03 (308)	3.40 (365)	3.36 (369)	3.78 (328)	2.58 (480)	3.44 (360)	2.69 (462)	3.02 (411)	4.25 (292)	4.09 (303)
f	0.0116	0.0146	0.0049	0.0062	0.0056	0.0080	0.0109	0.0089	0.0090	0.0013	0.0173
E (*λ*)	3.27 (379)	4.11 (302)	3.49 (355)	3.44 (361)	3.82 (324)	2.62 (474)	3.48 (357)	2.70 (458)	3.04 (408)	4.27 (290)	4.12 (301)
f	0.0044	0.0046	0.0019	0.0058	0.0002	0.0012	0.0060	0.0031	0.0040	0.0268	0.0005
E (*λ*)	3.36 (369)	4.14 (299)	3.52 (352)	3.45 (359)	3.84 (323)	2.62 (473)	3.55 (349)	2.73 (454)	3.10 (399)	4.31 (288)	4.16 (298)
f	0.0027	0.0158	0.0069	0.0025	0.0043	0.0029	0.0036	0.0022	0.0020	0.0005	0.0029
E (*λ*)	3.40 (365)	4.15 (299)	3.54 (350)	3.49 (356)	3.88 (319)	2.71 (457)	3.58 (346)	2.82 (440)	3.16 (393)	4.44 (279)	4.22 (294)
f	0.0151	0.0073	0.0150	0.0146	0.0106	0.0294	0.0122	0.0291	0.0215	0.1233	0.0463
E (*λ*)	3.69 (336)	4.22 (294)	3.75 (330)	3.78 (328)	3.90 (318)	2.94 (422)	3.78 (328)	3.09 (401)	3.45 (359)	4.46 (278)	4.30 (288)
f	0.0455	0.0141	0.0001	0.0056	0.0012	0.0001	0.0001	0.0001	0	0.099	0.0236
E (*λ*)	3.69 (336)	4.27 (291)	3.84 (323)	3.79 (327)	3.98 (311)	3.15 (394)	3.84 (323)	3.26 (380)	3.55 (350)	4.54 (273)	4.36 (284)
f	0.0284	0.0224	0.1686	0.2133	0.0156	0.0032	0.1207	0.003	0.044	0.0595	0.1210
E (*λ*)	3.73 (332)	4.28 (290)	3.89 (319)	3.81 (325)	4.06 (305)	3.16 (393)	3.90 (318)	3.27 (379)	3.59 (345)	4.59 (270)	4.37 (284)
f	0.0368	0.2825	0.0459	0.0006	0.2623	0.0137	0.0522	0.0186	0.0387	0.1154	0.1693

Table S5. Excitation energies, *E*, in eV (in parentheses absorption wavelengths, λ, in nm) and oscillator strengths (*f*) of the first ten lowest-lying singlet-singlet electron transitions calculated at the PCM-TD-DFT/6-31+G(d)-LANL2DZ//B3LYP-D3/6-31+G(d)-LANL2DZ level for Re(I) complex **2**.

	DFT										
	B3LYP-D3	CAM-B3LYP	M05	M06	MN15	PBE	PBE0	TPSS	TPSSh	wB97x	wB97xD
E (λ)	2.69 (462)	3.25 (381)	2.84 (436)	2.78 (446)	3.06 (405)	2.23 (556)	2.82 (440)	2.31 (537)	2.54 (488)	3.56 (348)	3.32 (374)
f	0.0638	0.1007	0.0712	0.0676	0.0877	0.0371	0.0706	0.0439	0.0557	0.1240	0.1053
E (λ)	2.97 (417)	3.67 (338)	3.24 (382)	3.16 (392)	3.51 (353)	2.30 (539)	3.19 (389)	2.40 (517)	2.74 (452)	3.87 (321)	3.73 (333)
f	0.0061	0.0009	0.0067	0.0066	0.0021	0.0143	0.0067	0.0101	0.0058	0.0009	0.0010
E (λ)	3.25 (382)	3.95 (314)	3.36 (369)	3.29 (376)	3.63 (341)	2.84 (437)	3.37 (368)	2.91 (426)	3.13 (396)	4.19 (296)	4.06 (306)
f	0.0095	0.0248	0.0088	0.0122	0.0155	0.0115	0.0071	0.0148	0.0121	0.0006	0.0043
E (λ)	3.50 (354)	4.05 (306)	3.67 (338)	3.57 (347)	3.81 (325)	2.89 (430)	3.66 (339)	3.01 (412)	3.34 (371)	4.28 (289)	4.13 (301)
f	0.0307	0.1339	0.1321	0.0443	0.0352	0.0044	0.1175	0.0035	0.0085	0.0884	0.2089
E (λ)	3.55 (350)	4.11 (302)	3.73 (333)	3.63 (341)	3.90 (318)	2.92 (425)	3.72 (333)	3.02 (410)	3.38 (367)	4.44 (280)	4.20 (295)
f	0.1018	0.0640	0.0487	0.1115	0.1473	0.0022	0.0493	0.0021	0.0320	0.4352	0.0123
E (λ)	3.65 (340)	4.30 (288)	3.90 (318)	3.79 (327)	4.11 (302)	3.05 (407)	3.88 (320)	3.14 (394)	3.43 (361)	4.52 (274)	4.38 (283)
f	0.0115	0.2694	0.0071	0.0122	0.1596	0.0121	0.0020	0.0109	0.0682	0.0175	0.3124
E (λ)	3.73 (333)	4.40 (282)	3.96 (313)	3.85 (322)	4.16 (298)	3.10 (400)	3.95 (314)	3.20 (388)	3.51 (353)	4.62 (268)	4.43 (280)
f	0.0088	0.0086	0.0753	0.0832	0.0054	0.0809	0.0095	0.0894	0.0221	0.0665	0.0087
E (λ)	3.76 (330)	4.47 (277)	3.98 (311)	3.90 (318)	4.26 (291)	3.19 (389)	3.97 (313)	3.28 (379)	3.59 (345)	4.67 (266)	4.52 (274)
f	0.0709	0.0855	0.0257	0.0078	0.0223	0.0153	0.0853	0.0216	0.0373	0.1414	0.0078
E (λ)	3.90 (318)	4.49 (276)	4.01 (309)	3.92 (316)	4.33 (287)	3.28 (378)	4.05 (306)	3.41 (363)	3.71 (334)	4.69 (265)	4.54 (273)
f	0.0224	0.1854	0.0129	0.0103	0.2907	0.0062	0.0237	0.0115	0.0249	0.1638	0.2382
E (λ)	3.92 (316)	4.61 (269)	4.02 (308)	3.95 (314)	4.38 (283)	3.31 (374)	4.08 (304)	3.43 (361)	3.74 (332)	4.75 (261)	4.64 (267)
f	0.0075	0.0849	0.0199	0.0220	0.0060	0.0349	0.0056	0.0295	0.0154	0.0800	0.0955

Table S6. Excitation energies, *E*, in eV (in parentheses absorption wavelengths, *λ*, in nm) and oscillator strengths (*f*) of the first ten lowest-lying singlet-singlet electron transitions calculated at the PCM-TD-DFT/6-31+G(d)-LANL2DZ//B3LYP-D3/6-31+G(d)-LANL2DZ level for Re(I) complex **3**.

	DFT										
	B3LYP-D3	CAM-B3LYP	M05	M06	MN15	PBE	PBE0	TPSS	TPSSh	wB97x	wB97xD
E (*λ*)	2.94 (422)	3.53 (351)	3.11 (399)	3.05 (407)	3.33 (372)	2.32 (533)	3.08 (403)	2.44 (509)	2.77 (448)	3.79 (327)	3.59 (346)
f	0.0944	0.3091	0.1413	0.1335	0.1911	0.0049	0.1128	0.0059	0.0676	0.5748	0.3361
E (*λ*)	3.06 (405)	3.68 (337)	3.34 (372)	3.26 (381)	3.53 (351)	2.42 (512)	3.28 (378)	2.51 (494)	2.81 (441)	3.92 (316)	3.74 (332)
f	0.0046	0.3310	0.0072	0.0060	0.3852	0.0438	0.0051	0.0496	0.0074	0.1262	0.3172
E (*λ*)	3.30 (376)	4.05 (306)	3.39 (365)	3.33 (373)	3.72 (333)	2.74 (453)	3.41 (364)	2.84 (436)	3.19 (389)	4.24 (293)	4.13 (300)
f	0.3312	0.0029	0.3704	0.3587	0.0056	0.0002	0.3714	0.0008	0.1273	0.0012	0.0019
E (*λ*)	3.45 (359)	4.17 (298)	3.71 (334)	3.62 (343)	3.89 (319)	2.86 (433)	3.68 (337)	2.98 (416)	3.23 (383)	4.39 (282)	4.29 (289)
f	0.0198	0.0088	0.0108	0.0089	0.0045	0.0083	0.0137	0.0256	0.1486	0.0357	0.0219
E (*λ*)	3.53 (351)	4.27 (290)	3.76 (330)	3.67 (338)	4.06 (305)	2.94 (422)	3.72 (333)	3.04 (408)	3.33 (373)	4.51 (275)	4.32 (287)
f	0.0173	0.0220	0.0035	0.0016	0.0026	0.0206	0.0084	0.2049	0.0378	0.0032	0.0224
E (*λ*)	3.62 (343)	4.39 (282)	3.82 (325)	3.70 (335)	4.14 (299)	2.96 (419)	3.81 (326)	3.07 (403)	3.43 (361)	4.62 (268)	4.42 (280)
f	0.0049	0.0086	0.0083	0.0129	0.0082	0.1897	0.0071	0.0085	0.0025	0.0222	0.0097
E (*λ*)	3.82 (325)	4.40 (282)	4.00 (310)	3.92 (316)	4.17 (297)	3.14 (395)	4.04 (307)	3.23 (384)	3.59 (346)	4.69 (265)	4.51 (275)
f	0.0109	0.0551	0.0248	0.0361	0.0542	0.0114	0.0019	0.0129	0.0122	0.0148	0.0229
E (*λ*)	3.86 (321)	4.47 (277)	4.03 (308)	3.94 (315)	4.19 (296)	3.26 (380)	4.05 (306)	3.36 (369)	3.69 (336)	4.76 (261)	4.52 (274)
f	0.0133	0.0361	0.0442	0.0203	0.0375	0.002	0.0175	0.0032	0.0026	0.0293	0.0588
E (*λ*)	3.89 (319)	4.53 (274)	4.05 (306)	3.97 (312)	4.27 (291)	3.32 (374)	4.06 (305)	3.44 (361)	3.72 (333)	4.83 (257)	4.64 (267)
f	0.0223	0.0220	0.0204	0.0048	0.0490	0.0188	0.0466	0.0241	0.0262	0.0545	0.0328
E (*λ*)	3.97 (312)	4.64 (267)	4.10 (303)	3.99 (311)	4.45 (279)	3.36 (369)	4.12 (301)	3.49 (355)	3.80 (326)	5.04 (246)	4.72 (263)
f	0.0078	0.0206	0.0110	0.0076	0.0395	0.0075	0.0086	0.0066	0.0017	0.0805	0.0206

Figure S2. Simulated electronic spectra obtained at the PCM-TD-DFT/6-31+G(d)-LANL2DZ//B3LYP-D3/6-31+G(d)-LANL2DZ (DFT = B3LYP-D3, CAM-B3LYP, M05, M06, MN15, PBE, PBE0, TPSS, TPSSh, wB97x, wB97xD) levels for complex **1**. Experimental UV/Vis absorbance spectrum measured in DMSO for the same complex is also provided for comparison purposes.

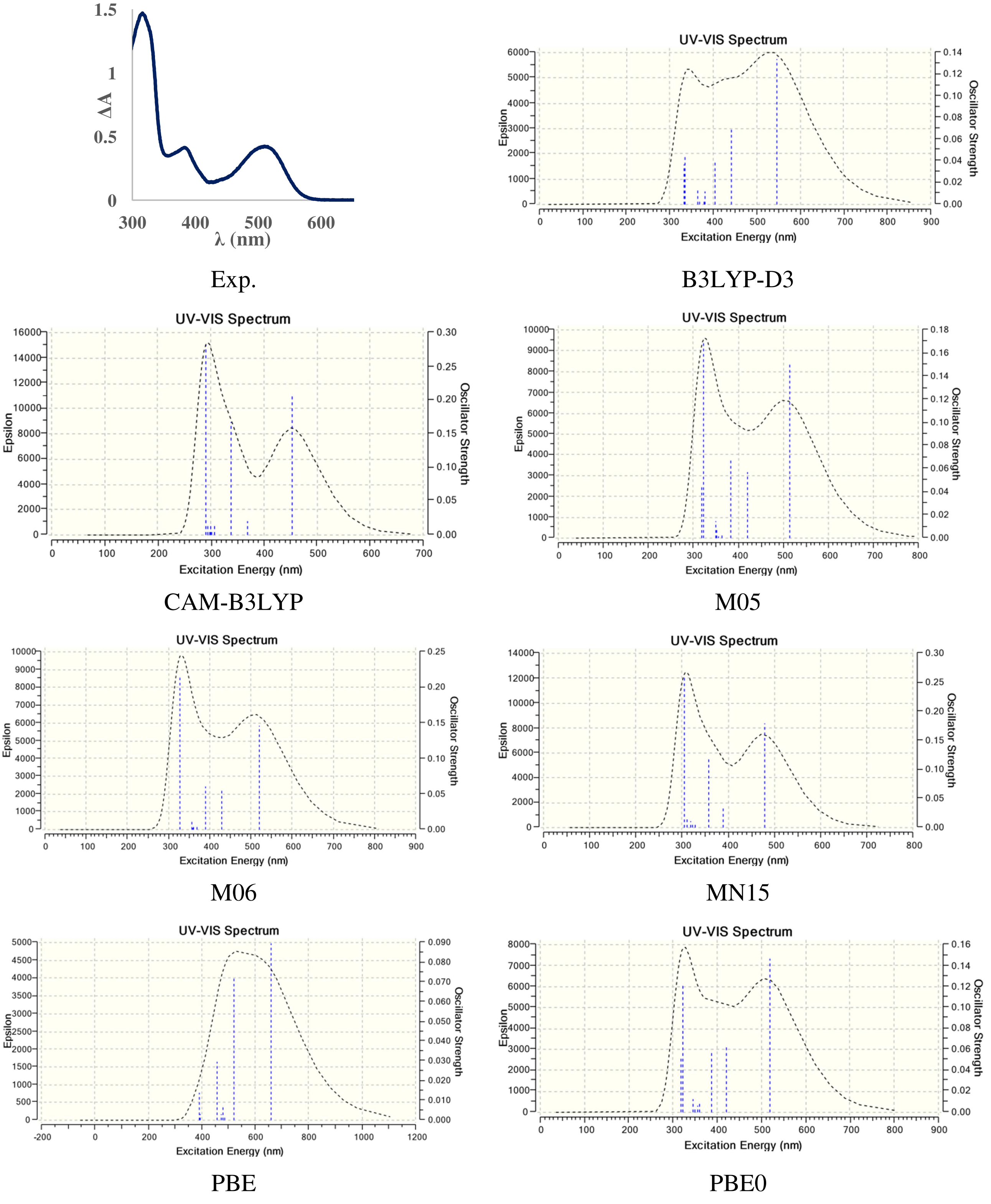

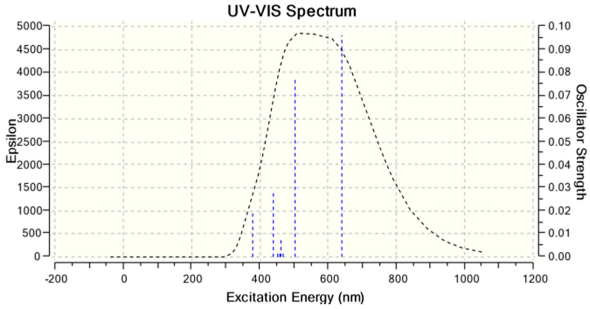

TPSS

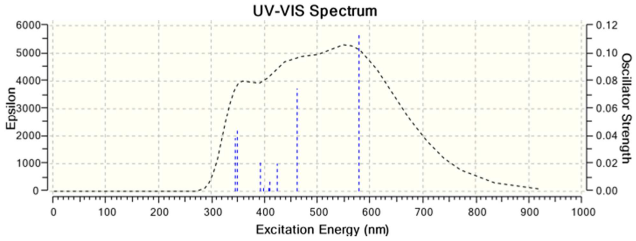

TPSSh

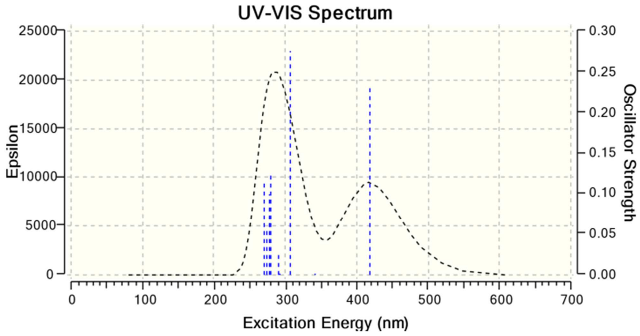

wB97x

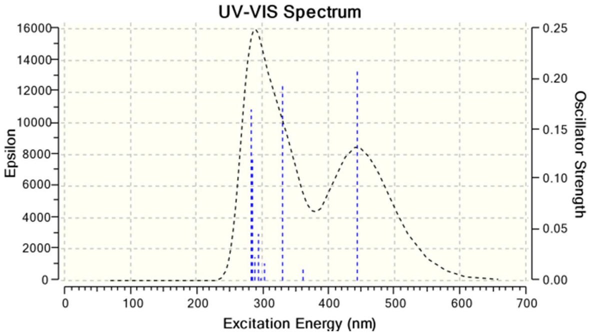

wB97xD

Figure S3. Simulated electronic spectra obtained at the PCM-TD-DFT/6-31+G(d)-LANL2DZ//B3LYP-D3/6-31+G(d)-LANL2DZ (DFT = B3LYP-D3, CAM-B3LYP, M05, M06, MN15, PBE, PBE0, TPSS, TPSSh, wB97x, wB97xD) levels for complex **2**. Experimental UV/Vis absorbance spectrum measured in DMSO for the same complex is also provided for comparison purposes.

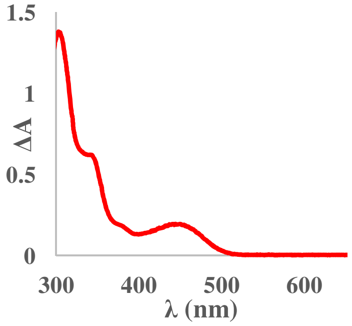

Exp.

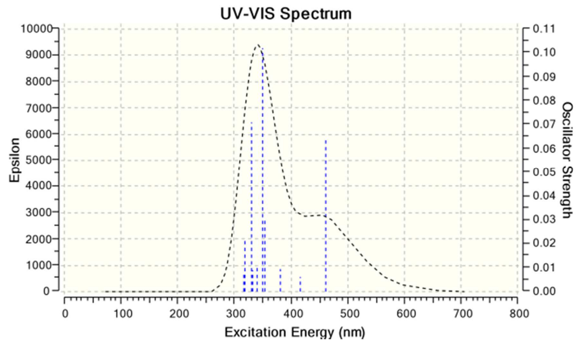

B3LYP-D3

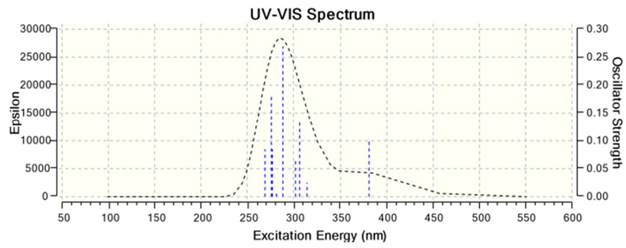

CAM-B3LYP

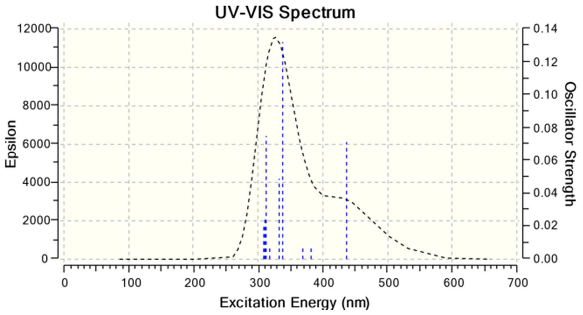

M05

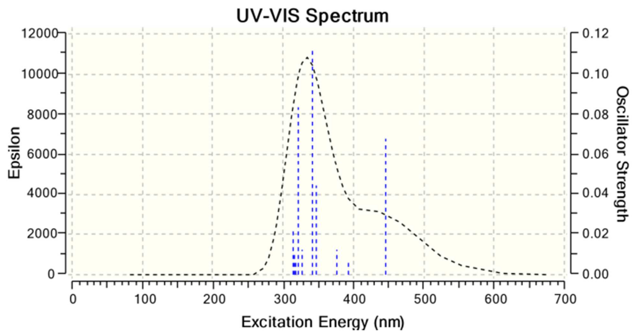

M06

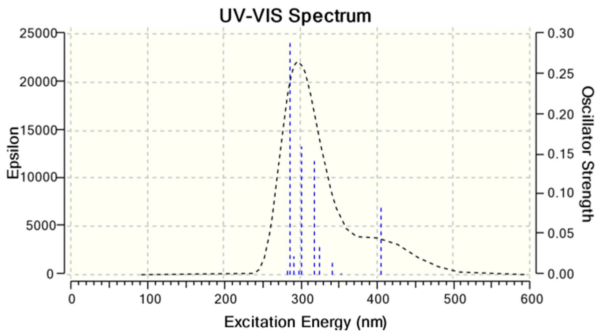

MN15

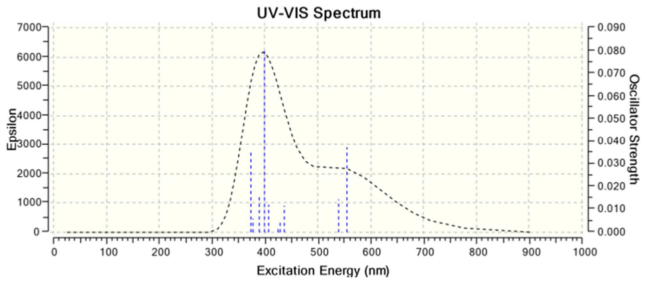

PBE

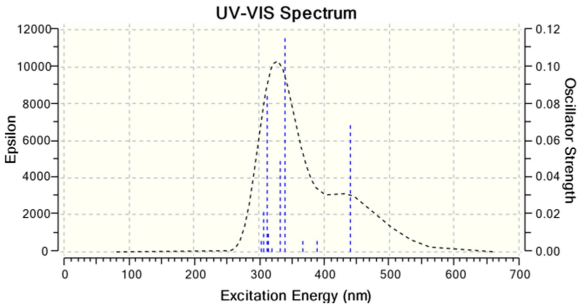

PBE0

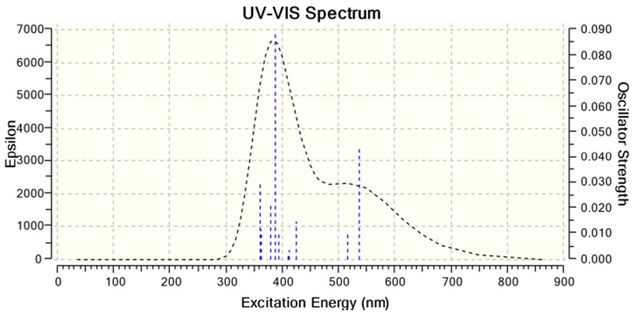

TPSS

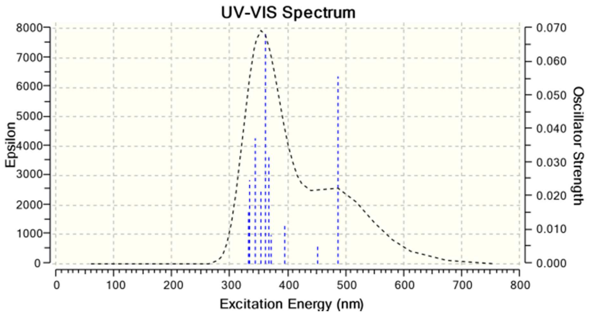

TPSSh

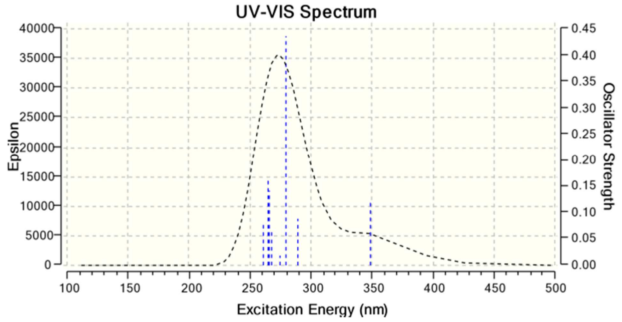

wB97x

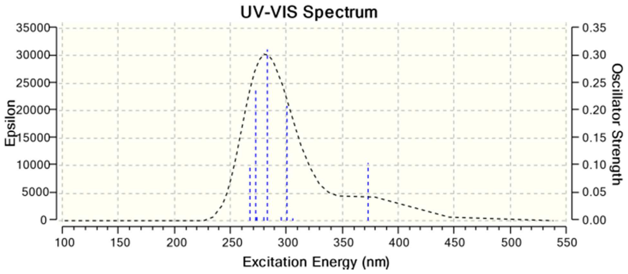

wB97xD

Figure S4. Simulated electronic spectra obtained at the PCM-TD-DFT/6-31+G(d)-LANL2DZ//B3LYP-D3/6-31+G(d)-LANL2DZ (DFT = B3LYP-D3, CAM-B3LYP, M05, M06, MN15, PBE, PBE0, TPSS, TPSSh, wB97x, wB97xD) levels for complex **3**. Experimental UV/Vis absorbance spectrum measured in DMSO for the same complex is also provided for comparison purposes.

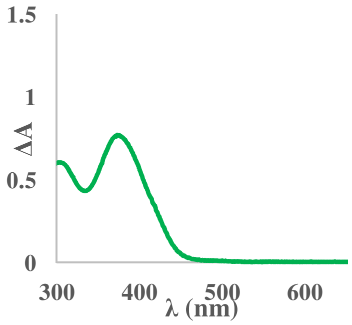

Exp.

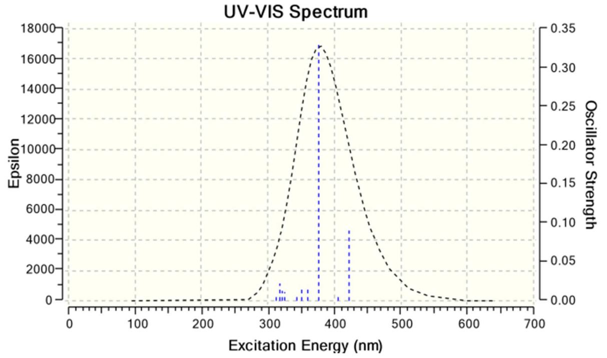

B3LYP-D3

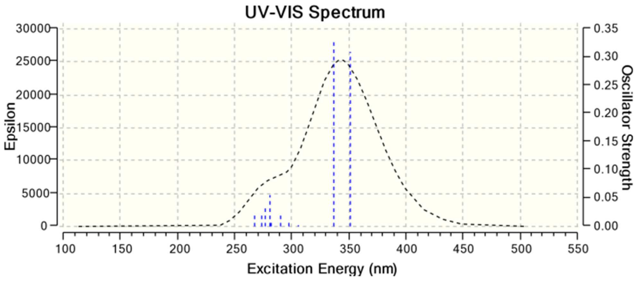

CAM-B3LYP

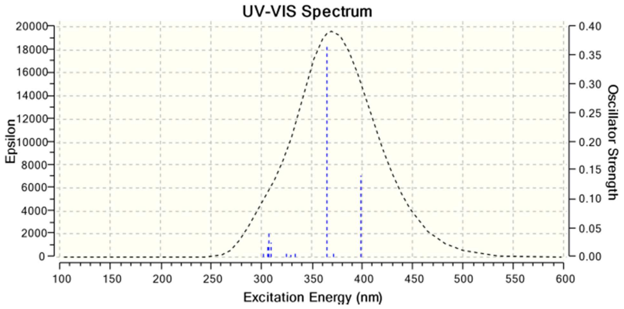

M05

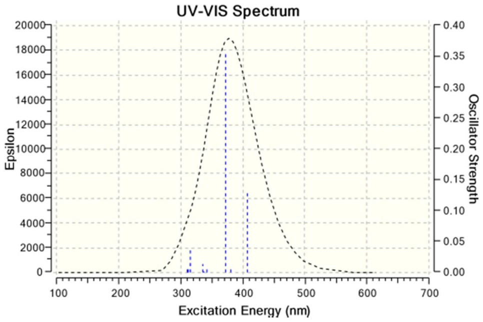

M06

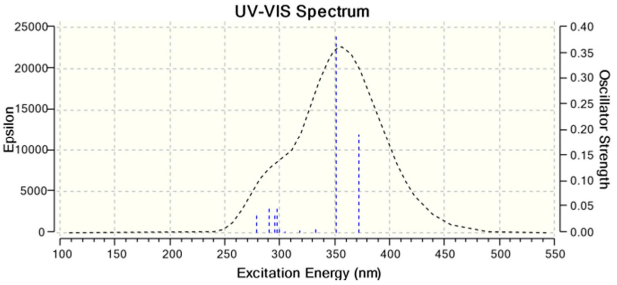

MN15

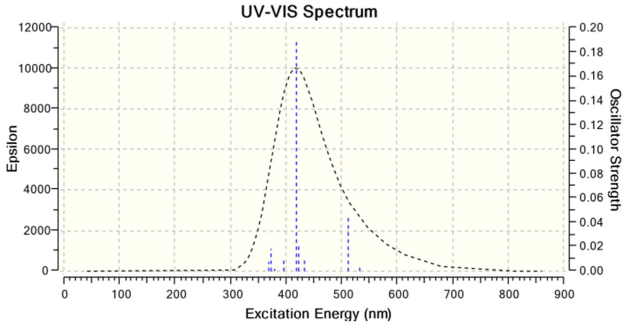

PBE

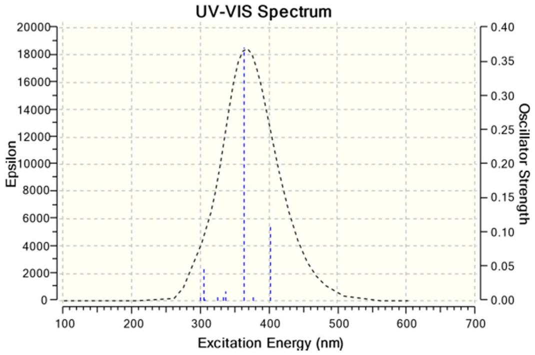

PBE0

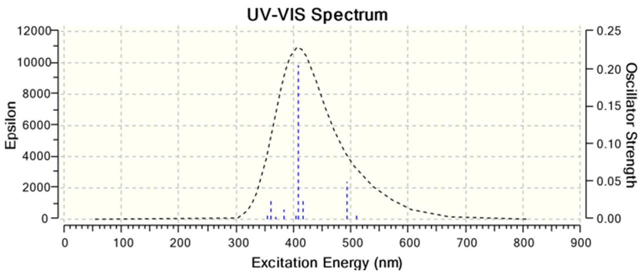

TPSS

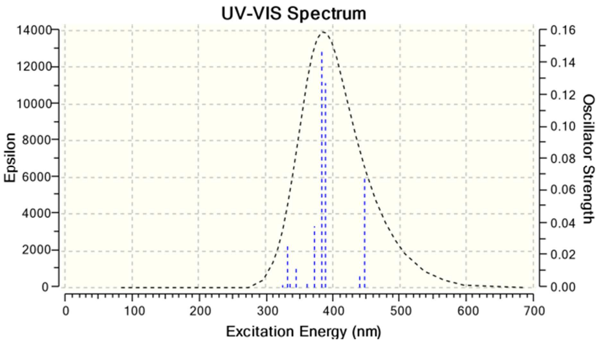

TPSSh

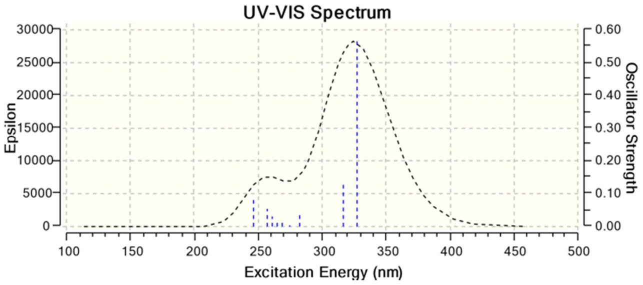

wB97x

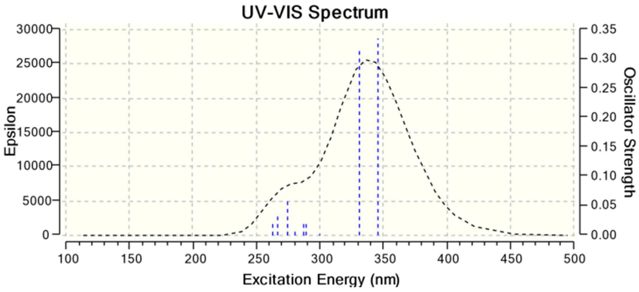

wB97xD

Table S7. Excitation energy, *E* (in parenthesis the corresponding absorption wavelength, λ), of the most red-shifted absorption band found for complexes **1-3** at the PCM-TD-DFT/6-31+G(d)-LANL2DZ//B3LYP-D3/6-31+G(d)-LANL2DZ levels along with the difference in absolute value between the computed excitation energies and the respective experimental value, ΔE, for each level of theory and complex and the total error, ΔE_{total}, obtained for each level of theory, ΔE_{total}.

	E/eV (λ/nm)			*ΔE/eV*			$\Delta E_{total}/eV$
DFT	**1**	**2**	**3**	**1**	**2**	**3**	
B3LYP-D3	2.27 (547)	2.69 (462)	3.30 (376)	0.15	0.09	0.02	0.26
CAM-B3LYP	2.73 (454)	3.25 (381)	3.53 (351)	0.31	0.47	0.21	0.99
M05	2.41 (514)	2.84 (436)	3.39 (365)	0.01	0.06	0.07	0.14
M06	2.38 (521)	2.78 (446)	3.33 (373)	0.04	0.00	0.00	0.04
MN15	2.59 (479)	3.06 (405)	3.33 (372)	0.17	0.28	0.01	0.46
PBE	2.39 (519)	3.10 (400)	2.96 (419)	0.03	0.32	0.36	0.71
PBE0	2.39 (519)	2.82 (440)	3.41 (364)	0.03	0.04	0.09	0.16
TPSS	2.46 (504)	3.20 (388)	3.04 (408)	0.04	0.42	0.28	0.74
TPSSh	2.68 (462)	2.54 (488)	3.23 (383)	0.26	0.24	0.09	0.59
wB97x	2.97 (418)	3.56 (348)	3.79 (327)	0.55	0.78	0.47	1.80
wB97xD	2.79 (445)	3.32 (374)	3.59 (346)	0.37	0.54	0.27	1.18
exp.	2.42 (512)	2.78 (446)	3.32 (373)				

Table S8. B3LYP-D3/6-31+G(d)-LANL2DZ relevant optimized geometry data for Re(I) complexes **1-3** and **1a-1s** in their singlet ground states. Bond distances, bond angles, and dihedral angles are given in Å, degrees, and degrees, respectively. The atom numbering used is collected for complex **1**.[a]

	Re-C1	Re-C2	Re-L3	Re-N4	Re-N5	Re-N6	N4-Re-N7	L3-Re-N7	C8-N4-Re-N7	C9-N5-N6-C10
**1**	1.928	1.924	1.931	2.259	2.251	2.181	75.7	98.3	-80.4	1.3
**2**	1.928	1.923	1.929	2.261	2.254	2.178				1.1
**3**	1.929	1.927	1.928	2.265	2.224	2.158				2.1
**1a**	1.931	1.923	1.931	2.258	2.254	2.183	76.5	98.1	-79.1	0.7
**1b**	1.929	1.924	1.932	2.258	2.254	2.181	76.0	98.2	-79.4	1.0
**1c**	1.929	1.924	1.931	2.260	2.251	2.178	75.4	98.7	-79.3	1.3
**1d**	1.929	1.924	1.931	2.260	2.251	2.177	75.4	98.6	-79.8	1.2
**1e**	1.931	1.922	1.931	2.259	2.254	2.180	76.2	98.3	-78.4	0.5
**1f**	1.931	1.926	1.931	2.258	2.254	2.179	76.2	98.3	-78.5	0.6
**1g**	1.930	1.923	1.932	2.257	2.254	2.185	77.0	97.6	-80.1	1.0
**1h**	1.930	1.923	1.932	2.259	2.254	2.178	75.8	98.8	-78.6	0.7
**1i**	1.902	1.900	2.381	2.225	2.258	2.194	81.6	90.1	-76.8	-2.0
**1j**	1.901	1.900	2.382	2.226	2.260	2.199	82.7	88.3	-77.0	-1.9
**1k**	1.906	1.903	2.359	2.217	2.263	2.197	87.9	82.0	-76.0	-5.9
**1l**	1.905	1.899	2.380	2.224	2.259	2.196	81.9	89.9	-76.7	-2.1
**1m**	1.905	1.899	2.380	2.224	2.259	2.193	81.6	90.1	-76.0	-1.9
**1n**	1.904	1.899	2.383	2.225	2.261	2.200	83.1	88.0	-76.7	-1.9
**1o**	1.904	1.898	2.382	2.226	2.261	2.198	82.5	88.5	-76.0	-2.0
**1p**	1.903	1.900	2.382	2.226	2.257	2.194	81.6	90.3	-76.2	-2.0
**1q**	1.904	1.900	2.382	2.226	2.258	2.191	81.5	90.2	-75.5	-2.3
**1r**	1.902	1.900	2.385	2.227	2.259	2.199	82.3	88.7	-76.4	-1.8
**1s**	1.902	1.899	2.384	2.227	2.260	2.196	82.7	88.3	-75.3	-2.0

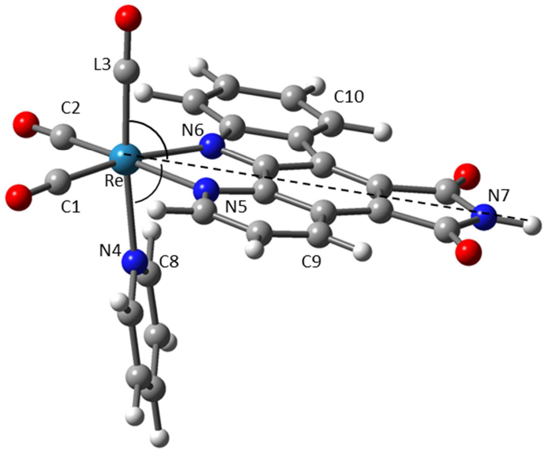

[a] L3 is the carbon atom of the carbonyl ligand in *trans* disposition to the pyridine ligand for complexes **1-3** and **1a-1h** or the phosphorus atom of the phosphine ligand in *trans* disposition to the pyridine ligand for complexes **1i-1s**.

Table S9. Excitation energies in eV (*E*) and nm (λ), and oscillator strengths (*f*) of the first ten lowest-lying singlet-singlet electron transitions calculated at the PCM-TD-M06/6-31+G(d)-LANL2DZ//B3LYP-D3/6-31+G(d)-LANL2DZ level for Re(I) complexes **1**-**3**.

Transition		Complex		
		**1**	**2**	**3**
1	*E (λ)*	**2.38 (521)**	**2.78 (446)**	3.05 (407)
	f	**0.1459**	**0.0676**	0.1335
2	*E (λ)*	2.89 (428)	3.16 (392)	3.26 (381)
	f	0.0577	0.0066	0.0060
3	*E (λ)*	**3.18 (390)**	3.29 (376)	**3.33 (373)**
	f	**0.0606**	0.0122	**0.3587**
4	*E (λ)*	3.36 (369)	3.57 (347)	3.62 (343)
	f	0.0062	0.0443	0.0089
5	*E (λ)*	3.44 (361)	**3.63 (341)**	3.67 (338)
	f	0.0058	**0.1115**	0.0016
6	*E (λ)*	3.45 (359)	3.79 (327)	3.70 (335)
	f	0.0025	0.0122	0.0129
7	*E (λ)*	3.49 (356)	3.85 (322)	3.92 (316)
	f	0.0146	0.0832	0.0361
6	*E (λ)*	3.78 (328)	3.90 (318)	3.94 (315)
	f	0.0056	0.0078	0.0203
9	*E (λ)*	**3.79 (327)**	3.92 (316)	3.97 (312)
	f	**0.2133**	0.0103	0.0048
10	*E (λ)*	3.81 (325)	3.95 (314)	3.99 (311)
	f	0.0006	0.0220	0.0076

Table S10. B3LYP-D3/6-31+G(d)-LANL2DZ relevant optimized geometry data for Re(I) complexes **1**-**3** and **1a**-**1s** in their triplet excited states. Bond distances, bond angles, and dihedral angles are given in Å, degrees, and degrees, respectively. The atom numbering used is collected for complex **1**.[a]

	Re-C1	Re-C2	Re-L3[a]	Re-N4	Re-N5	Re-N6	N4-Re-N7	L3-Re-N7	C8-N4-Re-N7	C9-N5-N6-C10
**1**	1.936	1.926	1.935	2.164	2.236	2.260	74.3	100.7	-84.9	0.9
**2**	1.933	1.928	1.930	2.174	2.209	2.268				1.0
**3**	1.934	1.930	1.929	2.153	2.192	2.270				-1.3
**1a**	1.940	1.925	1.936	2.161	2.237	2.259	75.4	99.7	-82.6	-0.1
**1b**	1.937	1.926	1.936	2.162	2.237	2.260	74.6	100.4	-84.1	0.5
**1c**	1.936	1.925	1.934	2.164	2.234	2.261	74.0	100.8	-85.9	0.7
**1d**	1.934	1.924	1.932	2.168	2.235	2.263	74.0	100.5	-85.4	0.2
**1e**	1.939	1.924	1.935	2.162	2.235	2.261	75.2	99.7	-83.0	-0.4
**1f**	1.937	1.923	1.932	2.168	2.237	2.262	75.3	99.4	-83.2	-0.8
**1g**	1.940	1.926	1.936	2.162	2.238	2.258	75.8	99.4	-83.6	0.3
**1h**	1.937	1.924	1.935	2.163	2.235	2.261	74.5	100.4	-84.7	-0.03
**1i**	1.927	1.902	2.396	2.129	2.248	2.228	81.4	91.0	-83.0	-0.4
**1j**	1.931	1.903	2.399	2.120	2.250	2.229	82.4	89.8	-82.8	-0.5
**1k**	1.923	1.905	2.369	2.221	2.250	2.147	85.1	85.7	-82.2	-3.5
**1l**	1.935	1.902	2.401	2.118	2.246	2.227	82.3	90.5	-81.9	-0.6
**1m**	1.929	1.900	2.395	2.129	2.247	2.228	82.0	90.4	-81.7	-0.7
**1n**	1.938	1.902	2.401	2.133	2.246	2.228	84.1	88.4	-82.0	0.5
**1o**	1.932	1.900	2.397	2.124	2.248	2.229	83.5	88.4	-81.9	0.2
**1p**	1.931	1.902	2.403	2.122	2.248	2.227	81.1	90.8	-82.8	-0.1
**1q**	1.925	1.900	2.395	2.132	2.249	2.229	81.4	90.8	-82.8	-0.7
**1r**	1.936	1.903	2.401	2.114	2.249	2.229	82.4	89.8	-82.8	-0.4
**1s**	1.928	1.900	2.398	2.128	2.249	2.230	82.8	88.9	-83.3	0.1

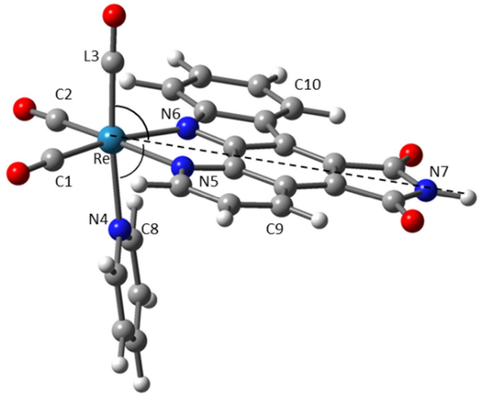

[a] L3 is the carbon atom of the carbonyl ligand in *trans* disposition to the pyridine ligand for complexes **1**-**3** and **1a**-**1h** or the phosphorus atom of the phosphine ligand in *trans* disposition to the pyridine ligand for complexes **1i**-**1s**.

Table S11. Energies in the singlet ground state and triplet excited state (E_{S_0} and E_{T_1} in hartree, respectively), difference between E_{T_1} and E_{S_0} (ΔE_{ST}, in kcal/mol), excitation energy in nm and eV (λ_{max} (*E*)), oscillator strength (*f*), dominant orbital excitations with their corresponding coefficients for the lowest-lying absorption band, HOMO and LUMO energies (E_H and E_L, in eV, respectively), and difference between E_L and E_H ($\Delta E_{H\rightarrow L}$, in eV) of the Re(I) complexes **1-3** and **1a-1s** calculated at the PCM-M06/6-31+G(d)-LANL2DZ//B3LYP-D3/6-31+G(d)-LANL2DZ level.

Species	E_{S_0}	E_{T_1}	ΔE_{ST}	λ_{max} (*E*)	*f*	Configuration	E_H	E_L	$\Delta E_{H\rightarrow L}$
**1**	-1633.912888	-1633.851279	38.7	521 (2.38)	0.1459	H → L (0.6960)	-5.98	-2.82	3.16
**2**	-1353.183798	-1353.108711	47.1	446 (2.78)	0.0676	H → L (0.6993)	-5.64	-1.98	3.66
**3**	-1277.008324	-1276.930089	49.1	373 (3.33)	0.3587	H-1 → L (0.6718)/H → L (-0.1659)	-5.73	-1.88	3.85
**1a**	-1900.959515	-1900.899520	37.7	534 (2.32)	0.1386	H → L (0.6937)	-6.05	-2.96	3.09
**1b**	-1733.125226	-1733.064473	38.1	532 (2.33)	0.1435	H → L (0.6967)	-6.04	-2.93	3.11
**1c**	-1748.373801	-1748.317551	35.3	565 (2.19)	0.1292	H → L (0.6987)	-5.75	-2.80	2.95
**1d**	-1767.776972	-1767.732364	28.0	659 (1.88)	0.1007	H → L (0.6998)	-5.35	-2.77	2.58
**1e**	-2015.420137	-2015.365833	34.1	579 (2.14)	0.1415	H → L (0.6942)	-5.81	-2.94	2.87
**1f**	-2034.824141	-2034.781497	26.8	678 (1.83)	0.1312	H → L (0.6935)/H → L+1 (-0.1110)	-5.40	-2.94	2.46
**1g**	-2168.011959	-2167.948813	39.6	517 (2.40)	0.1537	H → L (0.6945)	-6.21	-3.04	3.17
**1h**	-1847.585859	-1847.531178	34.3	578 (2.14)	0.1292	H → L (0.6994)	-5.80	-2.91	2.89
**1i**	-1981.602431	-1981.546457	35.1	584 (2.12)	0.0979	H-1 → L (0.1119)/H → L (0.6897)	-5.61	-2.74	2.87
**1j**	-2379.582469	-2379.527312	34.6	611 (2.03)	0.0767	H-1 → L (-0.3159)/H → L (0.6171)	-5.44	-2.75	2.69
**1k**	-2528.857023	-2528.798015	37.0	559 (2.22)	0.1017	H-1 → L (0.1114)/H → L (0.6898)	-5.77	-2.80	2.97
**1l**	-2248.648310	-2248.595150	33.4	608 (2.04)	0.0945	H-1 → L (0.1233)/H → L (0.6875)	-5.66	-2.88	2.78
**1m**	-2363.109107	-2363.059803	30.9	631 (1.96)	0.1161	H → L (0.6924)	-5.54	-2.86	2.68
**1n**	-2646.628760	-2646.577198	32.4	644 (1.92)	0.0698	H-1 → L (-0.3458)/H → L (0.6007)	-5.48	-2.89	2.59
**1o**	-2761.089141	-2761.040814	30.3	653 (1.90)	0.0934	H-1 → L (-0.2543)/H → L (0.6431)	-5.45	-2.87	2.58
**1p**	-2080.815019	-2080.760020	34.5	601 (2.06)	0.0860	H-1 → L (0.1407)/H → L (0.6839)	-5.67	-2.84	2.83
**1q**	-2195.275858	-2195.225650	31.5	627 (1.98)	0.0995	H-1 → L (-0.1015)/H → L (0.6926)	-5.54	-2.81	2.63
**1r**	-2478.794985	-2478.741640	33.5	632 (1.96)	0.0651	H-2 → L (0.1021)/H-1 → L (-0.4084)/H → L (0.5515)	-5.48	-2.85	2.63
**1s**	-2593.255739	-2593.206056	31.2	645 (1.93)	0.0822	H-2 → L (0.1029)/H-1 → L (-0.2869)/H → L (0.6261)	-5.45	-2.82	2.63

Table S12. Variations of some relevant bond distances (in Å), bond angles (in degrees), and dihedral angles (in degrees) when going from their singlet ground states to their corresponding triplet excited states of Re(I) complexes **1**-**3** and **1a**-**1s** at the B3LYP-D3/6-31+G(d)-LANL2DZ level of theory. The atom numbering used is collected for complex **1**.[a]

	Re-C1	Re-C2	Re-L3[a]	Re-N4	Re-N5	Re-N6	N4-Re-N7	C3-Re-N7	C8-N4-Re-N7	C9-N5-N6-C10
**1**	0.008	0.002	0.004	-0.017	-0.015	0.001	-1.4	2.4	-4.5	-0.4
**2**	0.005	0.005	0.001	-0.004	-0.045	0.007				-0.1
**3**	0.005	0.003	0.001	-0.005	-0.032	0.005				-3.4
**1a**	0.009	0.002	0.005	-0.022	-0.017	0.001	-1.1	1.6	-3.5	-0.8
**1b**	0.008	0.002	0.004	-0.019	-0.017	0.002	-1.4	2.2	-4.7	-0.5
**1c**	0.007	0.001	0.003	-0.014	-0.017	0.001	-1.4	2.1	-6.6	-0.6
**1d**	0.005	0.000	0.001	-0.009	-0.016	0.003	-1.4	1.9	-5.6	-1.0
**1e**	0.008	0.002	0.004	-0.018	-0.019	0.002	-1.0	1.4	-4.6	-0.9
**1f**	0.006	-0.003	0.001	-0.011	-0.017	0.004	-0.9	1.1	-4.7	-1.4
**1g**	0.010	0.003	0.004	-0.023	-0.016	0.001	-1.2	1.8	-3.5	-0.7
**1h**	0.007	0.001	0.003	-0.015	-0.019	0.002	-1.3	1.6	-6.1	-0.7
**1i**	0.025	0.002	0.015	-0.065	-0.010	0.003	-0.2	0.9	-6.2	1.6
**1j**	0.030	0.003	0.017	-0.079	-0.010	0.003	-0.3	1.5	-5.8	1.4
**1k**	0.017	0.002	0.010	-0.050	-0.013	0.004	-2.8	3.7	-6.2	-2.4
**1l**	0.030	0.003	0.021	-0.078	-0.013	0.003	0.4	0.6	-5.2	1.5
**1m**	0.024	0.001	0.015	-0.064	-0.012	0.004	0.4	0.3	-5.7	1.2
**1n**	0.034	0.003	0.018	-0.067	-0.015	0.003	1.0	0.4	-5.3	2.4
**1o**	0.028	0.002	0.015	-0.074	-0.013	0.003	1.0	-0.1	-5.9	2.2
**1p**	0.028	0.002	0.021	-0.072	-0.009	0.001	-0.5	0.5	-6.6	1.9
**1q**	0.021	0.000	0.013	-0.059	-0.009	0.003	-0.1	0.6	-7.3	1.6
**1r**	0.034	0.003	0.016	-0.085	-0.010	0.002	0.1	1.1	-6.4	1.4
**1s**	0.026	0.001	0.014	-0.068	-0.011	0.003	0.1	0.6	-8.0	2.1

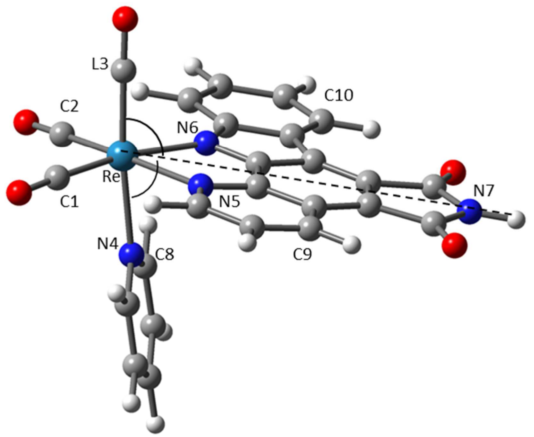

[a] L3 is the carbon atom of the carbonyl ligand in *trans* disposition to the pyridine ligand for complexes **1**-**3** and **1a**-**1h** or the phosphorus atom of the phosphine ligand in *trans* disposition to the pyridine ligand for complexes **1i**-**1s**.

Figure S5. B3LYP-D3/6-31+G(d)-LANL2DZ optimized structures in the singlet ground state of Re(I) complexes **1a**-**1s**. For clarity, two views are given for each species.

1a

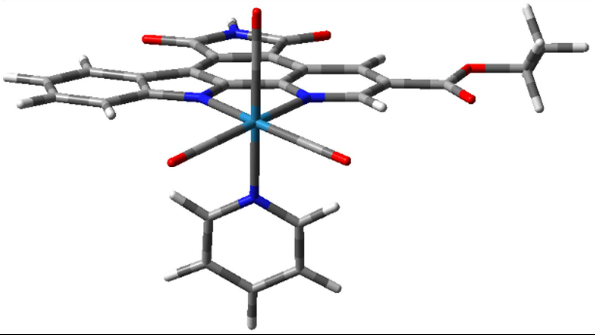

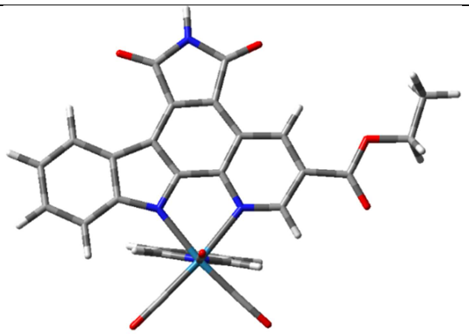

1b

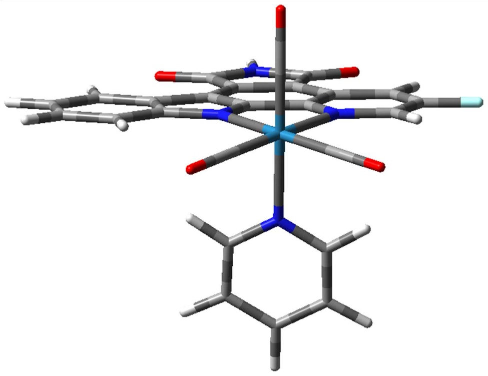

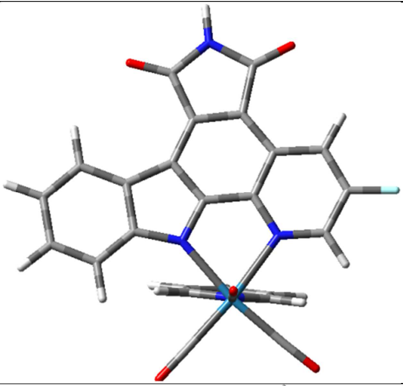

1c

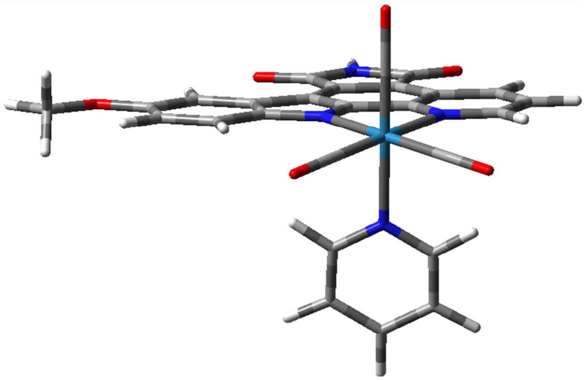

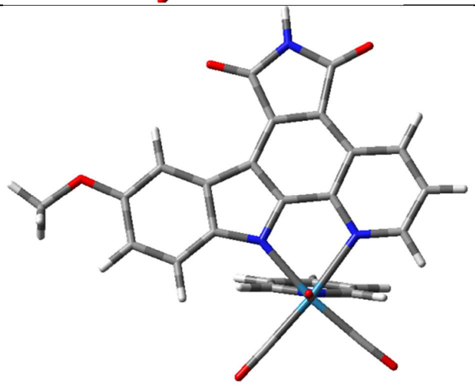

1d

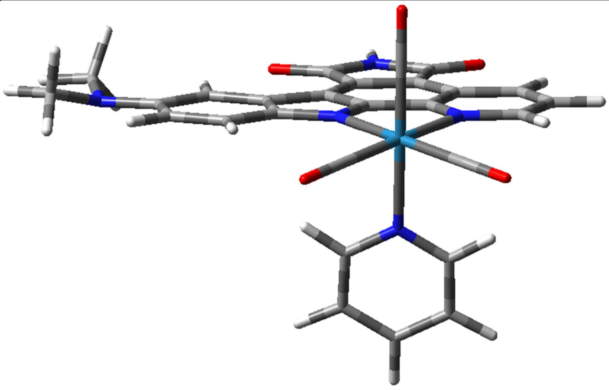

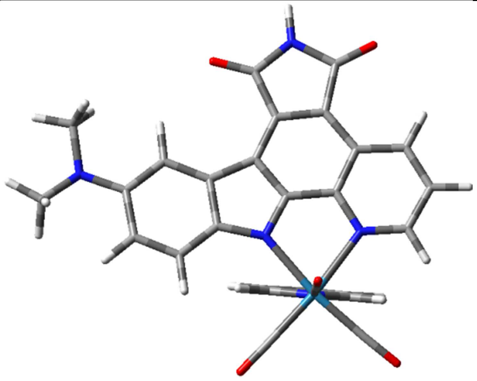

Figure S5. ***(cont.)***

1e

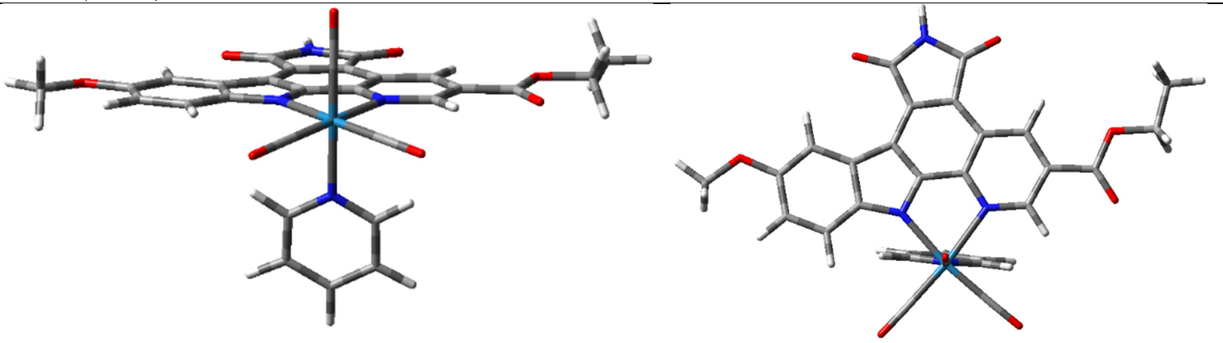

1f

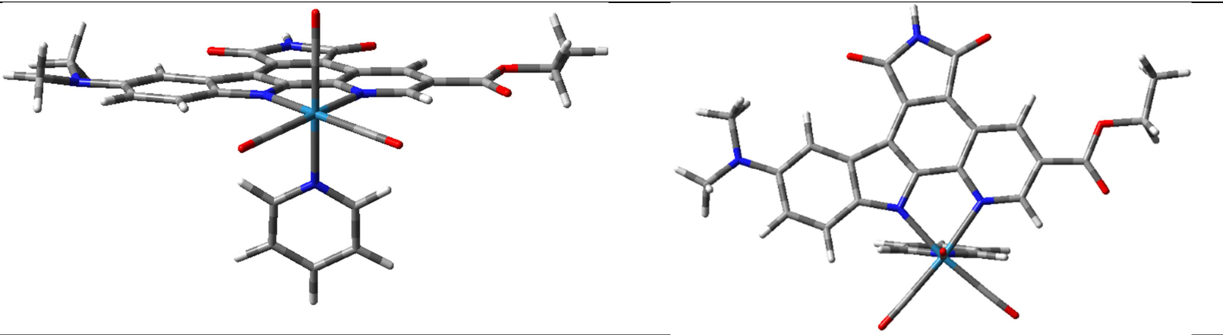

1g

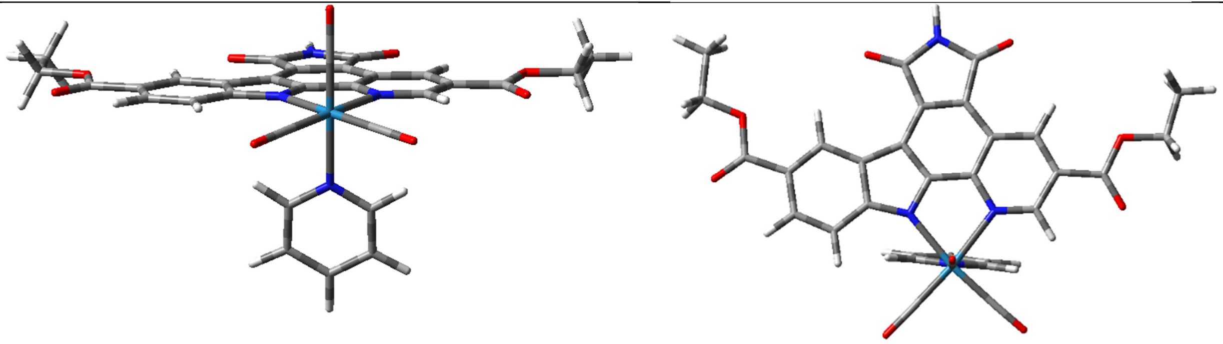

1h

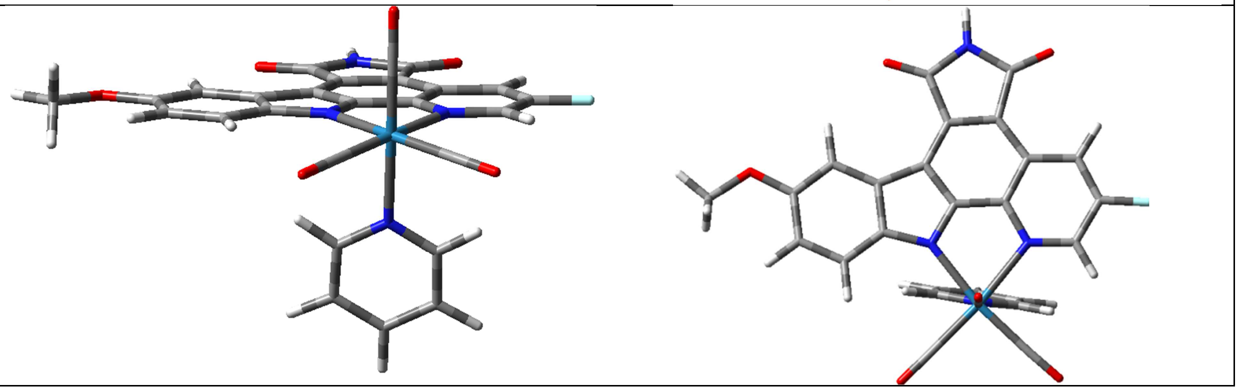

Figure S5. ***(cont.)***

1i

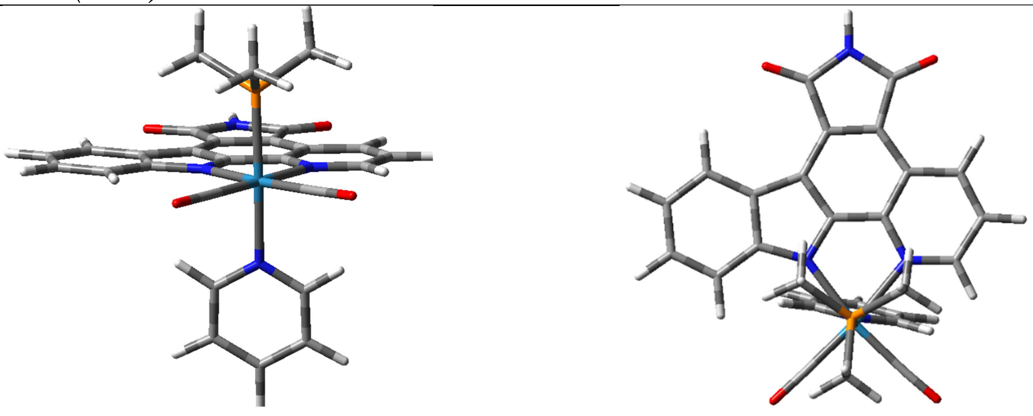

1j

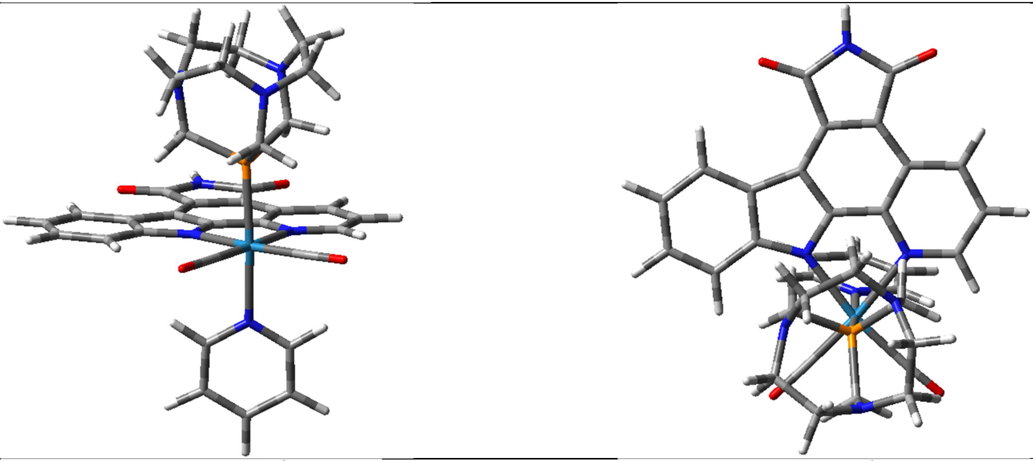

1k

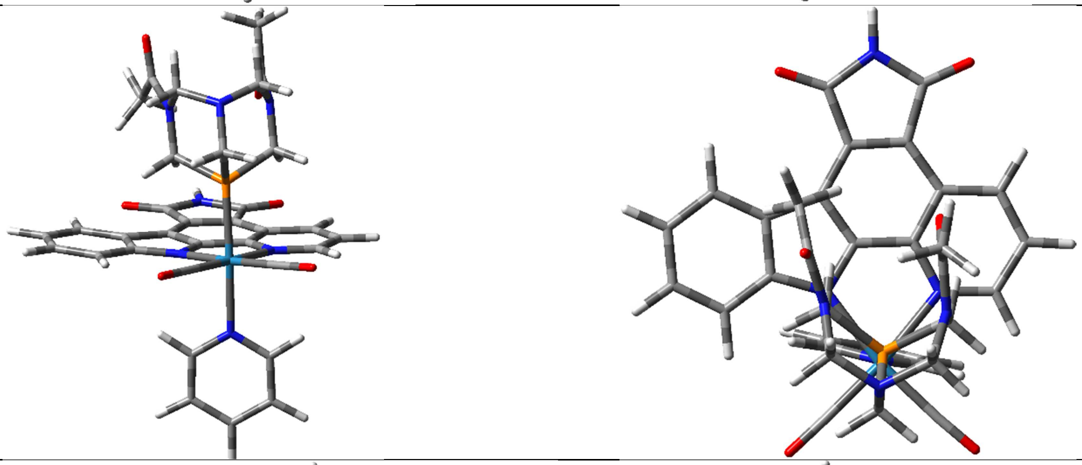

1l

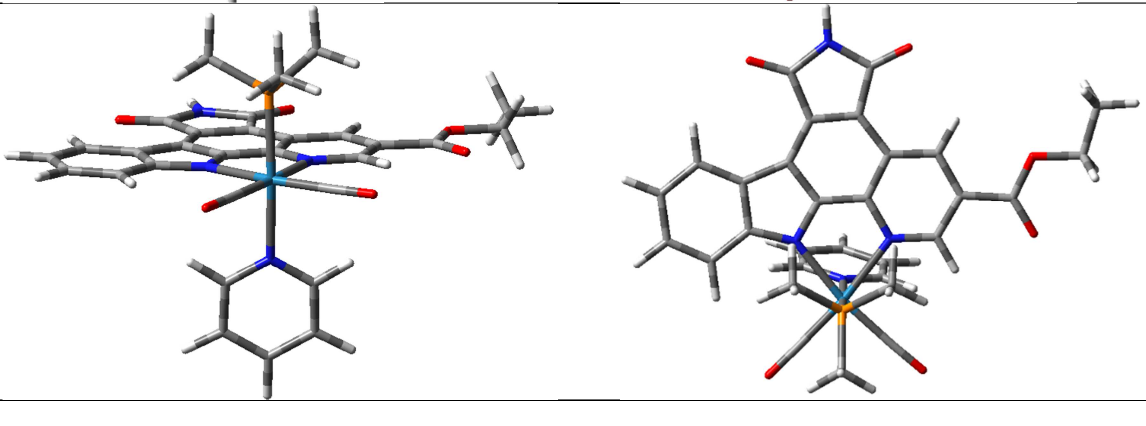

Figure S5. ***(cont.)***

1m

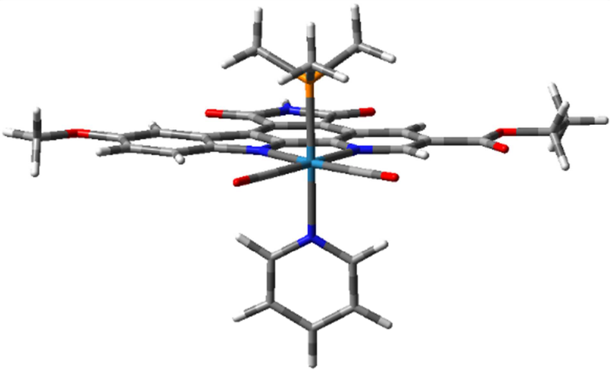

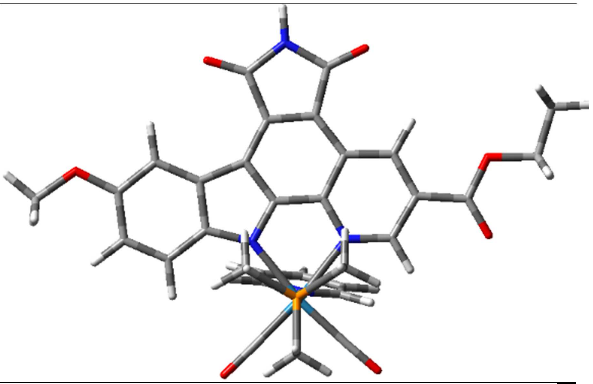

1n

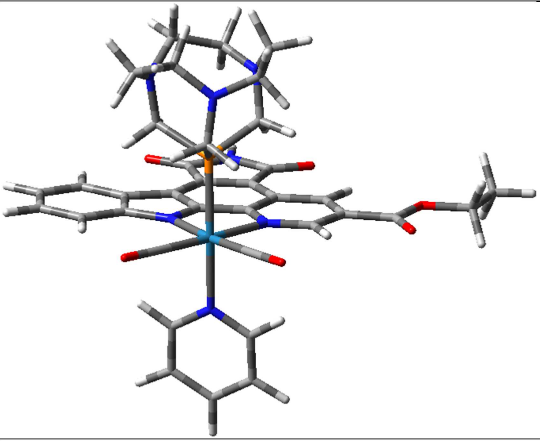

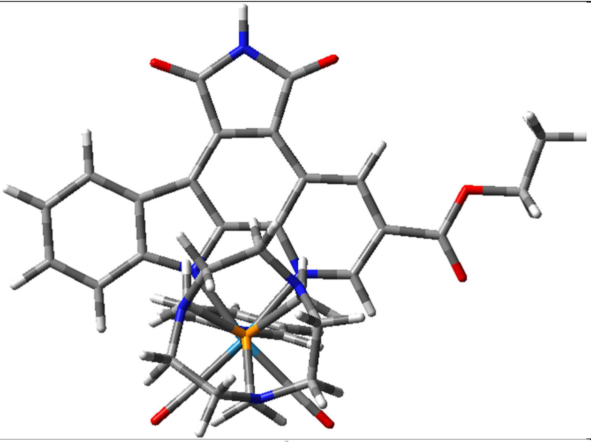

1o

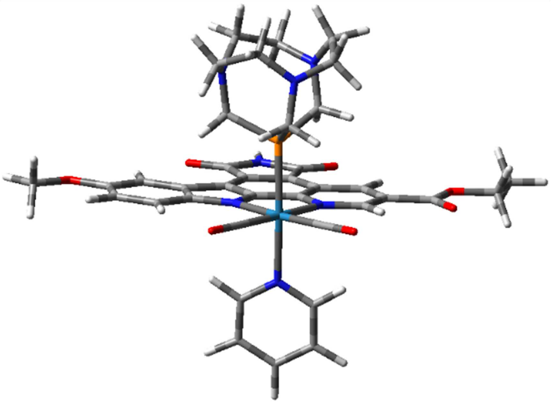

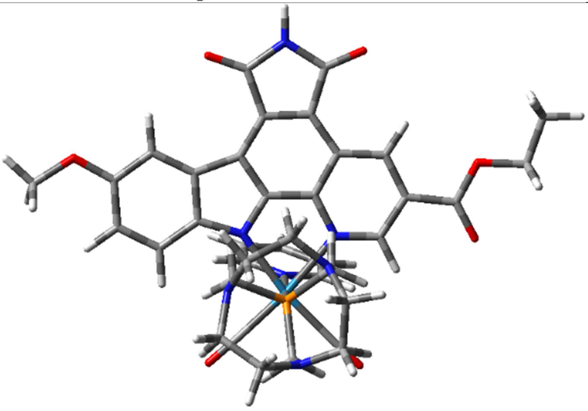

1p

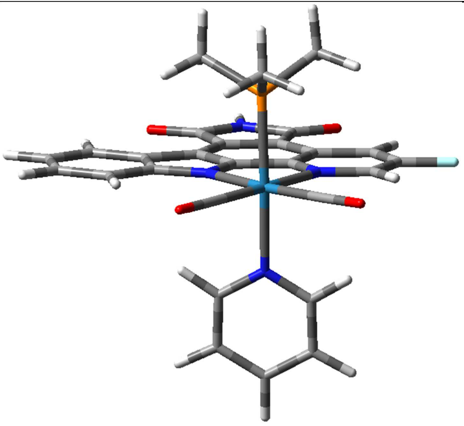

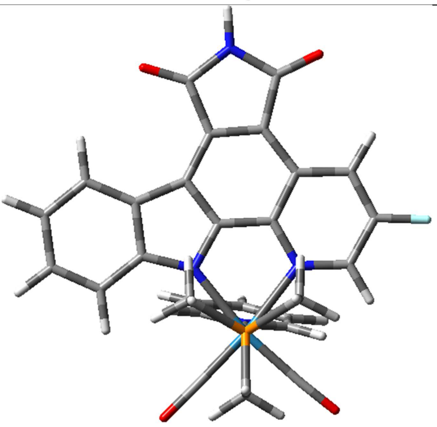

Figure S5. ***(cont.)***

1q

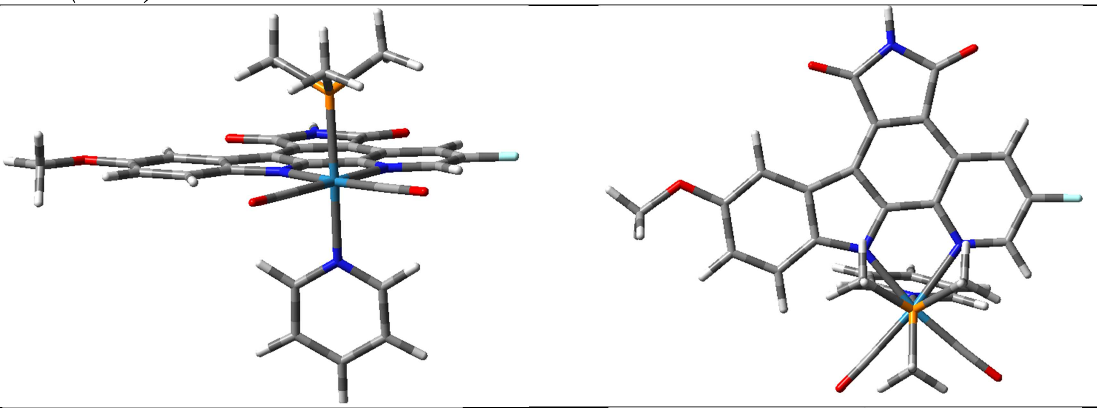

1r

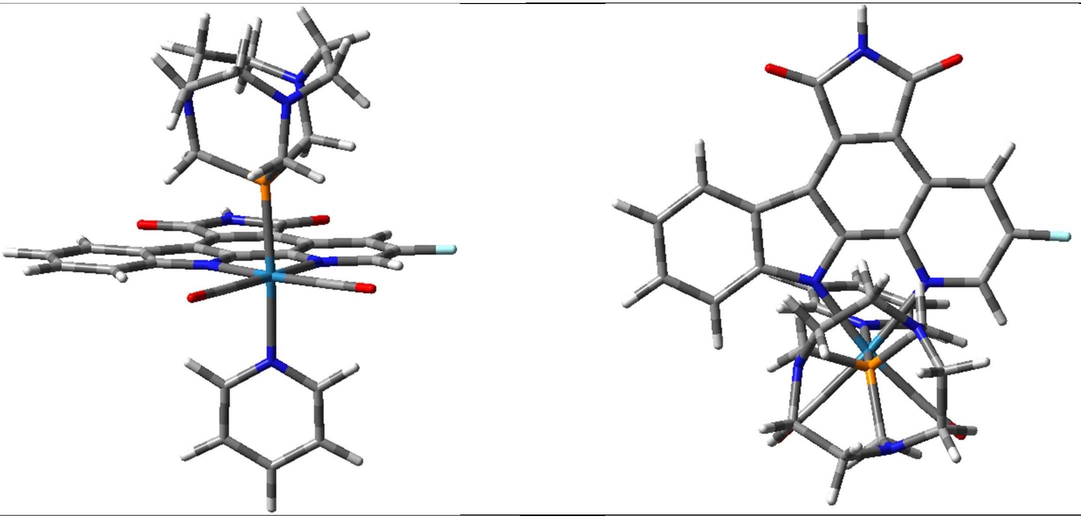

1s

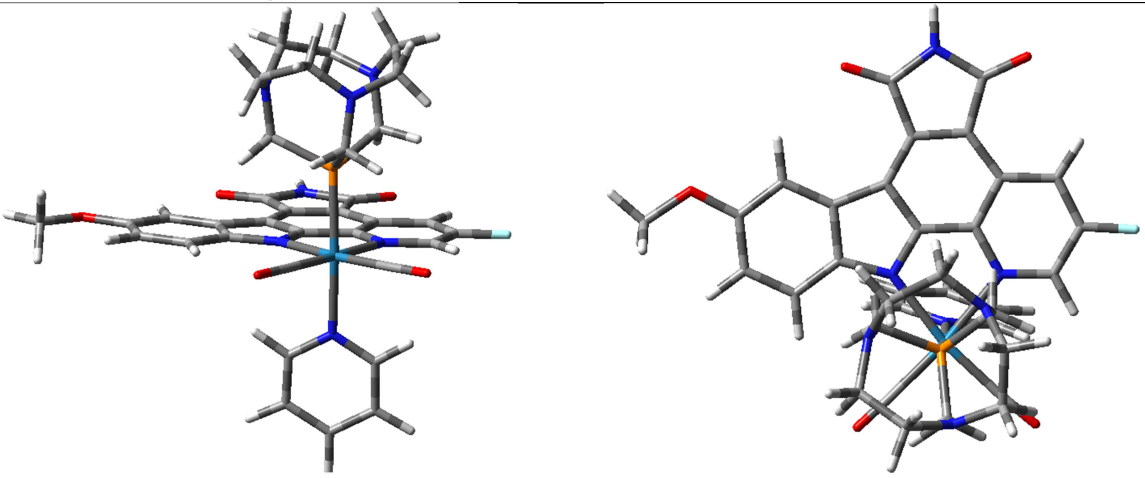

Figure S6. Contour maps of the spin density distribution (isovalue 0.0004) for the triplet excited state of the Re(I) complexes **1a**-**1s**. For clarity, two views are given for each species.

**1a**	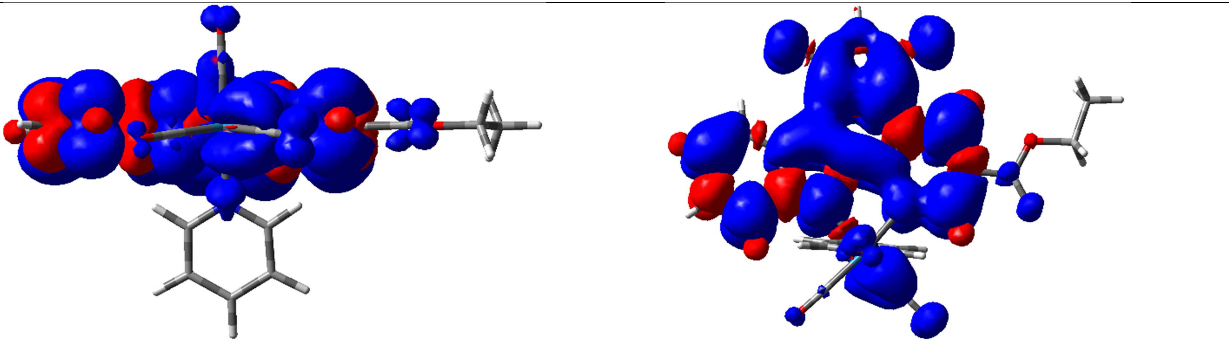
**1b**	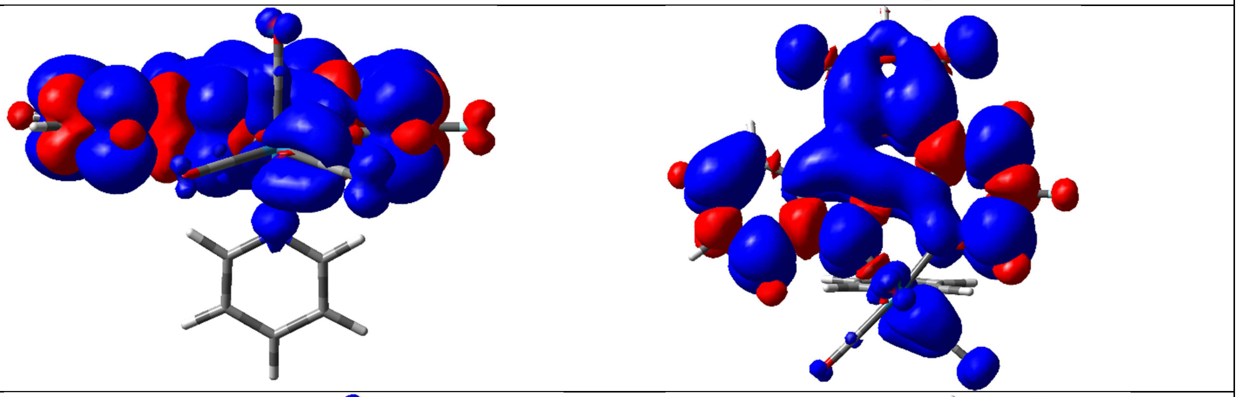
**1c**	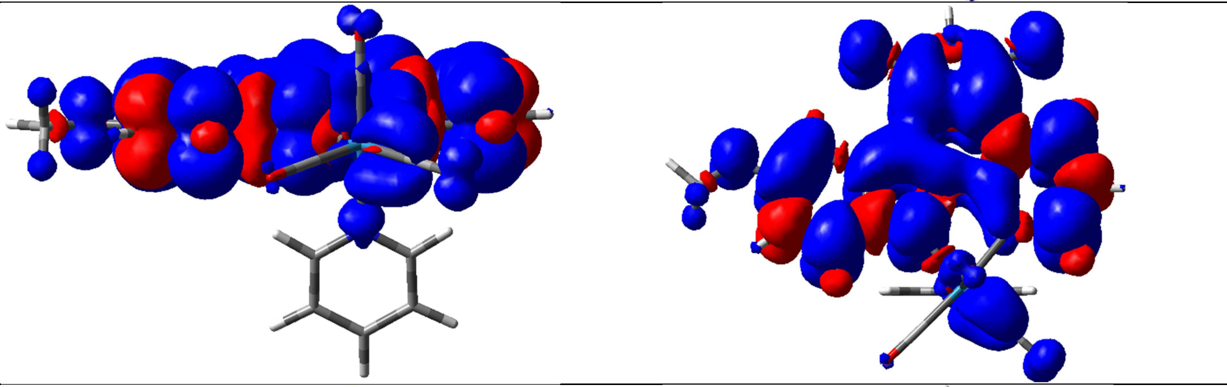
**1d**	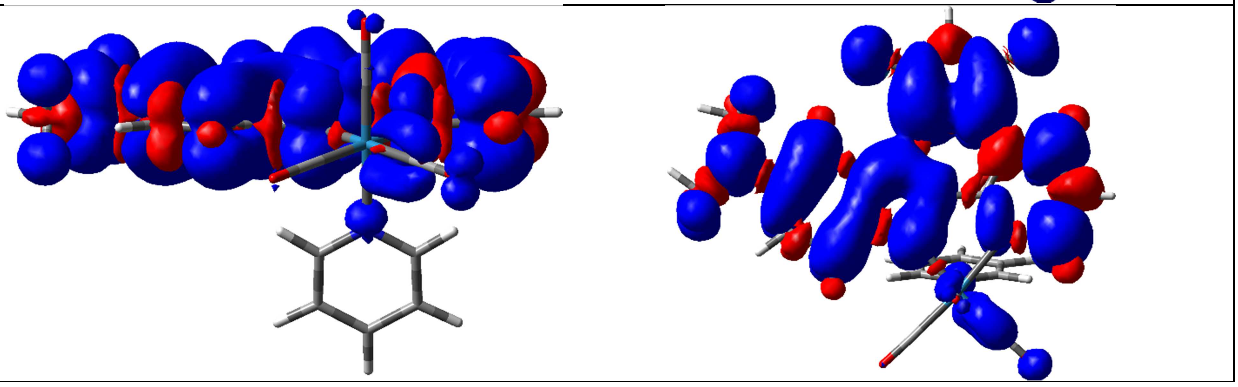

Figure S6. ***(cont.)***

1e

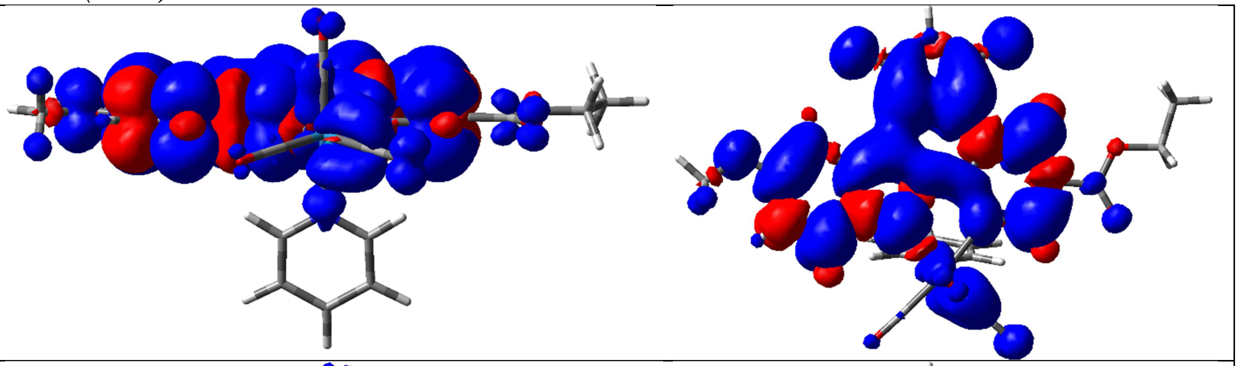

1f

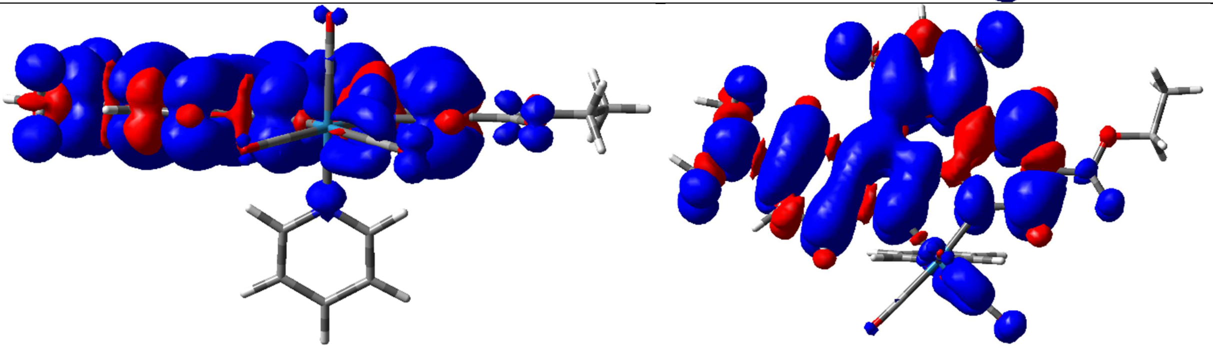

1g

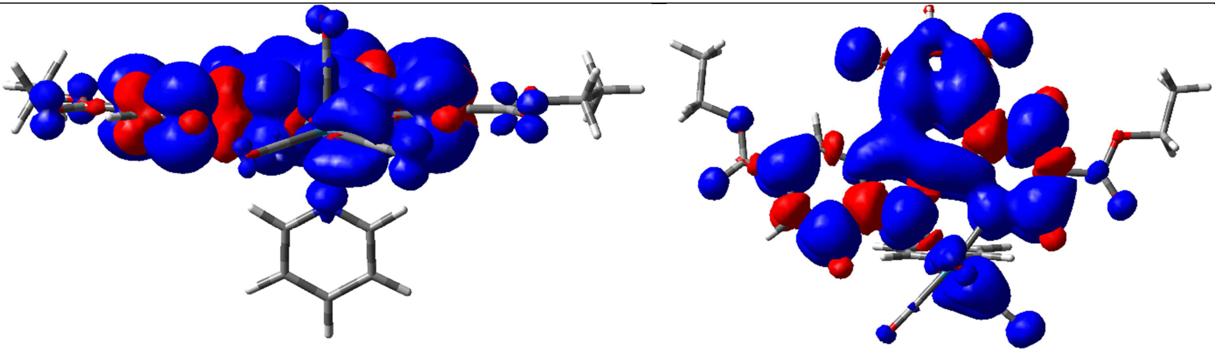

1h

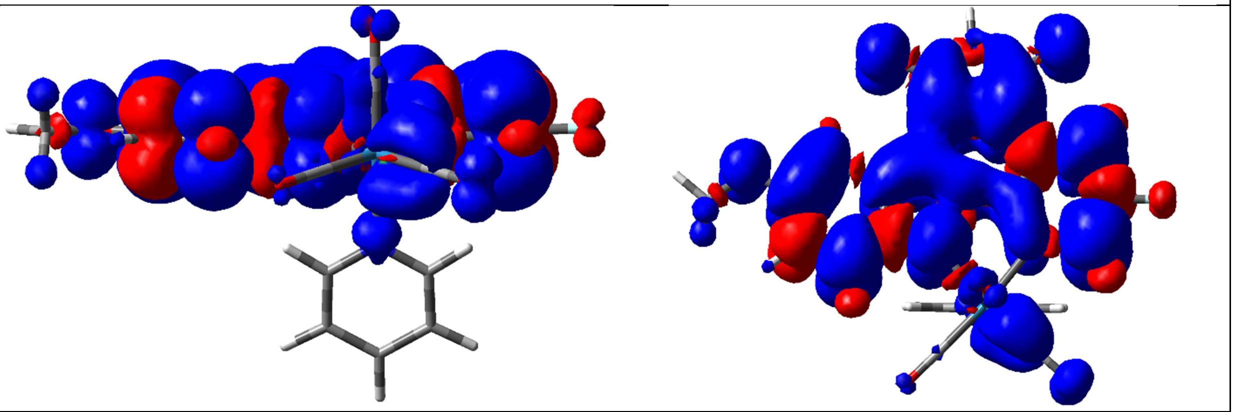

Figure S6. *(cont.)*

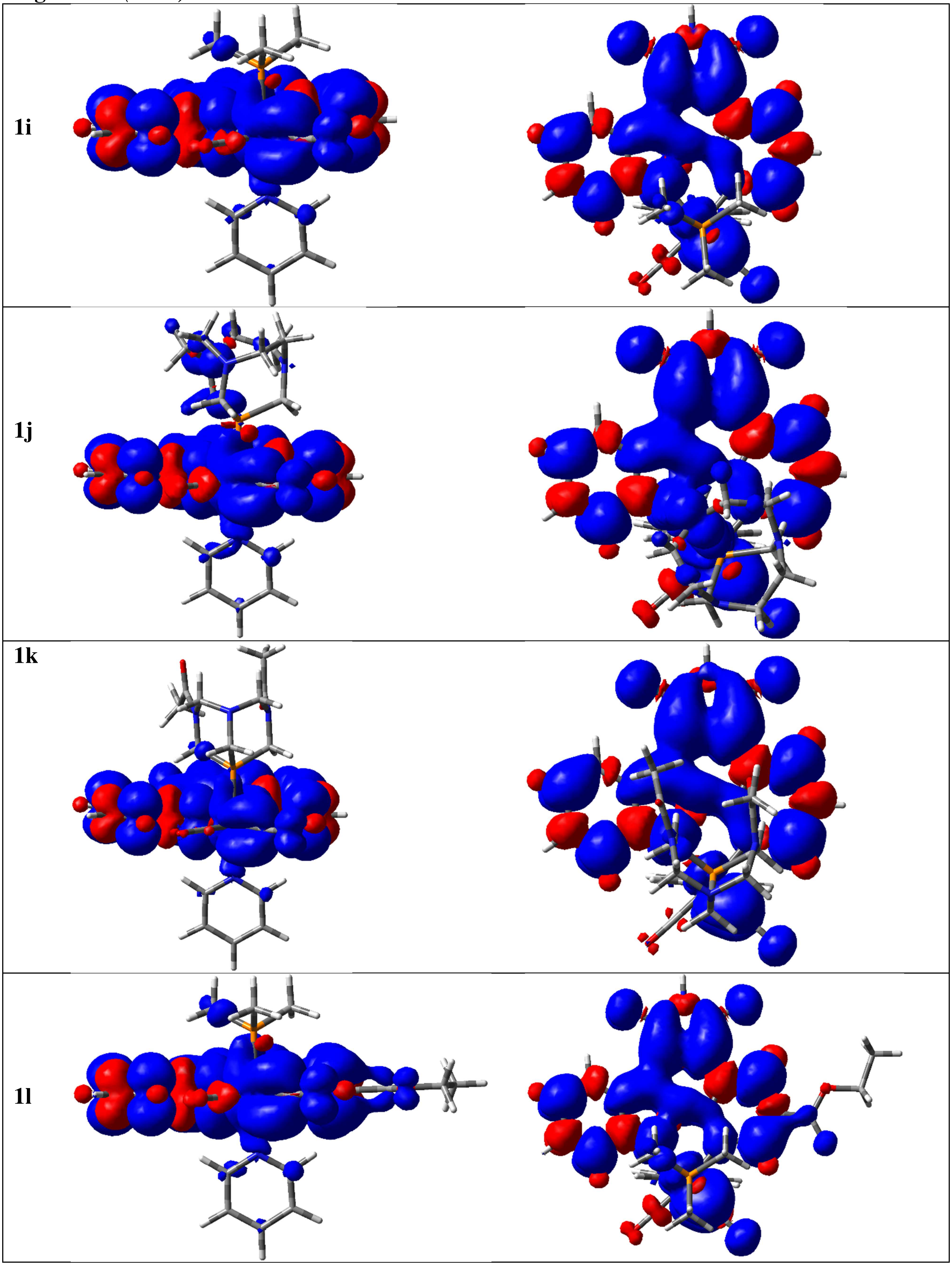

Figure S6. ***(cont.)***

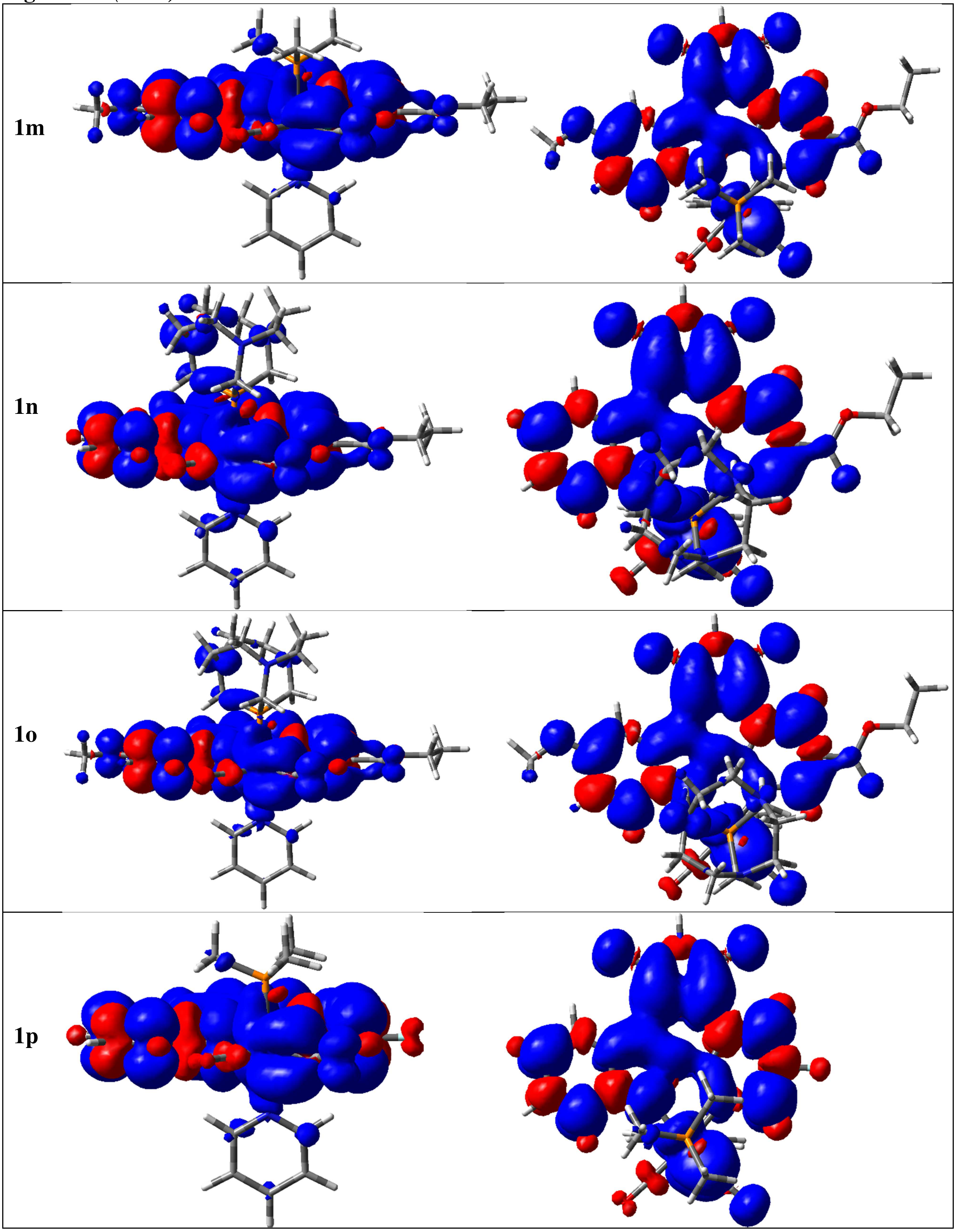

Figure S6. ***(cont.)***

1q

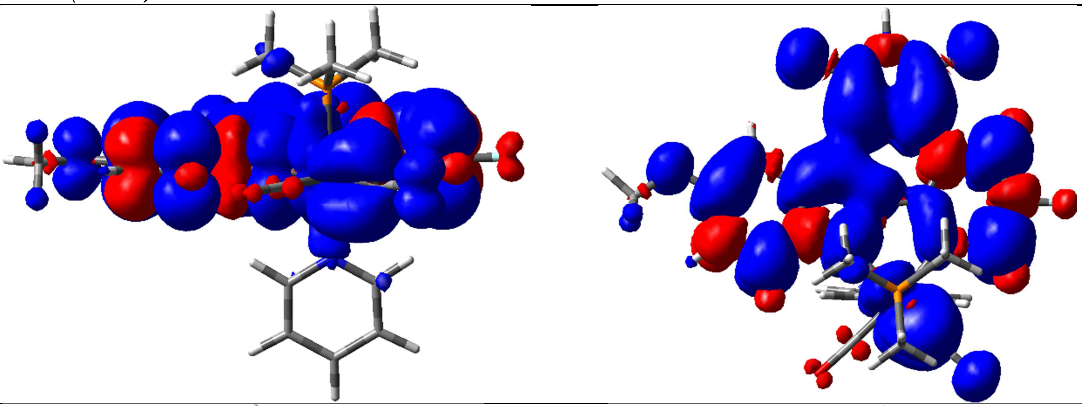

1r

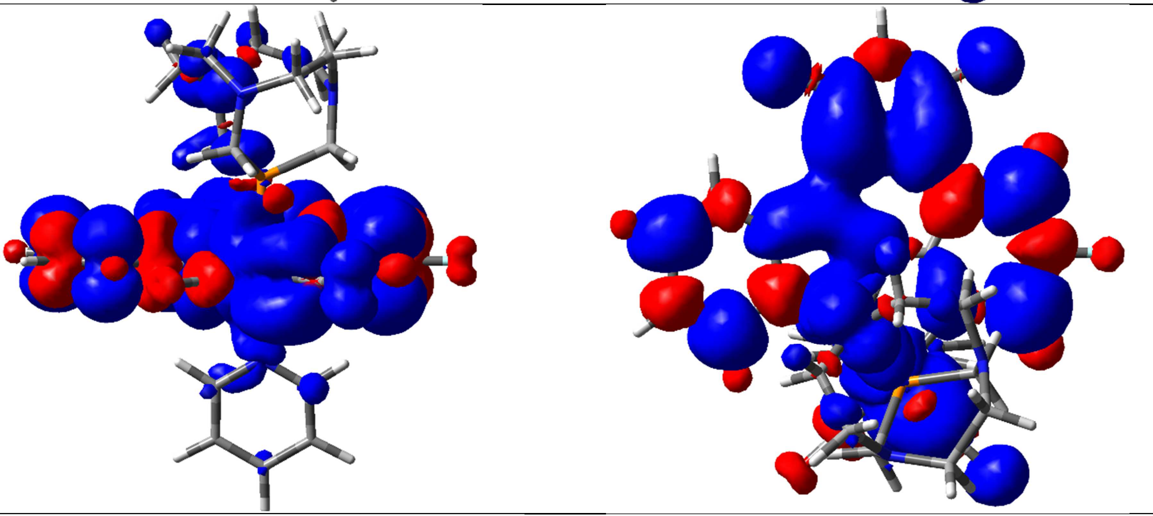

1s

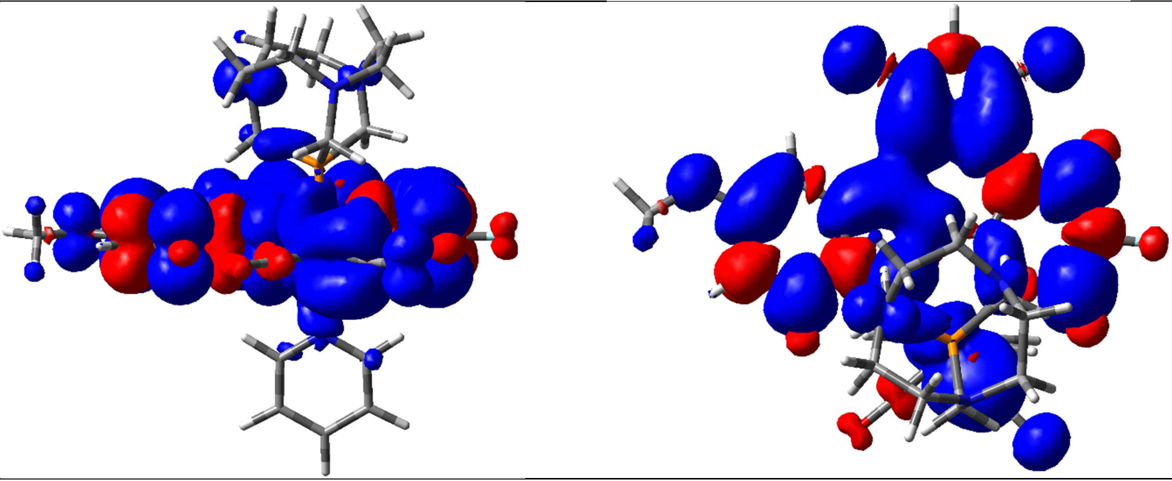

Table S13. B3LYP-D3/6-31+G(d)-LANL2DZ variations of some relevant bond distances (in Å), bond angles (in degrees), and dihedral angles (in degrees) of complexes **1a-1h** compared to complex **1**. The atom numbering used is collected for complex **1**.[a]

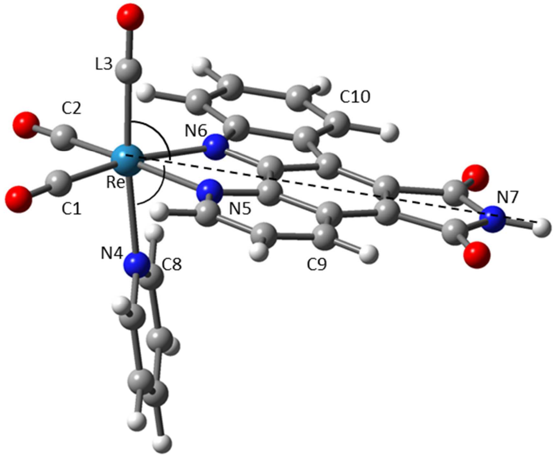

	bond distances						bond angles		dihedral angles	
Complexes	Re-C1	Re-C2	Re-L3[a]	Re-N4	Re-N5	Re-N6	N4-Re-N7	C3-Re-N7	C8-N4-Re-N7	C9-N5-N6-C10
**1a**	0.003	-0.001	0.000	0.002	0.003	-0.001	0.8	-0.2	1.3	-0.6
**1b**	0.001	0.000	0.001	0.000	0.003	-0.001	0.3	-0.1	1.0	-0.3
**1c**	0.001	0.000	0.000	-0.003	0.000	0.001	-0.3	0.4	1.1	0.0
**1d**	0.001	0.000	0.000	-0.004	0.000	0.001	-0.3	0.3	0.6	-0.1
**1e**	0.003	-0.002	0.000	-0.001	0.003	0.000	0.5	0.0	2.0	-0.8
**1f**	0.003	0.002	0.000	-0.002	0.003	-0.001	0.5	0.0	1.9	-0.7
**1g**	0.002	-0.001	0.001	0.004	0.003	-0.002	1.3	-0.7	0.3	-0.3
**1h**	0.002	-0.001	0.001	-0.003	0.003	0.000	0.1	0.5	1.8	-0.6

[a] L3 is the carbon atom of the carbonyl ligand in *trans* disposition to the pyridine ligand for complexes **1** and **1a-1h**.

Table S14. Excitation energies in eV (*E*) and nm (λ), and oscillator strengths (*f*) of the first ten lowest-lying singlet-singlet electron transitions calculated at the PCM-TD-M06/6-31+G(d)-LANL2DZ//B3LYP-D3/6-31+G(d)-LANL2DZ level for Re(I) complexes **1a**-**1h**. For comparison purposes, the data obtained for complex **1** are also included.

Transition		Complex								
		**1**	**1a**	**1b**	**1c**	**1d**	**1e**	**1f**	**1g**	**1h**
1	*E (λ)*	**2.38 (521)**	**2.32 (534)**	**2.33 (532)**	**2.19 (565)**	**1.88 (659)**	**2.14 (579)**	**1.83 (678)**	**2.40 (517)**	**2.14 (578)**
	f	**0.1459**	**0.1386**	**0.1435**	**0.1292**	**0.1007**	**0.1415**	**0.1312**	**0.1537**	**0.1292**
2	*E (λ)*	2.89 (428)	2.80 (443)	2.83 (439)	2.83 (439)	2.63 (471)	2.64 (470)	2.38 (521)	2.88 (430)	2.77 (448)
	f	0.0577	0.0083	0.0669	0.0490	0.0423	0.0048	0.0348	0.0100	0.0770
3	*E (λ)*	3.18 (390)	**2.96 (418)**	3.15 (394)	**2.98 (416)**	**2.74 (452)**	**2.84 (436)**	**2.64 (470)**	**3.05 (406)**	2.94 (421)
	f	0.0606	**0.1024**	0.0300	**0.0943**	**0.1465**	**0.1196**	**0.1325**	**0.1053**	0.0397
4	*E (λ)*	3.36 (369)	3.25 (381)	3.29 (377)	3.26 (381)	2.96 (419)	3.24 (383)	2.98 (416)	3.24 (383)	3.28 (379)
	f	0.0062	0.0030	0.0156	0.0063	0.0035	0.0052	0.0035	0.0075	0.0221
5	*E (λ)*	3.44 (361)	3.33 (372)	3.36 (369)	3.36 (369)	3.30 (376)	3.29 (376)	3.19 (389)	3.31 (375)	3.29 (376)
	f	0.0058	0.0037	0.0008	0.0091	0.0303	0.0032	0.0300	0.0018	0.0042
6	*E (λ)*	3.45 (359)	3.37 (368)	3.40 (364)	3.43 (361)	3.40 (365)	3.32 (373)	3.25 (381)	3.35 (370)	3.35 (371)
	f	0.0025	0.0139	0.0145	0.0131	0.0247	0.0100	0.0333	0.0185	0.0166
7	*E (λ)*	3.49 (356)	3.48 (356)	3.48 (356)	3.48 (357)	3.43 (362)	3.37 (368)	**3.26 (380)**	3.61 (344)	3.39 (366)
	f	0.0146	0.0150	0.0137	0.0038	0.0601	0.0037	**0.1678**	0.0308	0.0066
8	*E (λ)*	3.78 (328)	**3.61 (343)**	**3.76 (330)**	3.64 (340)	3.47 (357)	**3.50 (354)**	**3.35 (370)**	**3.66 (339)**	3.62 (343)
	f	0.0056	**0.4810**	**0.2128**	0.0119	0.0473	**0.4735**	**0.1614**	**0.4517**	0.0633
9	*E (λ)*	**3.79 (327)**	3.70 (335)	3.78 (328)	3.67 (338)	3.48 (356)	3.52 (352)	3.38 (367)	3.76 (330)	3.66 (339)
	f	**0.2133**	0.0882	0.0101	0.0660	0.0075	0.0457	0.0406	0.0002	0.0835
10	*E (λ)*	3.81 (325)	3.77 (329)	3.80 (327)	**3.70 (335)**	**3.54 (351)**	3.69 (336)	3.48 (356)	3.77 (329)	**3.68 (337)**
	f	0.0006	0.0002	0.0005	**0.2316**	**0.1401**	0.0050	0.0089	0.0181	**0.1738**

Table S15. B3LYP-D3/6-31+G(d)-LANL2DZ variations of some relevant bond distances (in Å), bond angles (in degrees), and dihedral angles (in degrees) of complexes **1i-1s** compared to complex **1**. The atom numbering used is collected for complex **1**.[a]

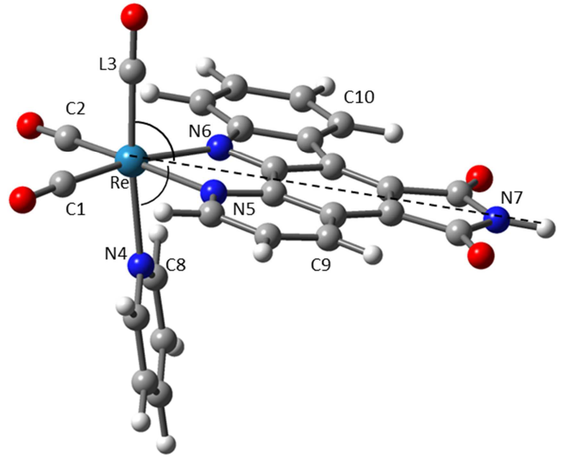

	bond distances						bond angles		dihedral angles	
Complexes	Re-C1	Re-C2	Re-L3[a]	Re-N4	Re-N5	Re-N6	N4-Re-N7	C3-Re-N7	C8-N4-Re-N7	C9-N5-N6-C10
**1i**	-0.026	-0.024	0.450	-0.034	0.007	0.013	5.9	-8.2	3.6	-3.3
**1j**	-0.027	-0.024	0.451	-0.033	0.009	0.018	7.0	-10.0	3.4	-3.2
**1k**	-0.022	-0.021	0.428	-0.042	0.012	0.016	12.2	-16.3	4.4	-7.2
**1l**	-0.023	-0.025	0.449	-0.035	0.008	0.015	6.2	-8.4	3.7	-3.4
**1m**	-0.023	-0.025	0.449	-0.035	0.008	0.012	5.9	-8.2	4.4	-3.2
**1n**	-0.024	-0.025	0.452	-0.034	0.010	0.019	7.4	-10.3	3.7	-3.2
**1o**	-0.024	-0.026	0.451	-0.033	0.010	0.017	6.8	-9.8	4.4	-3.3
**1p**	-0.025	-0.024	0.451	-0.033	0.006	0.013	5.9	-8.0	4.2	-3.3
**1q**	-0.024	-0.024	0.451	-0.033	0.007	0.010	5.8	-8.1	4.9	-3.6
**1r**	-0.026	-0.024	0.454	-0.032	0.008	0.018	6.6	-9.6	4.0	-3.1
**1s**	-0.026	-0.025	0.453	-0.032	0.009	0.015	7.0	-10.0	5.1	-3.3

[a] L3 is the carbon atom of the carbonyl ligand in *trans* disposition to the pyridine ligand for complex **1** and the phosphorus atom of the phosphine ligand in *trans* disposition to the pyridine ligand for complexes **1i**-**1s**.

Table S16. Excitation energies in eV (*E*) and nm (λ), and oscillator strengths (*f*) of the first ten lowest-lying singlet-singlet electron transitions calculated at the PCM-TD-M06/6-31+G(d)-LANL2DZ//B3LYP-D3/6-31+G(d)-LANL2DZ level for Re(I) complexes **1i**-**1s**. For comparison purposes, the data obtained for complex **1** are also included.

Transition		**1**	**1i**	**1j**	**1k**	**1l**	**1m**	**1n**	**1o**	**1p**	**1q**	**1r**	**1s**
1	*E* (λ)	**2.38 (521)**	**2.12 (584)**	**2.03 (611)**	**2.22 (559)**	**2.04 (608)**	**1.96 (631)**	**1.92 (644)**	**1.90 (653)**	**2.06 (601)**	**1.98 (627)**	**1.96 (632)**	**1.92 (645)**
	f	**0.1459**	**0.0979**	**0.0767**	**0.1017**	**0.0945**	**0.1161**	**0.0698**	**0.0934**	**0.0860**	**0.0995**	**0.0651**	**0.0822**
2	*E* (λ)	2.89 (428)	2.47 (503)	2.14 (579)	2.56 (484)	2.37 (523)	2.38 (520)	2.02 (614)	2.04 (607)	**2.41 (514)**	2.43 (510)	2.06 (603)	2.08 (596)
	f	0.0577	0.0557	0.0083	0.0323	0.0580	0.0374	0.0076	0.0111	**0.0791**	0.0674	0.0129	0.0154
3	*E* (λ)	3.18 (390)	2.78 (446)	2.28 (545)	2.81 (441)	2.57 (483)	2.45 (505)	2.16 (575)	2.17 (571)	2.73 (454)	2.64 (471)	2.20 (564)	2.21 (560)
	f	0.0606	0.0297	0.0232	0.0309	0.0069	0.0248	0.0276	0.0256	0.0394	0.0461	0.0296	0.0273
4	*E* (λ)	3.36 (369)	2.94 (422)	2.56 (485)	2.99 (414)	2.78 (446)	2.67 (465)	2.45 (505)	2.35 (528)	2.86 (434)	2.79 (445)	2.49 (497)	2.39 (519)
	f	0.0062	0.0003	0.0472	0.0408	0.0275	0.0497	0.0340	0.0275	0.0005	0.0168	0.0527	0.0359
5	*E* (λ)	3.44 (361)	2.96 (418)	2.80 (443)	3.03 (409)	2.83 (438)	2.85 (436)	2.51 (494)	2.43 (510)	2.91 (426)	2.87 (432)	2.76 (450)	2.70 (459)
	f	0.0058	0.0603	0.0027	0.0305	0.0015	0.0024	0.0146	0.0168	0.0541	0.0441	0.0255	0.0011
6	*E* (λ)	3.45 (359)	3.00 (414)	**2.90 (428)**	3.06 (405)	2.94 (422)	2.87 (433)	2.68 (463)	2.67 (465)	2.97 (418)	2.88 (430)	2.83 (437)	**2.80 (442)**
	f	0.0025	0.0336	**0.0870**	0.0111	0.0367	0.0475	0.0029	0.0038	0.0167	0.0181	0.0540	**0.0929**
7	*E* (λ)	3.49 (356)	3.19 (389)	2.93 (423)	3.31 (374)	3.02 (410)	3.00 (413)	2.79 (445)	2.77 (448)	3.21 (386)	3.14 (395)	2.85 (435)	2.87 (432)
	f	0.0146	0.0256	0.0002	0.0372	0.0450	0.0236	0.0006	0.0002	0.0255	0.0220	0.0056	0.0007
8	*E* (λ)	3.78 (328)	3.32 (374)	3.01 (412)	3.40 (364)	3.19 (389)	3.12 (398)	2.80 (443)	2.80 (443)	3.32 (373)	3.31 (375)	2.94 (421)	2.93 (423)
	f	0.0056	0.0368	0.0006	0.0250	0.0202	0.0178	0.0463	0.0632	0.0318	0.0267	0.0001	0.0081
9	*E* (λ)	**3.79 (327)**	3.49 (356)	3.04 (408)	**3.60 (344)**	3.36 (369)	3.26 (380)	2.82 (440)	2.84 (437)	3.52 (352)	3.47 (358)	2.99 (414)	2.99 (415)
	f	**0.2133**	0.0791	0.0046	**0.0910**	0.0205	0.0091	0.0033	0.0027	0.0680	0.0921	0.0035	0.0019
10	*E* (λ)	3.81 (325)	3.63 (342)	3.11 (398)	3.67 (338)	**3.47 (357)**	3.35 (370)	2.93 (423)	2.92 (425)	**3.58 (346)**	**3.53 (351)**	3.10 (401)	3.03 (409)
	f	0.0006	0.0776	0.0274	0.0174	**0.0921**	0.0661	0.0035	0.0173	**0.1234**	**0.1008**	0.0253	0.0237

Figure S7. PCM-TD-M06/6-31+G(d)-LANL2DZ//B3LYP-D3/6-31+G(d)-LANL2DZ electronic absorption spectra of Re(I) pyridocarbazole complexes bearing the phosphine ligands DAPTA (**1k**), PMe_3 (**1p** and **1q**), and CAP (**1r** and **1s**).

1k
(R^1 = H, R^2 = H, R^3 = DAPTA)

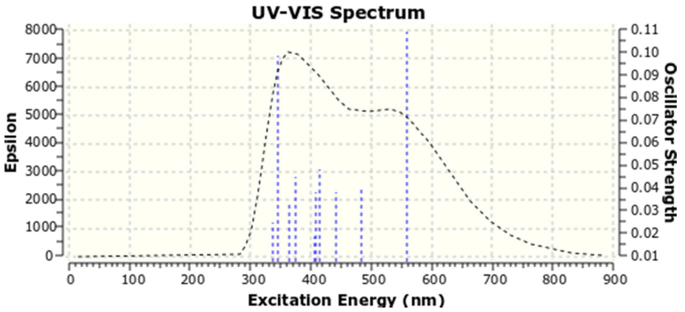

1p
(R^1 = F, R^2 = H, R^3 = PMe_3)

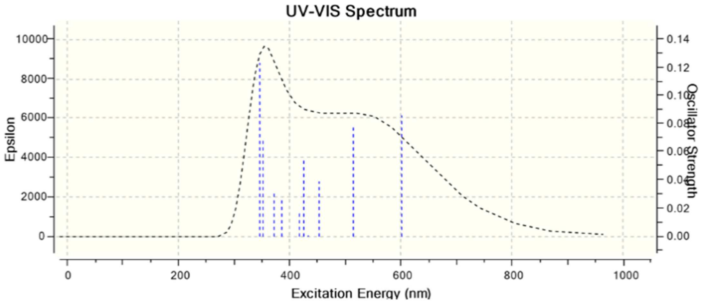

1q
(R^1 = F, R^2 = OMe, R^3 = PMe_3)

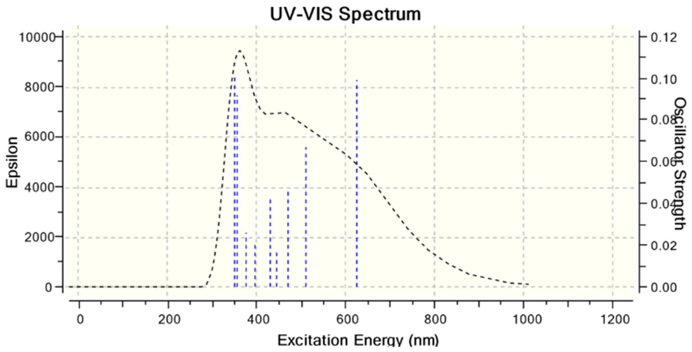

1r
(R^1 = F, R^2 = H, R^3 = CAP)

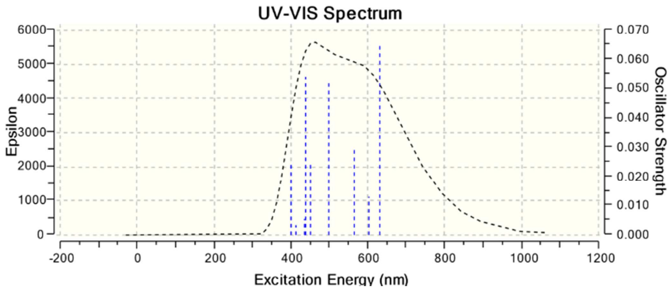

1s
(R^1 = F, R^2 = OMe, R^3 = CAP)

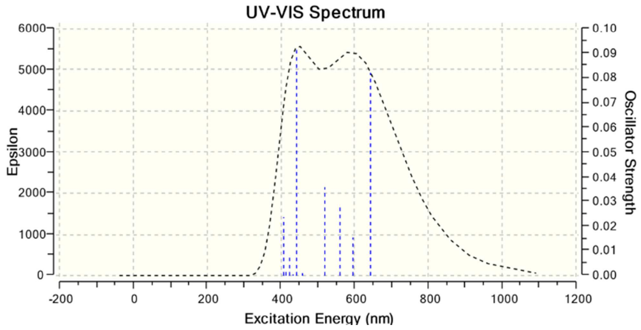

Figure S8. Contour maps of the frontier Kohn-Sham orbitals involved in the main orbital transition of the lowest-lying absorption band found for Re(I) dicarbonyl pyridyl complexes containing the phosphine ligands DAPTA (**1k**), PMe_3 (**1p** and **1q**), and CAP (**1r** and **1s**). Two views are given for each orbital.

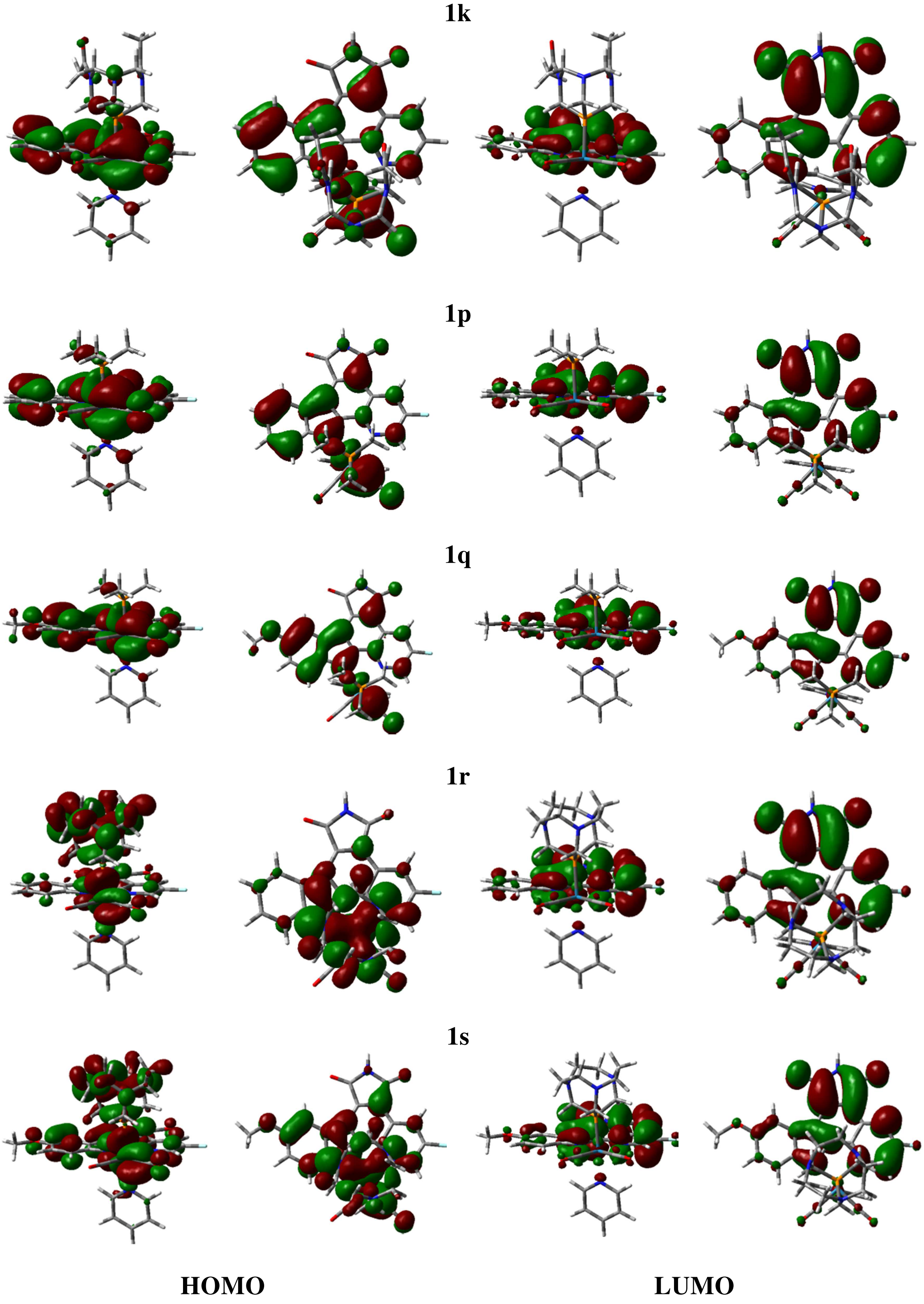

Table S17. B3LYP-D3/6-31+G(d)-LANL2DZ cartesian coordinates, in Å, of the optimized structures in their singlet ground states of all Re(I) complexes investigated.

1

Re	9.134012	12.089196	15.332679
C	9.083914	12.944592	13.605011
O	9.074238	13.414479	12.538489
C	9.452572	13.769912	16.213463
O	9.672116	14.771443	16.761677
N	11.342310	11.642385	15.167651
C	11.898691	11.263161	13.998702
C	13.241917	10.920350	13.879223
C	14.055653	10.972353	15.012512
C	13.486411	11.369276	16.223076
C	12.133088	11.693203	16.260900
N	8.957802	10.007973	14.492680
C	8.749986	9.607944	13.236181
C	8.749476	8.247245	12.870354
C	8.971222	7.264446	13.824230
C	9.186245	7.650049	15.168340
C	9.162706	9.048401	15.445443
C	9.350228	9.506201	16.765896
N	9.326279	10.816304	17.092886
C	9.539950	10.861591	18.466626
C	9.611653	11.987184	19.298085
C	9.855544	11.780848	20.654879
C	10.024532	10.483429	21.186835
C	9.952930	9.356690	20.370928
C	9.711429	9.537404	18.999743
C	9.580637	8.645785	17.874755
C	9.614007	7.275714	17.580189
C	9.837675	6.101065	18.484233
O	10.046052	6.088101	19.684150
N	9.762545	4.983106	17.653802
C	9.515966	5.319304	16.308127
O	9.413244	4.512604	15.396785
C	9.423900	6.792236	16.281479
H	11.235271	11.233241	13.142041
H	13.633385	10.618752	12.912953
H	15.108127	10.710197	14.952726
H	14.072510	11.426179	17.134611
H	11.653966	11.991153	17.185481
H	8.576246	10.389046	12.502838
H	8.978628	6.210789	13.564244
H	9.471317	12.985635	18.895269
H	9.911344	12.639188	21.319673
H	10.078725	8.354866	20.769575
H	9.871064	4.031616	17.979931
C	7.225199	12.297771	15.537627
O	6.077386	12.400079	15.665115
H	8.571583	7.983527	11.832666
H	10.209748	10.364663	22.250961

2

Re	1.912802	-0.109845	-0.489261
C	3.366083	-1.374133	-0.407931
O	4.211099	-2.175308	-0.336069
C	3.110805	1.373772	-0.239972
O	3.816019	2.281935	-0.062831
N	1.499648	-0.279910	1.727291
C	1.604756	-1.458266	2.374687
C	1.303388	-1.605960	3.725132
C	0.877149	-0.489985	4.447787
C	0.770166	0.732142	3.783184
C	1.086387	0.796680	2.428795
N	0.324876	-1.705979	-0.596054
C	0.439575	-3.031505	-0.702727
C	-0.682113	-3.882865	-0.678826
C	-1.957504	-3.349732	-0.541595
C	-2.128601	-1.951369	-0.434322
C	-0.931000	-1.177692	-0.472231
C	-1.024946	0.217753	-0.386651
N	0.061628	1.035618	-0.428157
C	-0.446574	2.318033	-0.324976
C	0.250431	3.536985	-0.308205
C	-0.484043	4.711963	-0.173828
C	-1.893202	4.693307	-0.059022
C	-2.594511	3.490826	-0.076685
C	-1.882998	2.287043	-0.207235
C	-2.253905	0.893530	-0.254624
C	-3.441097	0.118020	-0.208171
C	-3.379199	-1.264612	-0.296007
H	1.936996	-2.301058	1.779526
H	1.403377	-2.580447	4.192469
H	0.634663	-0.571740	5.503605
H	0.441943	1.630585	4.295821
H	0.998819	1.723454	1.875311
H	1.445459	-3.424059	-0.814011
H	-2.826763	-4.003261	-0.521655
H	1.331866	3.556613	-0.407357
H	0.036988	5.666159	-0.162846
H	-3.678801	3.483411	0.009465
H	-4.408939	0.603603	-0.107336
H	-4.291857	-1.853969	-0.264674
C	2.080281	0.023845	-2.406599
O	2.158135	0.099372	-3.561650
H	-0.531868	-4.953770	-0.772040
H	-2.432619	5.631561	0.040532

3

Re	1.820259	-0.091171	-0.490398
C	3.298898	-1.327889	-0.414143
O	4.152399	-2.121667	-0.347499
C	3.033174	1.382911	-0.228592
O	3.757876	2.273790	-0.042271
N	1.424284	-0.268688	1.732475
C	1.574017	-1.440772	2.382344
C	1.292765	-1.593191	3.736706
C	0.839811	-0.488256	4.460342
C	0.686019	0.727121	3.792670
C	0.984230	0.796663	2.434192
N	0.249030	-1.661020	-0.596894
C	0.477123	-2.983503	-0.742787
C	-0.536171	-3.930320	-0.732205
C	-1.858404	-3.489132	-0.562396
C	-2.106920	-2.133355	-0.423049
C	-1.036765	-1.217215	-0.447856
C	-1.194705	0.211324	-0.340296
N	-0.049393	0.984102	-0.428798
C	-0.454261	2.289383	-0.336551
C	0.314308	3.470028	-0.368694
C	-0.345006	4.684000	-0.233194
C	-1.753981	4.749886	-0.068737
C	-2.523272	3.597038	-0.039118
C	-1.883344	2.344116	-0.170971
C	-2.336495	0.998992	-0.179738
H	1.928617	-2.274485	1.787052
H	1.430027	-2.561952	4.206547
H	0.613107	-0.573366	5.519436
H	0.335651	1.616783	4.305967
H	0.861195	1.718194	1.878356
H	1.514413	-3.271766	-0.875456
H	-2.679458	-4.200715	-0.547511
H	1.389778	3.431382	-0.506348
H	0.228931	5.606980	-0.259999
H	-3.602769	3.652115	0.082925
H	-3.358681	0.653950	-0.089891
H	-3.117428	-1.758444	-0.301226
C	1.984902	0.057762	-2.405890
O	2.062341	0.142686	-3.560685

H	-0.296801	-4.981009	-0.857381
H	-2.231173	5.721604	0.030264

1a

Re	1.353175	-1.620688	-0.477307
C	0.795825	-3.463266	-0.330599
O	0.425605	-4.561040	-0.217314
C	3.205072	-2.126861	-0.368338
O	4.329621	-2.407876	-0.276030
N	1.203063	-1.290394	1.751290
C	0.220755	-1.859268	2.480987
C	0.066664	-1.630697	3.844841
C	0.965246	-0.781466	4.493396
C	1.986235	-0.195079	3.744390
C	2.070960	-0.471046	2.382583
N	-0.741219	-0.790257	-0.423610
C	-1.908033	-1.427277	-0.404053
C	-3.141708	-0.743628	-0.285842
C	-3.163542	0.644703	-0.187905
C	-1.941748	1.351193	-0.206885
C	-0.750923	0.577233	-0.329508
C	0.501713	1.217204	-0.361680
N	1.664367	0.539842	-0.480519
C	2.660705	1.508338	-0.478568
C	4.047651	1.328233	-0.573967
C	4.855928	2.462135	-0.530982
C	4.308006	3.758077	-0.397982
C	2.932448	3.950956	-0.305004
C	2.094637	2.824035	-0.342767
C	0.670292	2.628186	-0.270261
C	-0.511396	3.372138	-0.140782
C	-0.709251	4.852588	-0.012110
O	0.127776	5.736744	0.010001
N	-2.089497	5.028174	0.085059
C	-2.798909	3.812447	0.035206
O	-4.013515	3.710882	0.106004
C	-1.769391	2.763283	-0.110908
H	-0.457220	-2.512690	1.943847
H	-0.744805	-2.114032	4.379408
H	0.872178	-0.582906	5.557319
H	2.711427	0.472803	4.197954
H	2.841551	-0.023734	1.766608
H	-1.893435	-2.508566	-0.485555
H	-4.099655	1.183756	-0.096704
H	4.472631	0.335672	-0.687272
H	5.934223	2.347108	-0.607214
H	2.500509	4.941834	-0.205420
H	-2.536377	5.930869	0.179955
C	1.391776	-1.744458	-2.404105
O	1.402162	-1.798421	-3.562129
C	-4.379095	-1.577034	-0.261683
O	-4.372366	-2.795991	-0.274990
O	-5.496349	-0.831690	-0.219245
C	-6.757671	-1.551461	-0.193102
C	-7.863412	-0.514135	-0.151902
H	-6.811396	-2.184399	-1.084824
H	-6.763999	-2.204486	0.685837
H	-7.777958	0.113805	0.741265
H	-8.837762	-1.015837	-0.131550
H	-7.825155	0.132931	-1.034522
H	4.973577	4.616570	-0.371838

1b

Re	1.912214	-0.036979	-0.478883
C	3.343539	-1.325371	-0.364557
O	4.171725	-2.139491	-0.272805
C	3.145435	1.426703	-0.285871
O	3.870896	2.323468	-0.143333
N	1.512662	-0.124083	1.742285
C	1.600820	-1.282125	2.429223
C	1.300955	-1.379803	3.784345
C	0.895574	-0.233546	4.470704
C	0.809136	0.968128	3.766761
C	1.122443	0.982607	2.410491
N	0.286008	-1.596934	-0.524745
C	0.388071	-2.925273	-0.580171
C	-0.758512	-3.735854	-0.510417
C	-2.030864	-3.221105	-0.383038
C	-2.160067	-1.812903	-0.330932
C	-0.958760	-1.045806	-0.409565
C	-1.020670	0.363125	-0.376061
N	0.077826	1.142152	-0.455548
C	-0.388736	2.452917	-0.395385
C	0.352601	3.640584	-0.433131
C	-0.342233	4.845694	-0.337459
C	-1.747726	4.879896	-0.208330
C	-2.495750	3.704877	-0.171903
C	-1.819456	2.478477	-0.263460
C	-2.233853	1.097063	-0.255630
C	-3.399079	0.325825	-0.167966
C	-4.829732	0.753176	-0.026312
O	-5.286164	1.879721	0.047470
N	-5.562313	-0.432822	0.009611
C	-4.750067	-1.579528	-0.091177
O	-5.160989	-2.729480	-0.079050
C	-3.369397	-1.073484	-0.204621
H	1.919291	-2.150282	1.863652
H	1.386316	-2.339804	4.283301
H	0.654046	-0.276497	5.528892
H	0.499303	1.888698	4.250692
H	1.052980	1.893945	1.829288
H	1.373892	-3.364770	-0.684907
H	-2.900734	-3.866956	-0.328410
H	1.432508	3.619100	-0.543269
H	0.211692	5.780510	-0.368608
H	-3.576866	3.719812	-0.075403
H	-6.569677	-0.469157	0.096669
C	2.072711	0.031795	-2.403034
O	2.146234	0.070103	-3.559057
F	-0.570817	-5.074449	-0.568648
H	-2.252623	5.839624	-0.139897

1c

Re	-1.994803	-0.543310	-0.486447
C	-3.870672	-0.103531	-0.393950
O	-4.990326	0.210969	-0.316614
C	-2.363435	-2.413017	-0.224617
O	-2.560145	-3.544369	-0.040495
N	-1.692635	-0.187579	1.724613
C	-2.327464	0.813064	2.369265
C	-2.114109	1.098414	3.714292
C	-1.208221	0.317882	4.434924
C	-0.552765	-0.721769	3.774233
C	-0.818787	-0.941277	2.425541
N	-1.323788	1.602348	-0.606163
C	-2.046928	2.720231	-0.713336
C	-1.459311	3.999768	-0.685863
C	-0.085705	4.138409	-0.542163
C	0.713293	2.976166	-0.434984
C	0.031685	1.724899	-0.479127
C	0.764804	0.521778	-0.396671
N	0.178050	-0.690789	-0.444811
C	1.218609	-1.612615	-0.341623
C	1.154671	-3.007075	-0.336352
C	2.341809	-3.731141	-0.203626
C	3.587115	-3.072844	-0.078403
C	3.666726	-1.677036	-0.083538
C	2.484473	-0.942377	-0.213869
C	2.180626	0.468198	-0.255932
C	2.829989	1.707441	-0.200762
C	4.287701	2.025246	-0.049832
O	5.228985	1.260749	0.062612
N	4.358724	3.417692	-0.059250
C	3.095458	4.027545	-0.198103
O	2.905910	5.234285	-0.228229
C	2.130283	2.916599	-0.287265

H	-3.020997	1.398257	1.776479
H	-2.650874	1.919357	4.178855
H	-1.018351	0.515640	5.486045
H	0.162231	-1.358045	4.285710
H	-0.317338	-1.728723	1.876258
H	-3.118797	2.593282	-0.827228
H	0.387574	5.114684	-0.514987
H	0.204974	-3.522505	-0.442062
H	2.290839	-4.813850	-0.204108
H	4.628251	-1.183821	0.010811
H	5.222648	3.937605	0.022661
C	-2.096736	-0.750373	-2.403523
O	-2.140236	-0.860929	-3.556939
H	-2.098002	4.872215	-0.780429
O	4.783642	-3.730125	0.055683
C	4.793114	-5.149401	0.038266
H	5.840085	-5.440081	0.144912
H	4.401525	-5.543664	-0.909980
H	4.213968	-5.567595	0.873858

1d

Re	-2.018653	-0.871056	-0.485276
C	-3.944595	-0.797562	-0.404617
O	-5.104309	-0.702769	-0.334367
C	-2.021786	-2.774888	-0.209165
O	-1.998195	-3.921608	-0.016476
N	-1.802832	-0.448034	1.723968
C	-2.613187	0.426351	2.355109
C	-2.464781	0.757189	3.698477
C	-1.437773	0.159869	4.431674
C	-0.599600	-0.748913	3.784673
C	-0.811395	-1.024267	2.436597
N	-1.771229	1.362368	-0.621923
C	-2.694007	2.321529	-0.742400
C	-2.361142	3.689002	-0.724042
C	-1.038997	4.088124	-0.575652
C	-0.033959	3.101305	-0.454710
C	-0.464612	1.741940	-0.490570
C	0.483096	0.702251	-0.393170
N	0.141085	-0.600548	-0.430180
C	1.338985	-1.303135	-0.308374
C	1.557912	-2.683642	-0.292066
C	2.859176	-3.153983	-0.145925
C	3.980362	-2.279965	-0.006487
C	3.755853	-0.889413	-0.043164
C	2.449445	-0.406915	-0.183120
C	1.884162	0.919472	-0.244528
C	2.285208	2.259216	-0.198793
C	3.654810	2.849389	-0.044481
O	4.725555	2.278945	0.078109
N	3.460639	4.228291	-0.065128
C	2.103434	4.586572	-0.216210
O	1.692158	5.736754	-0.257677
C	1.368260	3.313715	-0.300129
H	-3.396671	0.871304	1.752620
H	-3.144663	1.471323	4.151988
H	-1.294695	0.397566	5.481957
H	0.215417	-1.240439	4.306051
H	-0.170410	-1.711021	1.897356
H	-3.721360	1.991510	-0.859373
H	-0.761177	5.137065	-0.555036
H	0.732975	-3.381108	-0.404211
H	3.008322	-4.226841	-0.142429
H	4.561050	-0.171015	0.033469
H	4.209047	4.904037	0.015389
C	-2.066774	-1.108393	-2.400584
O	-2.080839	-1.233685	-3.553377
H	-3.153557	4.423256	-0.829109
N	5.266915	-2.798179	0.178473
C	5.517162	-4.189577	-0.169909
C	6.395261	-1.884323	0.065055
H	4.933271	-4.866269	0.464352
H	6.571815	-4.415742	0.002348
H	5.280669	-4.414588	-1.224221
H	7.319789	-2.432360	0.260935
H	6.322048	-1.083919	0.808887
H	6.466990	-1.417582	-0.932375

1e

Re	0.380016	-2.101796	-0.467175
C	-0.791651	-3.632606	-0.352602
O	-1.525043	-4.531794	-0.259428
C	1.935520	-3.223470	-0.333173
O	2.891304	-3.877525	-0.225515
N	0.307630	-1.750658	1.762793
C	-0.826228	-1.946052	2.467903
C	-0.921300	-1.680346	3.830447
C	0.200986	-1.194778	4.504115
C	1.377564	-0.996218	3.780590
C	1.391176	-1.282518	2.418344
N	-1.287584	-0.586790	-0.435281
C	-2.604963	-0.771601	-0.441624
C	-3.518886	0.302164	-0.328912
C	-3.050516	1.608291	-0.209447
C	-1.658213	1.838125	-0.200987
C	-0.815975	0.694326	-0.319396
C	0.583502	0.850399	-0.324552
N	1.431248	-0.191846	-0.438743
C	2.707938	0.362877	-0.410970
C	3.944015	-0.282077	-0.494604
C	5.106004	0.488709	-0.429331
C	5.042995	1.894977	-0.283431
C	3.814011	2.554710	-0.199874
C	2.642877	1.792660	-0.262099
C	1.237786	2.111569	-0.209782
C	0.393706	3.222343	-0.085196
C	0.729685	4.676386	0.063020
O	1.824860	5.206516	0.109464
N	-0.500899	5.326845	0.145088
C	-1.593572	4.440134	0.067170
O	-2.766843	4.775356	0.121091
C	-0.999645	3.097781	-0.080956
H	-1.676914	-2.323127	1.911823
H	-1.861333	-1.853730	4.344383
H	0.159129	-0.977264	5.567584
H	2.278567	-0.619929	4.254209
H	2.281511	-1.127141	1.821248
H	-2.970802	-1.787626	-0.540406
H	-3.737441	2.442384	-0.122315
H	4.004001	-1.359089	-0.617159
H	6.064460	-0.012688	-0.498830
H	3.778411	3.633393	-0.091106
H	-0.602081	6.328178	0.247867
C	0.411035	-2.220853	-2.394219
O	0.425000	-2.268730	-3.552559
C	-4.970992	-0.040028	-0.333736
O	-5.396025	-1.182225	-0.365855
O	-5.753450	1.051888	-0.294003
C	-7.187728	0.824567	-0.294728
C	-7.855894	2.185560	-0.253034
H	-7.440008	0.208304	0.574561
H	-7.446591	0.259487	-1.196052
H	-7.575991	2.784969	-1.125691
H	-7.569172	2.734797	0.650011
H	-8.944915	2.060925	-0.252510
O	6.150212	2.700654	-0.213327
C	7.437404	2.112312	-0.326406
H	7.564288	1.603048	-1.291973
H	7.629539	1.401112	0.489421
H	8.147566	2.938698	-0.258201

1f

Re	-0.055842	-2.207893	-0.466534
C	-1.426116	-3.565259	-0.369502
O	-2.276068	-4.356543	-0.286744
C	1.332492	-3.530072	-0.323485

O	2.190101	-4.307646	-0.209849
N	-0.100923	-1.856931	1.763829
C	-1.258030	-1.894087	2.456959
C	-1.328866	-1.621894	3.819697
C	-0.156584	-1.300017	4.506232
C	1.043413	-1.265247	3.794855
C	1.030673	-1.546309	2.431480
N	-1.503205	-0.480492	-0.441742
C	-2.833861	-0.482791	-0.459514
C	-3.592542	0.705028	-0.348523
C	-2.949600	1.935146	-0.218554
C	-1.540141	1.972337	-0.198159
C	-0.861048	0.723516	-0.315914
C	0.545132	0.686441	-0.307876
N	1.245387	-0.460275	-0.418255
C	2.584332	-0.083158	-0.370653
C	3.731417	-0.879907	-0.448192
C	4.977318	-0.268692	-0.364806
C	5.132498	1.142693	-0.197104
C	3.974808	1.942726	-0.138611
C	2.715156	1.336208	-0.215839
C	1.367568	1.845783	-0.179168
C	0.683376	3.060835	-0.056928
C	1.215140	4.453827	0.101749
O	2.373673	4.829297	0.159386
N	0.086937	5.266794	0.176961
C	-1.118746	4.538498	0.084451
O	-2.233545	5.036166	0.129938
C	-0.715068	3.129609	-0.065786
H	-2.147195	-2.146802	1.890781
H	-2.289099	-1.663645	4.323753
H	-0.178428	-1.082183	5.570258
H	1.983363	-1.019710	4.278520
H	1.939702	-1.513711	1.843274
H	-3.334369	-1.438836	-0.566238
H	-3.516861	2.854854	-0.132147
H	3.656152	-1.954647	-0.584399
H	5.853370	-0.901987	-0.433879
H	4.019191	3.018690	-0.035781
H	0.122973	6.272118	0.283425
C	-0.021923	-2.323909	-2.393443
O	-0.002314	-2.370085	-3.551938
C	-5.076838	0.565578	-0.366470
O	-5.656122	-0.506553	-0.410393
O	-5.702957	1.754800	-0.324282
C	-7.154174	1.725219	-0.337219
C	-7.630425	3.164537	-0.289927
H	-7.495746	1.143730	0.525516
H	-7.480734	1.206927	-1.244677
H	-8.726333	3.189915	-0.298411
H	-7.263533	3.725545	-1.155988
H	-7.278508	3.663144	0.619555
N	6.406082	1.706674	-0.079009
C	7.560116	0.933618	-0.515963
C	6.523088	3.156614	-0.149321
H	7.493709	0.630588	-1.574932
H	7.683782	0.030330	0.092331
H	8.461957	1.535913	-0.386258
H	6.172010	3.565674	-1.112097
H	5.945502	3.631313	0.651026
H	7.569673	3.437768	-0.011904

1g

Re	-0.878293	-2.240181	-0.482671
C	-2.519635	-3.249997	-0.370650
O	-3.526577	-3.825942	-0.277321
C	0.168769	-3.850722	-0.384469
O	0.821194	-4.808820	-0.298179
N	-0.796594	-1.929569	1.751449
C	-1.915342	-1.701424	2.470819
C	-1.893308	-1.455404	3.840154
C	-0.665944	-1.446850	4.505585
C	0.493531	-1.687616	3.767297
C	0.388147	-1.922088	2.399251
N	-1.895342	-0.229895	-0.421032
C	-3.190916	0.066947	-0.418215
C	-3.663677	1.396429	-0.301983
C	-2.762236	2.450069	-0.188360
C	-1.378539	2.168904	-0.189002
C	-0.997871	0.800724	-0.310777
C	0.364470	0.451221	-0.325259
N	0.789682	-0.829040	-0.444341
C	2.171822	-0.764683	-0.421080
C	3.096106	-1.818560	-0.503272
C	4.446529	-1.507544	-0.433338
C	4.898106	-0.170386	-0.287576
C	3.987200	0.887573	-0.210154
C	2.619625	0.596045	-0.273435
C	1.422165	1.394549	-0.215389
C	1.029585	2.735309	-0.087436
C	1.860930	3.974393	0.056466
O	3.072728	4.080510	0.096392
N	0.941790	5.020752	0.143428
C	-0.393361	4.581245	0.073316
O	-1.372207	5.307743	0.132108
C	-0.315512	3.111806	-0.075518
H	-2.849440	-1.717195	1.920949
H	-2.825623	-1.276286	4.366035
H	-0.615159	-1.258881	5.574254
H	1.473158	-1.693323	4.234179
H	1.267135	-2.101163	1.792117
H	-3.895777	-0.751807	-0.512512
H	-3.107228	3.473884	-0.098955
H	2.761244	-2.843709	-0.626421
H	5.193602	-2.292570	-0.494102
H	4.323748	1.912115	-0.101229
H	1.204189	5.992630	0.244963
C	-0.908066	-2.329989	-2.411924
O	-0.922527	-2.359254	-3.570593
C	-5.143487	1.591539	-0.297408
O	-5.944293	0.673071	-0.324453
O	-5.487194	2.889314	-0.255373
C	-6.909595	3.185732	-0.247462
C	-7.051450	4.695163	-0.204320
H	-7.358196	2.698358	0.624394
H	-7.356742	2.749313	-1.146434
H	-6.583742	5.106715	0.696337
H	-6.582558	5.156819	-1.079609
H	-8.113914	4.964402	-0.197570
C	6.367549	0.054905	-0.221373
O	7.204326	-0.832957	-0.261138
O	6.688954	1.363172	-0.110484
C	8.098959	1.678170	-0.044474
C	8.215187	3.187129	0.072253
H	8.534208	1.160680	0.817427
H	8.586644	1.293359	-0.946679
H	9.271869	3.474842	0.125127
H	7.763333	3.680938	-0.794658
H	7.710745	3.548487	0.974847

1h

Re	-1.756857	-1.050567	-0.475469
C	-3.684017	-1.018603	-0.382183
O	-4.844316	-0.948785	-0.304419
C	-1.722632	-2.960332	-0.251942
O	-1.675762	-4.110714	-0.090557
N	-1.537193	-0.684585	1.742856
C	-2.379265	0.133171	2.408347
C	-2.232610	0.428484	3.760050
C	-1.173888	-0.146091	4.465897
C	-0.303804	-0.997637	3.784130
C	-0.515792	-1.239827	2.429758
N	-1.552488	1.193005	-0.548458
C	-2.501128	2.128505	-0.622763
C	-2.167113	3.491919	-0.561946
C	-0.869613	3.938758	-0.425553
C	0.150687	2.961313	-0.353923
C	-0.253459	1.594010	-0.423103

C	0.717083	0.570039	-0.368396
N	0.398388	-0.736414	-0.436559
C	1.611665	-1.421483	-0.358594
C	1.841733	-2.797525	-0.381197
C	3.156380	-3.258155	-0.272148
C	4.236392	-2.355145	-0.142806
C	4.020309	-0.973384	-0.119311
C	2.708981	-0.502528	-0.225860
C	2.114449	0.813753	-0.237283
C	2.487337	2.159290	-0.160142
C	3.846932	2.775774	-0.010629
O	4.928823	2.224303	0.079196
N	3.621865	4.151416	0.010488
C	2.257033	4.483905	-0.107552
O	1.814631	5.622694	-0.109578
C	1.547815	3.197239	-0.215934
H	-3.188831	0.560581	1.827986
H	-2.939022	1.097303	4.241198
H	-1.031500	0.064795	5.521930
H	0.535734	-1.470067	4.283775
H	0.148038	-1.882754	1.864761
H	-3.532220	1.812470	-0.735769
H	-0.636890	4.997245	-0.378417
H	1.020584	-3.499341	-0.490518
H	3.334176	-4.327096	-0.294621
H	4.857590	-0.290603	-0.022196
H	4.357519	4.840319	0.098923
C	-1.812638	-1.238260	-2.397368
O	-1.830649	-1.334275	-3.552327
F	-3.185226	4.380119	-0.638739
O	5.545492	-2.747146	-0.031997
C	5.854869	-4.131846	-0.083677
H	6.940914	-4.196197	0.007096
H	5.542752	-4.578860	-1.037913
H	5.388491	-4.681025	0.746530

1i

Re	1.749358	-0.113260	-0.043315
C	3.195007	-1.344722	-0.157643
O	4.047216	-2.150836	-0.246521
C	2.928564	1.344782	0.264645
O	3.631193	2.264187	0.455860
N	1.361751	-0.465168	2.119013
C	1.533699	-1.681705	2.678321
C	1.239831	-1.949197	4.012058
C	0.750918	-0.919333	4.818062
C	0.576671	0.342147	4.247229
C	0.889426	0.528177	2.903495
N	0.163538	-1.697045	-0.315980
C	0.298522	-3.012508	-0.499398
C	-0.809212	-3.872085	-0.641400
C	-2.100546	-3.366525	-0.598471
C	-2.284318	-1.976091	-0.407785
C	-1.104971	-1.187425	-0.265977
C	-1.211954	0.207964	-0.080147
N	-0.133527	1.008513	0.050133
C	-0.640380	2.295454	0.178420
C	0.071254	3.492590	0.336579
C	-0.659445	4.674413	0.449470
C	-2.071553	4.677506	0.406226
C	-2.789753	3.493945	0.248089
C	-2.077781	2.289441	0.132242
C	-2.452150	0.907650	-0.040686
C	-3.598556	0.114266	-0.177407
C	-5.045416	0.504592	-0.176622
O	-5.539845	1.611487	-0.053729
N	-5.744335	-0.689322	-0.350052
C	-4.893243	-1.807590	-0.464591
O	-5.276030	-2.956950	-0.624732
C	-3.523689	-1.272923	-0.350640
H	1.922338	-2.453351	2.024139
H	1.398215	-2.949184	4.403551
H	0.514555	-1.095191	5.863689
H	0.201395	1.179974	4.826308
H	0.759282	1.490965	2.425120
H	1.315334	-3.389922	-0.544636
H	-2.967943	-4.009010	-0.710478
H	1.156929	3.491271	0.367328
H	-0.129698	5.615932	0.572097
H	-3.874801	3.485847	0.213060
H	-6.753179	-0.750142	-0.392640
P	1.818685	0.272568	-2.391444
C	3.470959	0.669359	-3.101413
C	1.258681	-1.133340	-3.450635
C	0.763385	1.658277	-2.998050
H	4.159190	-0.155541	-2.891130
H	3.415034	0.832051	-4.183926
H	3.862128	1.570047	-2.617779
H	1.891738	-2.006242	-3.261078
H	1.311335	-0.874299	-4.514262
H	0.227196	-1.395759	-3.195664
H	1.052723	2.585229	-2.494038
H	0.861014	1.784348	-4.082332
H	-0.282357	1.457201	-2.747103
H	-0.634118	-4.933151	-0.789571
H	-2.604443	5.620268	0.496938

1j

Re	-0.514110	1.612999	-0.068549
C	-1.507981	2.687080	-1.282552
O	-2.090894	3.321832	-2.084185
C	-1.385238	2.380627	1.435051
O	-1.905325	2.831702	2.385553
N	1.234658	2.987380	0.024207
C	1.626019	3.707806	-1.048263
C	2.748072	4.531068	-1.037768
C	3.506478	4.628355	0.130449
C	3.104177	3.889810	1.244229
C	1.972111	3.084929	1.151985
N	0.692497	0.610883	-1.695197
C	0.641215	0.782224	-3.018212
C	1.466192	0.061895	-3.904822
C	2.371380	-0.871319	-3.420551
C	2.455506	-1.080670	-2.023177
C	1.589381	-0.297950	-1.204416
C	1.613778	-0.459843	0.198051
N	0.810820	0.241370	1.025632
C	1.109306	-0.212152	2.304461
C	0.547545	0.200960	3.520277
C	1.008174	-0.402881	4.689032
C	2.008255	-1.400422	4.660919
C	2.574181	-1.819130	3.458445
C	2.127922	-1.226271	2.266446
C	2.458307	-1.388554	0.872109
C	3.302501	-2.145863	0.050029
C	4.306882	-3.198056	0.410029
O	4.608039	-3.624716	1.511086
N	4.851376	-3.611853	-0.804885
C	4.297194	-2.933559	-1.910106
O	4.615513	-3.128515	-3.073688
C	3.307813	-1.999275	-1.342374
H	1.008072	3.613429	-1.933596
H	3.012297	5.084992	-1.933175
H	4.386003	5.264648	0.171869
H	3.654659	3.927514	2.178976
H	1.635077	2.491920	1.993173
H	-0.086067	1.501431	-3.381819
H	3.010565	-1.443450	-4.085216
H	-0.224872	0.964272	3.541843
H	0.585912	-0.100703	5.644286
H	3.343043	-2.584947	3.426312
H	5.566103	-4.322795	-0.888605
P	-2.128437	-0.137783	-0.136154
C	-3.931198	0.397915	-0.102065
N	-4.933099	-0.611290	-0.342155
C	-5.422031	-1.413699	0.766718
C	-4.389797	-1.781164	1.867257
N	-3.103816	-2.296068	1.444091

C	-2.021551	-1.355035	1.299431
C	-5.029786	-1.149700	-1.683965
C	-4.239626	-2.446910	-2.007856
N	-2.845809	-2.484181	-1.598076
C	-2.417556	-3.527424	-0.686280
C	-3.053452	-3.558841	0.730067
C	-2.059762	-1.275323	-1.639901
H	-4.103996	0.887091	0.863871
H	-4.009739	1.191542	-0.856635
H	-6.255241	-0.901372	1.281043
H	-5.844284	-2.334618	0.348190
H	-4.195433	-0.895880	2.484258
H	-4.880350	-2.508865	2.533185
H	-1.938687	-0.735829	2.201148
H	-1.064640	-1.881881	1.219755
H	-4.713956	-0.357425	-2.373264
H	-6.084887	-1.353969	-1.925920
H	-4.744636	-3.300661	-1.541760
H	-4.329264	-2.598094	-3.099311
H	-1.328898	-3.442159	-0.586540
H	-2.601002	-4.513801	-1.140792
H	-4.078563	-3.939908	0.655880
H	-2.486884	-4.312877	1.306360
H	-2.330850	-0.677664	-2.518634
H	-1.000466	-1.532061	-1.766034
H	1.374883	0.243893	-4.971146
H	2.339421	-1.847959	5.594222

1k

Re	-1.424203	-1.439960	-0.055252
C	-1.788986	-2.869262	-1.261845
O	-2.001456	-3.710269	-2.054557
C	-1.671115	-2.567777	1.457833
O	-1.821196	-3.235957	2.408420
N	-3.503706	-0.670594	-0.024524
C	-4.299629	-0.738908	-1.112853
C	-5.585279	-0.207382	-1.143015
C	-6.085854	0.420751	-0.001088
C	-5.269415	0.490173	1.128584
C	-3.992384	-0.061332	1.078027
N	-1.129893	0.088347	-1.697553
C	-1.206932	-0.060903	-3.021888
C	-0.892453	0.979345	-3.918430
C	-0.467868	2.210923	-3.441978
C	-0.374761	2.407927	-2.044076
C	-0.739578	1.307608	-1.214740
C	-0.660143	1.430412	0.189296
N	-0.961922	0.413880	1.029459
C	-0.682565	0.891295	2.303621
C	-0.802211	0.215634	3.526270
C	-0.431252	0.895042	4.685503
C	0.051333	2.222320	4.642009
C	0.173836	2.904423	3.433412
C	-0.192392	2.242009	2.250390
C	-0.179887	2.593481	0.852100
C	0.179600	3.662606	0.018372
C	0.726287	5.013724	0.366941
O	0.973727	5.477201	1.466135
N	0.903970	5.660673	-0.854847
C	0.533453	4.860462	-1.954077
O	0.597293	5.210121	-3.121801
C	0.075350	3.581963	-1.372455
H	-3.880313	-1.240765	-1.976940
H	-6.177057	-0.292728	-2.048941
H	-7.086691	0.842962	0.008271
H	-5.606974	0.965709	2.043990
H	-3.327174	-0.017118	1.931283
H	-1.506223	-1.041368	-3.379117
H	-0.196608	3.018843	-4.113668
H	-1.161462	-0.808916	3.560593
H	-0.511152	0.390046	5.644966
H	0.546851	3.922984	3.388606
H	1.268931	6.599713	-0.947128
P	0.897380	-1.858747	-0.099170
C	1.754849	-1.543477	-1.727825
N	3.198032	-1.753658	-1.694604
C	4.015009	-0.638769	-1.755985
O	3.537663	0.493824	-1.838758
C	5.517682	-0.856960	-1.751460
C	3.620944	-3.068467	-1.180791
N	3.128836	-3.397221	0.153307
C	3.678991	-2.600227	1.240930
N	3.295812	-1.175910	1.219666
C	4.260541	-0.213906	1.450106
O	5.448090	-0.523350	1.572037
C	3.814349	1.236674	1.512010
C	1.871253	-0.874304	1.157472
C	1.667492	-3.519795	0.219527
H	1.555883	-0.524631	-2.058906
H	1.296925	-2.238021	-2.446305
H	5.825404	-1.656271	-2.434074
H	5.981849	0.080729	-2.061435
H	5.873842	-1.090890	-0.742219
H	3.244796	-3.839067	-1.865267
H	4.708471	-3.117834	-1.171751
H	4.767435	-2.637606	1.215890
H	3.335881	-3.050860	2.182441
H	4.690589	1.836179	1.762616
H	3.035826	1.404606	2.264885
H	3.431043	1.560209	0.537332
H	1.382871	-1.064164	2.124491
H	1.714529	0.176516	0.923822
H	1.368814	-3.893458	1.205235
H	1.313826	-4.239670	-0.527581
H	-0.969258	0.793813	-4.985124
H	0.333227	2.716393	5.567852

1l

Re	1.380032	-1.399354	-0.029563
C	0.970069	-3.259073	-0.001844
O	0.672863	-4.395485	0.003819
C	3.243779	-1.752617	0.067613
O	4.400709	-1.937225	0.125604
N	1.143067	-1.122591	2.164358
C	0.218046	-1.812533	2.865542
C	0.001469	-1.617980	4.226457
C	0.773228	-0.674870	4.907753
C	1.734811	0.037447	4.189547
C	1.887559	-0.211564	2.828454
N	-0.777381	-0.731231	-0.073285
C	-1.889266	-1.460742	-0.081814
C	-3.179246	-0.877997	-0.101023
C	-3.320194	0.507023	-0.117356
C	-2.159781	1.311378	-0.107898
C	-0.905778	0.633717	-0.078876
C	0.291766	1.375405	-0.073767
N	1.508897	0.792224	-0.056987
C	2.420289	1.837984	-0.092337
C	3.820640	1.764191	-0.096321
C	4.534882	2.959870	-0.132400
C	3.880929	4.212832	-0.165175
C	2.491115	4.299726	-0.162703
C	1.746407	3.109165	-0.126349
C	0.339907	2.800047	-0.115648
C	-0.902700	3.447512	-0.140306
C	-1.226162	4.910385	-0.179853
O	-0.465834	5.862132	-0.198101
N	-2.618963	4.974511	-0.192122
C	-3.224099	3.701574	-0.166872
O	-4.429685	3.505545	-0.173799
C	-2.107952	2.736902	-0.133138
H	-0.356095	-2.541763	2.305847
H	-0.758341	-2.203500	4.734515
H	0.630631	-0.501996	5.970728
H	2.364228	0.781351	4.667681
H	2.617897	0.327244	2.237474
H	-1.780495	-2.539729	-0.083772
H	-4.300217	0.969669	-0.138318

H	4.324163	0.802299	-0.074019
H	5.621645	2.928413	-0.137197
H	1.979185	5.256687	-0.188475
H	-3.142407	5.839755	-0.219286
P	1.461503	-1.404220	-2.408565
C	2.455516	-2.746170	-3.184360
C	-0.168046	-1.570840	-3.262261
C	2.150311	0.121685	-3.183092
H	2.050867	-3.715728	-2.876974
H	2.442839	-2.674981	-4.278066
H	3.488045	-2.679693	-2.827087
H	-0.819999	-0.740805	-2.972344
H	-0.646875	-2.506363	-2.955627
H	-0.046889	-1.567782	-4.351486
H	1.549243	0.985794	-2.884582
H	2.158011	0.042200	-4.276150
H	3.169905	0.285495	-2.821503
C	-4.344602	-1.808641	-0.106486
O	-4.242081	-3.023153	-0.075465
O	-5.520513	-1.157019	-0.148229
C	-6.717649	-1.977630	-0.156042
C	-7.905318	-1.035388	-0.206087
H	-6.716064	-2.599891	0.745005
H	-6.675234	-2.642449	-1.025074
H	-7.875339	-0.416433	-1.109032
H	-8.835495	-1.615310	-0.213454
H	-7.916157	-0.373349	0.666229
H	4.475402	5.122052	-0.193868

1m

Re	0.392977	-1.956085	-0.022862
C	-0.738980	-3.488155	-0.005441
O	-1.472694	-4.405510	-0.006358
C	1.952387	-3.033914	0.089624
O	2.936999	-3.668702	0.156386
N	0.262756	-1.605330	2.169802
C	-0.868410	-1.867943	2.858929
C	-1.004925	-1.599727	4.217712
C	0.070875	-1.040470	4.909836
C	1.244645	-0.771988	4.204155
C	1.300922	-1.064544	2.844303
N	-1.303751	-0.465587	-0.082315
C	-2.617144	-0.677958	-0.104334
C	-3.555298	0.380734	-0.127806
C	-3.117403	1.703148	-0.133373
C	-1.730197	1.963319	-0.109887
C	-0.863498	0.831979	-0.078596
C	0.533656	1.019210	-0.060642
N	1.403353	-0.009578	-0.041346
C	2.665538	0.573228	-0.061267
C	3.913593	-0.054531	-0.057478
C	5.061038	0.740439	-0.078621
C	4.970616	2.152762	-0.104525
C	3.729490	2.795202	-0.109395
C	2.572476	2.009357	-0.088171
C	1.160134	2.301705	-0.089449
C	0.291747	3.399145	-0.115942
C	0.595042	4.867132	-0.143878
O	1.678394	5.424203	-0.148531
N	-0.649597	5.494385	-0.163946
C	-1.722842	4.579078	-0.154005
O	-2.903301	4.894502	-0.169930
C	-1.099750	3.243867	-0.122566
H	-1.679331	-2.309283	2.291234
H	-1.940949	-1.832443	4.715681
H	-0.003202	-0.822096	5.971426
H	2.112608	-0.338645	4.691024
H	2.191567	-0.861674	2.262445
H	-2.958116	-1.707348	-0.113890
H	-3.822479	2.526069	-0.156732
H	3.990529	-1.137606	-0.040704
H	6.029301	0.253093	-0.076586
H	3.674430	3.878431	-0.129982
H	-0.773775	6.498066	-0.186098

P	0.491389	-1.993606	-2.400681
C	-1.035474	-1.427251	-3.272269
C	0.807167	-3.634548	-3.174375
C	1.789114	-0.923675	-3.158088
H	-1.259935	-0.395558	-2.983630
H	-1.880381	-2.057405	-2.976488
H	-0.912097	-1.476432	-4.360135
H	0.015847	-4.329867	-2.876773
H	0.839906	-3.564223	-4.267742
H	1.760578	-4.026424	-2.805992
H	1.625542	0.115830	-2.858931
H	2.772330	-1.225907	-2.785014
H	1.774432	-0.996443	-4.251573
C	-4.999039	0.008191	-0.149690
O	-5.403188	-1.142063	-0.130307
O	-5.805286	1.084192	-0.192144
C	-7.232943	0.825433	-0.216099
C	-7.930484	2.171569	-0.265057
H	-7.495545	0.251539	0.678799
H	-7.457677	0.206828	-1.091248
H	-7.640466	2.729517	-1.161665
H	-7.678161	2.774733	0.613510
H	-9.016438	2.023378	-0.284400
O	6.063319	2.982473	-0.126102
C	7.361502	2.409146	-0.131876
H	7.543521	1.811787	0.772780
H	7.522289	1.782440	-1.020505
H	8.057024	3.250504	-0.153752

1n

Re	0.927218	0.011144	1.586861
C	0.957854	-1.520175	2.717221
O	0.933181	-2.479981	3.394951
C	2.601047	0.610958	2.252988
O	3.634800	1.010905	2.639170
N	-0.281976	1.250547	2.984287
C	-1.240148	0.703524	3.762762
C	-2.053165	1.458286	4.603154
C	-1.874621	2.842009	4.655921
C	-0.882304	3.412656	3.857315
C	-0.113356	2.589831	3.039069
N	-1.092931	-0.463766	0.688380
C	-1.904793	-1.489434	0.928158
C	-3.141244	-1.651192	0.258564
C	-3.546434	-0.721299	-0.695054
C	-2.709923	0.382366	-0.970633
C	-1.489678	0.465690	-0.237990
C	-0.604268	1.536548	-0.470766
N	0.572209	1.663674	0.178828
C	1.158470	2.810030	-0.339049
C	2.394175	3.377996	0.002651
C	2.780566	4.541322	-0.659149
C	1.962477	5.139153	-1.645057
C	0.733362	4.585108	-1.993464
C	0.319535	3.411621	-1.341344
C	-0.843895	2.567088	-1.427417
C	-2.049164	2.468501	-2.135257
C	-2.618158	3.359310	-3.197644
O	-2.147302	4.375242	-3.677969
N	-3.837244	2.778387	-3.544480
C	-4.109275	1.603245	-2.814459
O	-5.113983	0.921213	-2.946396
C	-2.952107	1.421941	-1.917211
H	-1.343685	-0.373717	3.702494
H	-2.806988	0.959884	5.204555
H	-2.490819	3.458486	5.304318
H	-0.698204	4.482399	3.859062
H	0.660252	2.997726	2.400131
H	-1.580588	-2.224846	1.656235
H	-4.485718	-0.834735	-1.223964
H	3.025540	2.916606	0.756253
H	3.735600	4.999047	-0.413259
H	0.097905	5.036067	-2.749369
H	-4.461704	3.156049	-4.245054

P	2.019904	-1.156003	-0.179749
C	3.434200	-2.287512	0.326540
N	3.983160	-3.161117	-0.680864
C	5.034200	-2.672206	-1.557225
C	4.951699	-1.179027	-1.976401
N	3.676790	-0.676369	-2.446390
C	2.810562	-0.045590	-1.481841
C	3.154660	-4.260203	-1.134382
C	2.278133	-4.038551	-2.397023
N	1.490298	-2.817959	-2.439798
C	1.706378	-1.888442	-3.531364
C	3.108217	-1.233022	-3.660612
C	0.929362	-2.291059	-1.219927
H	4.219570	-1.651635	0.752104
H	3.042809	-2.879507	1.164101
H	6.026422	-2.808448	-1.089979
H	5.034127	-3.298843	-2.456591
H	5.251883	-0.552983	-1.127779
H	5.719939	-1.023175	-2.750430
H	3.366247	0.713984	-0.918028
H	2.006534	0.498800	-1.988942
H	2.507733	-4.544173	-0.295853
H	3.785197	-5.140269	-1.337585
H	1.625324	-4.926743	-2.479236
H	2.918853	-4.050255	-3.286130
H	0.951288	-1.098773	-3.437288
H	1.498785	-2.389415	-4.490133
H	3.816640	-1.972943	-4.050725
H	3.015092	-0.456324	-4.441517
H	0.591881	-3.109371	-0.572494
H	0.032678	-1.703656	-1.454306
C	-3.956572	-2.846991	0.617940
O	-3.627576	-3.663169	1.461817
O	-5.095840	-2.922887	-0.093256
C	-5.960002	-4.055287	0.183878
C	-7.165500	-3.931503	-0.728502
H	-6.234245	-4.029847	1.243729
H	-5.393209	-4.974798	0.004218
H	-7.705801	-2.998183	-0.537860
H	-6.862219	-3.946167	-1.780706
H	-7.848646	-4.770481	-0.552299
H	2.301597	6.045983	-2.138589

1o

Re	0.400946	-0.839782	1.617257
C	-0.444787	-2.325130	2.456118
O	-1.009159	-3.229188	2.951401
C	2.063947	-1.241191	2.440066
O	3.110293	-1.458901	2.926329
N	-0.226942	0.628561	3.167403
C	-1.413067	0.518194	3.803278
C	-1.862253	1.452181	4.732169
C	-1.056338	2.552211	5.031683
C	0.173011	2.669091	4.381229
C	0.548904	1.694899	3.460632
N	-1.473735	-0.121761	0.576519
C	-2.701960	-0.633229	0.598273
C	-3.774600	-0.058559	-0.122967
C	-3.566098	1.080690	-0.896896
C	-2.275148	1.650189	-0.939659
C	-1.261539	1.006784	-0.171042
C	0.052578	1.517774	-0.175867
N	1.056532	0.947087	0.518475
C	2.181946	1.714760	0.239157
C	3.487173	1.546513	0.707802
C	4.469158	2.444398	0.286034
C	4.157318	3.505457	-0.597405
C	2.856144	3.687720	-1.073545
C	1.863449	2.793848	-0.658115
C	0.453537	2.660959	-0.931716
C	-0.556868	3.279234	-1.677090
C	-0.496652	4.482240	-2.569697
O	0.459321	5.187899	-2.838877
N	-1.795060	4.642407	-3.050488
C	-2.684269	3.663251	-2.559495
O	-3.871075	3.600131	-2.843736
C	-1.872819	2.800649	-1.682600
H	-2.007743	-0.352492	3.552224
H	-2.826571	1.308270	5.209362
H	-1.377200	3.297060	5.754469
H	0.840478	3.502746	4.575575
H	1.491154	1.756681	2.930149
H	-2.860136	-1.530735	1.186005
H	-4.374807	1.529089	-1.462373
H	3.734371	0.731521	1.381555
H	5.482445	2.311737	0.647510
H	2.630619	4.505324	-1.749944
H	-2.073551	5.379600	-3.684743
P	1.008362	-2.131982	-0.289710
C	1.543962	-3.904095	0.041422
N	1.725003	-4.770284	-1.097361
C	3.003347	-4.788589	-1.787976
C	3.764036	-3.437821	-1.879959
N	3.021041	-2.268277	-2.303075
C	2.453225	-1.430400	-1.276163
C	0.537756	-5.175380	-1.822989
C	0.115275	-4.331721	-3.056619
N	0.080065	-2.889556	-2.880751
C	0.913038	-2.063943	-3.733235
C	2.452475	-2.244866	-3.638929
C	-0.323490	-2.338013	-1.610581
H	2.463135	-3.857137	0.637427
H	0.776414	-4.317284	0.708659
H	3.696570	-5.502313	-1.306793
H	2.827964	-5.171787	-2.799873
H	4.199475	-3.206078	-0.900674
H	4.617811	-3.599682	-2.557155
H	3.218487	-1.180173	-0.530777
H	2.133933	-0.471559	-1.698277
H	-0.289379	-5.195311	-1.103454
H	0.654135	-6.212937	-2.174202
H	0.794882	-4.547046	-3.889112
H	-0.875955	-4.711235	-3.365321
H	0.663158	-1.018828	-3.514711
H	0.636958	-2.224397	-4.787429
H	2.736041	-3.179041	-4.137379
H	2.896405	-1.429921	-4.239256
H	-1.131349	-2.938584	-1.175551
H	-0.743008	-1.335808	-1.764258
C	-5.101818	-0.729588	-0.015296
O	-5.308653	-1.719115	0.666519
O	-6.045963	-0.119557	-0.754468
C	-7.375542	-0.699198	-0.716182
C	-8.257697	0.146139	-1.615256
H	-7.721258	-0.703837	0.322874
H	-7.309607	-1.738687	-1.054127
H	-8.295325	1.182496	-1.263235
H	-9.276888	-0.257486	-1.616629
H	-7.882407	0.144461	-2.644083
O	5.081269	4.415922	-1.045083
C	6.428906	4.294228	-0.617073
H	6.867480	3.336594	-0.931468
H	6.517080	4.394435	0.474052
H	6.967771	5.112778	-1.098466

1p

Re	-1.735861	-0.759724	-0.024000
C	-3.621736	-0.500517	-0.042258
O	-4.776442	-0.280364	-0.069273
C	-1.925576	-2.639322	0.178629
O	-2.007371	-3.802577	0.302576
N	-1.510544	-0.380372	2.157582
C	-2.301985	0.500058	2.806755
C	-2.143731	0.808370	4.154556
C	-1.129350	0.179588	4.879008
C	-0.312253	-0.736683	4.215435
C	-0.531054	-0.986763	2.863552
N	-1.261235	1.442112	-0.181924

C	-2.095869	2.480767	-0.260084
C	-1.601215	3.793677	-0.332642
C	-0.254008	4.088200	-0.333964
C	0.647634	2.999931	-0.253368
C	0.081504	1.692253	-0.172167
C	0.928341	0.563383	-0.094033
N	0.454048	-0.693575	-0.023497
C	1.579864	-1.514542	0.003265
C	1.638713	-2.907335	0.068488
C	2.892999	-3.523583	0.085940
C	4.080712	-2.758144	0.038070
C	4.036330	-1.361419	-0.027816
C	2.787129	-0.735135	-0.046009
C	2.352811	0.641502	-0.111743
C	2.884929	1.931427	-0.188518
C	4.314460	2.383765	-0.220595
O	5.329320	1.710814	-0.185092
N	4.253887	3.773805	-0.301486
C	2.930753	4.262893	-0.327080
O	2.628360	5.445055	-0.397837
C	2.070292	3.071195	-0.253077
H	-3.083992	0.961362	2.215048
H	-2.811167	1.527085	4.619565
H	-0.981579	0.396607	5.933116
H	0.490872	-1.256025	4.728666
H	0.089233	-1.684004	2.314115
H	-3.162276	2.284483	-0.277675
H	0.103536	5.110297	-0.396910
H	0.730204	-3.501384	0.103034
H	2.938541	-4.605447	0.135519
H	4.955723	-0.786740	-0.064547
H	5.069315	4.371231	-0.340980
P	-1.692249	-0.979653	-2.395313
C	-2.896617	-2.167935	-3.122070
C	-2.034201	0.566103	-3.346288
C	-0.083298	-1.527383	-3.111130
H	-3.911522	-1.862893	-2.847817
H	-2.810346	-2.210343	-4.213898
H	-2.715893	-3.163180	-2.703767
H	-3.028446	0.942437	-3.084763
H	-1.990303	0.385409	-4.426321
H	-1.296867	1.330194	-3.080869
H	0.198590	-2.492531	-2.679919
H	-0.145751	-1.616787	-4.201619
H	0.696699	-0.806583	-2.848409
F	-2.512259	4.793468	-0.409154
O	5.338755	-3.305723	0.051931
C	5.472127	-4.717321	0.111831
H	5.012602	-5.203663	-0.760325
H	5.030102	-5.126144	1.031559
H	6.546089	-4.914067	0.109908

1q

Re	-1.735861	-0.759724	-0.024000
C	-3.621736	-0.500517	-0.042258
O	-4.776442	-0.280364	-0.069273
C	-1.925576	-2.639322	0.178629
O	-2.007371	-3.802577	0.302576
N	-1.510544	-0.380372	2.157582
C	-2.301985	0.500058	2.806755
C	-2.143731	0.808370	4.154556
C	-1.129350	0.179588	4.879008
C	-0.312253	-0.736683	4.215435
C	-0.531054	-0.986763	2.863552
N	-1.261235	1.442112	-0.181924
C	-2.095869	2.480767	-0.260084
C	-1.601215	3.793677	-0.332642
C	-0.254008	4.088200	-0.333964
C	0.647634	2.999931	-0.253368
C	0.081504	1.692253	-0.172167
C	0.928341	0.563383	-0.094033
N	0.454048	-0.693575	-0.023497
C	1.579864	-1.514542	0.003265
C	1.638713	-2.907335	0.068488
C	2.892999	-3.523583	0.085940
C	4.080712	-2.758144	0.038070
C	4.036330	-1.361419	-0.027816
C	2.787129	-0.735135	-0.046009
C	2.352811	0.641502	-0.111743
C	2.884929	1.931427	-0.188518
C	4.314460	2.383765	-0.220595
O	5.329320	1.710814	-0.185092
N	4.253887	3.773805	-0.301486
C	2.930753	4.262893	-0.327080
O	2.628360	5.445055	-0.397837
C	2.070292	3.071195	-0.253077
H	-3.083992	0.961362	2.215048
H	-2.811167	1.527085	4.619565
H	-0.981579	0.396607	5.933116
H	0.490872	-1.256025	4.728666
H	0.089233	-1.684004	2.314115
H	-3.162276	2.284483	-0.277675
H	0.103536	5.110297	-0.396910
H	0.730204	-3.501384	0.103034
H	2.938541	-4.605447	0.135519
H	4.955723	-0.786740	-0.064547
H	5.069315	4.371231	-0.340980
P	-1.692249	-0.979653	-2.395313
C	-2.896617	-2.167935	-3.122070
C	-2.034201	0.566103	-3.346288
C	-0.083298	-1.527383	-3.111130
H	-3.911522	-1.862893	-2.847817
H	-2.810346	-2.210343	-4.213898
H	-2.715893	-3.163180	-2.703767
H	-3.028446	0.942437	-3.084763
H	-1.990303	0.385409	-4.426321
H	-1.296867	1.330194	-3.080869
H	0.198590	-2.492531	-2.679919
H	-0.145751	-1.616787	-4.201619
H	0.696699	-0.806583	-2.848409
F	-2.512259	4.793468	-0.409154
O	5.338755	-3.305723	0.051931
C	5.472127	-4.717321	0.111831
H	5.012602	-5.203663	-0.760325
H	6.546089	-4.914067	0.109908
H	5.030102	-5.126144	1.031559

1r

Re	-0.532902	1.607092	0.071638
C	-1.455976	2.744929	-1.140869
O	-1.989874	3.421733	-1.941345
C	-1.467575	2.320367	1.563990
O	-2.029698	2.735616	2.506263
N	1.222336	2.958150	0.301731
C	1.661619	3.727939	-0.716794
C	2.789633	4.537369	-0.621047
C	3.502242	4.567527	0.579218
C	3.049770	3.778613	1.637851
C	1.915413	2.991339	1.461019
N	0.739209	0.663452	-1.538477
C	0.745839	0.900213	-2.851063
C	1.620944	0.203816	-3.702425
C	2.505045	-0.751445	-3.249125
C	2.514747	-1.016659	-1.858313
C	1.610078	-0.268166	-1.046718
C	1.565983	-0.493205	0.346794
N	0.725491	0.172362	1.164340
C	0.962614	-0.338570	2.436344
C	0.345024	0.023537	3.640444
C	0.749023	-0.632513	4.802497
C	1.747362	-1.631090	4.777664
C	2.369175	-1.998786	3.585640
C	1.980514	-1.352659	2.401757
C	2.377325	-1.453882	1.018121
C	3.257856	-2.174437	0.204150
C	4.243794	-3.245397	0.562465
O	4.491157	-3.721884	1.656295
N	4.844657	-3.604890	-0.642905

C	4.344403	-2.875717	-1.741558
O	4.715941	-3.016981	-2.896902
C	3.330942	-1.965852	-1.179869
H	1.077838	3.686433	-1.628999
H	3.093511	5.132757	-1.476326
H	4.385037	5.191362	0.686890
H	3.563120	3.763753	2.594108
H	1.540232	2.360608	2.257434
H	0.049167	1.631658	-3.245910
H	3.165097	-1.281834	-3.926955
H	-0.426212	0.788005	3.659011
H	0.282539	-0.370700	5.748986
H	3.137937	-2.764797	3.556272
H	5.561743	-4.313792	-0.724876
P	-2.160797	-0.123784	-0.134113
C	-3.950101	0.427874	-0.311376
N	-4.928733	-0.579825	-0.636437
C	-5.546383	-1.349524	0.430671
C	-4.646692	-1.696326	1.648135
N	-3.326743	-2.231835	1.384162
C	-2.225740	-1.303148	1.334723
C	-4.881941	-1.151276	-1.967402
C	-4.075419	-2.462982	-2.169234
N	-2.736003	-2.500857	-1.606052
C	-2.422079	-3.523311	-0.626504
C	-3.210434	-3.513241	0.711454
C	-1.937193	-1.300411	-1.591953
H	-3.938000	1.203936	-1.087750
H	-4.222717	0.940701	0.618665
H	-6.425921	-0.817465	0.836169
H	-5.929461	-2.277481	-0.009331
H	-4.513014	-0.796862	2.260802
H	-5.215536	-2.402965	2.273028
H	-2.236793	-0.659206	2.223040
H	-1.271374	-1.838568	1.374738
H	-4.482807	-0.379407	-2.636308
H	-5.905827	-1.352521	-2.320145
H	-4.638268	-3.299918	-1.740551
H	-4.044813	-2.641978	-3.259521
H	-1.350169	-3.444210	-0.409398
H	-2.564827	-4.519540	-1.073863
H	-4.224840	-3.888906	0.534462
H	-2.718201	-4.255731	1.365509
H	-0.873137	-1.569114	-1.591492
H	-2.101953	-0.724683	-2.510691
F	1.563669	0.501368	-5.022679
H	2.033609	-2.120284	5.704857

1s

Re	-0.809188	-0.976482	-1.281752
C	-2.129024	-2.326089	-1.517973
O	-2.921137	-3.191013	-1.609911
C	-1.419536	-0.008454	-2.797494
O	-1.770759	0.618734	-3.725095
N	0.782045	-2.068427	-2.393571
C	0.855499	-3.416563	-2.380384
C	1.873281	-4.124833	-3.012245
C	2.863860	-3.420365	-3.699066
C	2.790141	-2.026888	-3.721649
C	1.742923	-1.391684	-3.060003
N	0.130501	-1.980415	0.511666
C	-0.209399	-3.124060	1.109998
C	0.501332	-3.585370	2.230361
C	1.566439	-2.900748	2.776657
C	1.943516	-1.686082	2.155135
C	1.189066	-1.278652	1.014132
C	1.511393	-0.071492	0.353117
N	0.830251	0.371459	-0.719404
C	1.423629	1.585581	-1.060999
C	1.090833	2.452255	-2.102854
C	1.828598	3.629106	-2.255206
C	2.890941	3.940912	-1.375888
C	3.236395	3.080398	-0.328436
C	2.504455	1.900669	-0.167132
C	2.560485	0.801644	0.769566
C	3.286137	0.382268	1.887633
C	4.437935	1.037277	2.589932
O	4.993002	2.089981	2.328896
N	4.760928	0.177147	3.637787
C	3.932877	-0.964621	3.683248
O	4.025331	-1.856566	4.513863
C	2.997198	-0.815843	2.557235
H	0.064582	-3.929771	-1.845939
H	1.878311	-5.209257	-2.963974
H	3.670156	-3.943996	-4.204833
H	3.531790	-1.428028	-4.240816
H	1.658893	-0.312019	-3.048591
H	-1.056480	-3.677357	0.719590
H	2.093891	-3.273057	3.648223
H	0.272587	2.220942	-2.778543
H	1.567944	4.303542	-3.062753
H	4.054341	3.331944	0.338344
H	5.507501	0.350188	4.298157
P	-2.231057	0.310181	0.135237
C	-4.041149	0.401961	-0.367614
N	-4.963173	0.997838	0.567168
C	-5.158090	2.438029	0.561800
C	-3.911050	3.307598	0.244857
N	-2.677944	3.004901	0.942409
C	-1.741092	2.123862	0.291245
C	-5.249623	0.262873	1.782952
C	-4.407055	0.588286	3.046223
N	-2.965579	0.649189	2.872157
C	-2.275988	1.881480	3.201836
C	-2.633885	3.153431	2.386121
C	-2.324140	-0.241553	1.937198
H	-4.081904	0.923294	-1.331365
H	-4.335587	-0.634891	-0.576308
H	-5.930704	2.724088	-0.174749
H	-5.554825	2.722372	1.543230
H	-3.697184	3.246504	-0.828855
H	-4.197153	4.354350	0.434677
H	-1.556157	2.464473	-0.735037
H	-0.767287	2.164054	0.790761
H	-5.144587	-0.803056	1.548017
H	-6.305688	0.405317	2.061746
H	-4.731785	1.551234	3.456613
H	-4.676821	-0.172177	3.801677
H	-1.201355	1.686325	3.104492
H	-2.441638	2.125224	4.263042
H	-3.611627	3.526870	2.711804
H	-1.898268	3.924806	2.678059
H	-2.807898	-1.225562	1.957736
H	-1.284976	-0.409910	2.246740
F	0.093496	-4.753553	2.782291
O	3.649376	5.080230	-1.474738
C	3.358201	6.010645	-2.506005
H	2.339168	6.411895	-2.411702
H	4.078034	6.822595	-2.384814
H	3.482045	5.561853	-3.501833

Table S18. B3LYP-D3/6-31+G(d)-LANL2DZ cartesian coordinates, in Å, of the optimized structures in their triplet excited states of all Re(I) complexes investigated.

1

Re	1.905145	-0.108745	-0.465515
C	3.365436	-1.373508	-0.343252
O	4.210226	-2.166443	-0.248092
C	3.091611	1.375681	-0.150592
O	3.783265	2.286406	0.063311
N	1.421854	-0.321376	1.732012
C	1.381431	-1.533409	2.323268
C	1.014422	-1.708953	3.654102
C	0.671584	-0.589821	4.414374
C	0.714174	0.666525	3.807743
C	1.092085	0.758594	2.471810
N	0.329683	-1.684470	-0.647960
C	0.453058	-3.040020	-0.795891
C	-0.647625	-3.884827	-0.781276
C	-1.948473	-3.375110	-0.615453
C	-2.110646	-1.977653	-0.481566
C	-0.920358	-1.182799	-0.513865
C	-1.034752	0.222469	-0.405249
N	0.063322	1.027329	-0.446551
C	-0.416260	2.334527	-0.333732
C	0.309375	3.525745	-0.326858
C	-0.407086	4.724971	-0.187419
C	-1.803471	4.729084	-0.060761
C	-2.539675	3.529039	-0.069763
C	-1.843574	2.333433	-0.206505
C	-2.250890	0.934807	-0.258307
C	-3.417301	0.183339	-0.205115
C	-4.815938	0.556077	-0.051595
O	-5.337127	1.664008	0.079913
N	-5.515669	-0.669365	-0.078415
C	-4.697133	-1.795665	-0.234396
O	-5.083407	-2.962762	-0.281773
C	-3.330363	-1.261546	-0.318089
H	1.642291	-2.379141	1.698139
H	0.995854	-2.708681	4.075831
H	0.376593	-0.694487	5.454498
H	0.454027	1.568669	4.351879
H	1.124101	1.714950	1.963223
H	1.462838	-3.412555	-0.928001
H	-2.819175	-4.020615	-0.598268
H	1.389421	3.524821	-0.433314
H	0.134175	5.667069	-0.182786
H	-3.621757	3.517175	0.025803
H	-6.522679	-0.729157	0.004756
C	2.152686	0.051197	-2.377766
O	2.283194	0.142210	-3.525121
H	-0.490872	-4.952116	-0.903901
H	-2.328036	5.674720	0.043060

2

Re	-0.982657	-0.839072	-0.427650
C	-2.891549	-1.093259	-0.265659
O	-4.044046	-1.201478	-0.145599
C	-0.633093	-2.690903	-0.019601
O	-0.392124	-3.799611	0.249613
N	-0.792079	-0.225751	1.748000
C	-1.632572	0.679644	2.288164
C	-1.509484	1.127991	3.600628
C	-0.473861	0.629184	4.391923
C	0.401167	-0.304800	3.834220
C	0.208733	-0.704707	2.515196
N	-1.136279	1.346210	-0.708099
C	-2.237853	2.174592	-0.904752
C	-2.127500	3.544961	-0.964167
C	-0.865895	4.194471	-0.841278
C	0.287542	3.379852	-0.680601
C	0.079564	1.972026	-0.627675
C	1.201346	1.149049	-0.491781
N	1.095274	-0.200225	-0.450203
C	2.405670	-0.690531	-0.325356
C	2.820795	-2.014903	-0.243841
C	4.199124	-2.266890	-0.109010
C	5.123653	-1.220020	-0.060808
C	4.699634	0.121647	-0.147172
C	3.343504	0.390721	-0.278977
C	2.552262	1.618473	-0.395200
C	2.761773	2.984983	-0.434507
C	1.632495	3.843864	-0.576424
H	-2.410987	1.056596	1.635741
H	-2.214394	1.858991	3.983479
H	-0.349932	0.961942	5.418667
H	1.226432	-0.720219	4.403685
H	0.873209	-1.418253	2.041822
H	-3.191039	1.668325	-1.011794
H	-0.782114	5.275347	-0.893322
H	2.103426	-2.827937	-0.290139
H	4.546255	-3.294662	-0.046543
H	5.426371	0.929416	-0.112088
H	3.755986	3.417337	-0.365396
H	1.801582	4.917758	-0.614222
C	-1.051901	-1.230142	-2.316344
O	-1.085090	-1.448914	-3.455070
H	-3.027836	4.132979	-1.118191
H	6.182609	-1.440701	0.040786

3

Re	-0.591191	-0.961141	-0.226338
C	-2.316710	-1.773199	0.098295
O	-3.377414	-2.210487	0.300555
C	0.293801	-2.455969	0.614309
O	0.839245	-3.338228	1.146545
N	-0.667046	0.192532	1.727148
C	-1.790707	0.819870	2.127063
C	-1.855494	1.572514	3.296449
C	-0.713299	1.687537	4.090331
C	0.453365	1.041413	3.677653
C	0.436282	0.308162	2.494666
N	-1.409587	0.915739	-1.007991
C	-2.696876	1.183682	-1.314746
C	-3.152928	2.429179	-1.711645
C	-2.215737	3.500787	-1.810717
C	-0.892333	3.255348	-1.528957
C	-0.468815	1.954764	-1.135928
C	0.862519	1.582285	-0.882535
N	1.132904	0.291813	-0.534476
C	2.526069	0.164806	-0.385455
C	3.269702	-0.948424	-0.063123
C	4.687201	-0.803510	0.036819
C	5.308781	0.421045	-0.186334
C	4.547965	1.560434	-0.523502
C	3.151035	1.437926	-0.625261
C	2.113384	2.341551	-0.943143
H	-2.651147	0.716173	1.476681
H	-2.787012	2.058279	3.568929
H	-0.731624	2.267754	5.008663
H	1.370063	1.101414	4.255614
H	1.324199	-0.197744	2.133520
H	-3.382371	0.345473	-1.228617
H	-2.541980	4.491632	-2.113388
H	-0.150878	4.045596	-1.607145
H	2.804162	-1.913367	0.101390
H	5.284776	-1.675268	0.288761
H	5.032931	2.516154	-0.704543
H	2.210947	3.388358	-1.200832

C	-0.472020	-1.806567	-1.955712
O	-0.396067	-2.297916	-3.004314
H	-4.203071	2.569933	-1.943603
H	6.389207	0.501854	-0.106297

1a

Re	1.320296	-1.639330	-0.450622
C	0.729124	-3.477678	-0.268345
O	0.346036	-4.565488	-0.136051
C	3.165233	-2.167601	-0.295852
O	4.285905	-2.456840	-0.177062
N	1.128969	-1.272824	1.770420
C	0.069071	-1.737478	2.464565
C	-0.117210	-1.473546	3.818129
C	0.826568	-0.696826	4.491925
C	1.926182	-0.216437	3.778833
C	2.040805	-0.524484	2.426786
N	-0.745048	-0.779758	-0.442146
C	-1.952526	-1.401796	-0.437269
C	-3.152594	-0.708135	-0.318315
C	-3.155345	0.705536	-0.205324
C	-1.919213	1.378958	-0.219359
C	-0.738673	0.578866	-0.343633
C	0.517687	1.208644	-0.370412
N	1.673115	0.492035	-0.492799
C	2.706399	1.431329	-0.503749
C	4.078706	1.205862	-0.615811
C	4.927717	2.323663	-0.589483
C	4.418553	3.622792	-0.456841
C	3.033650	3.854890	-0.345986
C	2.181214	2.758031	-0.370140
C	0.732444	2.610501	-0.285231
C	-0.392006	3.406426	-0.151921
C	-0.556807	4.853070	-0.024682
O	0.287337	5.747269	-0.001236
N	-1.946732	5.043781	0.077255
C	-2.695650	3.856280	0.028897
O	-3.919983	3.781763	0.103551
C	-1.701328	2.785925	-0.119704
H	-0.647479	-2.329063	1.907064
H	-0.990964	-1.871335	4.324022
H	0.707555	-0.470608	5.547621
H	2.687818	0.393718	4.253473
H	2.874190	-0.160430	1.837401
H	-1.951020	-2.481847	-0.530079
H	-4.079825	1.260800	-0.113062
H	4.474547	0.201684	-0.726904
H	6.000295	2.175096	-0.678686
H	2.624952	4.856184	-0.244552
H	-2.371971	5.957179	0.174903
C	1.392014	-1.817258	-2.376810
O	1.424855	-1.907863	-3.530808
C	-4.401114	-1.517833	-0.310041
O	-4.431209	-2.737939	-0.345242
O	-5.504366	-0.747096	-0.253915
C	-6.778690	-1.437901	-0.243572
C	-7.861825	-0.377326	-0.181680
H	-6.802902	-2.109515	0.621281
H	-6.847741	-2.051798	-1.147793
H	-7.807105	0.287339	-1.050303
H	-8.847750	-0.856565	-0.172352
H	-7.762425	0.230444	0.723982
H	5.102559	4.466565	-0.442300

1b

Re	1.912106	-0.044055	-0.451847
C	3.340758	-1.342797	-0.296033
O	4.165069	-2.153465	-0.180574
C	3.139780	1.418228	-0.200987
O	3.856565	2.317349	-0.025234
N	1.446418	-0.165793	1.756084
C	1.397208	-1.353808	2.394015
C	1.042040	-1.473598	3.734163
C	0.721817	-0.321728	4.454424
C	0.774043	0.909891	3.799694
C	1.138497	0.945903	2.457334
N	0.292189	-1.583044	-0.559395
C	0.384665	-2.948056	-0.643435
C	-0.755880	-3.730937	-0.581945
C	-2.043026	-3.208296	-0.439150
C	-2.154925	-1.799158	-0.370091
C	-0.941104	-1.041333	-0.441204
C	-1.016861	0.369597	-0.390598
N	0.103415	1.141077	-0.466620
C	-0.339727	2.465148	-0.407961
C	0.418448	3.634582	-0.452628
C	-0.264348	4.858702	-0.362818
C	-1.659012	4.906541	-0.234085
C	-2.428333	3.727250	-0.190991
C	-1.765999	2.508624	-0.278471
C	-2.211465	1.120500	-0.271102
C	-3.398214	0.402216	-0.184126
C	-4.787101	0.818433	-0.043145
O	-5.276660	1.943729	0.042104
N	-5.521179	-0.387599	-0.015955
C	-4.733221	-1.538991	-0.125492
O	-5.144820	-2.696551	-0.122439
C	-3.350364	-1.043368	-0.235325
H	1.641768	-2.226515	1.800081
H	1.015800	-2.456181	4.194031
H	0.437082	-0.382689	5.500850
H	0.531988	1.835541	4.311756
H	1.177928	1.881712	1.912300
H	1.367939	-3.385533	-0.764140
H	-2.916093	-3.848156	-0.392824
H	1.497723	3.599423	-0.560736
H	0.302841	5.784643	-0.398954
H	-3.510000	3.750153	-0.093086
H	-6.529187	-0.416291	0.071966
C	2.147659	0.040779	-2.371425
O	2.270907	0.088581	-3.521823
F	-0.601635	-5.075241	-0.663346
H	-2.157408	5.869603	-0.169364

1c

Re	-1.985865	-0.567608	-0.459937
C	-3.869582	-0.142828	-0.324190
O	-4.988834	0.153765	-0.220071
C	-2.324983	-2.435721	-0.142060
O	-2.495983	-3.567188	0.073321
N	-1.642755	-0.147004	1.735454
C	-2.155911	0.953857	2.322553
C	-1.899630	1.286995	3.649292
C	-1.077968	0.453575	4.409475
C	-0.547154	-0.688249	3.807121
C	-0.851465	-0.952030	2.475328
N	-1.344568	1.564322	-0.645985
C	-2.092988	2.697928	-0.791849
C	-1.520948	3.965359	-0.776844
C	-0.136836	4.132639	-0.613405
C	0.672399	2.977772	-0.481959
C	-0.002031	1.712903	-0.514445
C	0.763137	0.534391	-0.408660
N	0.173751	-0.707529	-0.450831
C	1.203601	-1.620473	-0.346740
C	1.143167	-3.017610	-0.344985
C	2.332978	-3.741117	-0.217346
C	3.569418	-3.072391	-0.094345
C	3.643794	-1.655796	-0.095348
C	2.468859	-0.942833	-0.222419
C	2.163789	0.484384	-0.268100
C	2.839884	1.699328	-0.215146
C	4.249873	2.027280	-0.065715
O	5.230600	1.291201	0.060575
N	4.291058	3.436319	-0.089444
C	3.035022	4.042951	-0.240380

O	2.828412	5.255480	-0.284509
C	2.083270	2.927528	-0.322601
H	-2.777435	1.583458	1.697117
H	-2.335751	2.188440	4.067415
H	-0.854963	0.689247	5.446097
H	0.100492	-1.367858	4.351518
H	-0.446875	-1.820566	1.969154
H	-3.159445	2.550358	-0.922110
H	0.324384	5.113553	-0.596009
H	0.194092	-3.534116	-0.447521
H	2.289578	-4.823600	-0.220508
H	4.602454	-1.154231	-0.000611
H	5.150545	3.964260	-0.008416
C	-2.138910	-0.824503	-2.370607
O	-2.215828	-0.966336	-3.517949
H	-2.165379	4.830915	-0.897424
O	4.763862	-3.701370	0.032318
C	4.810256	-5.126220	0.024676
H	4.424310	-5.528828	-0.920585
H	5.864600	-5.385377	0.129654
H	4.242793	-5.544915	0.865911

1d

Re	-2.011819	-0.891390	-0.459706
C	-3.940106	-0.826758	-0.328819
O	-5.096290	-0.747685	-0.226992
C	-1.992780	-2.789020	-0.142700
O	-1.944271	-3.933047	0.072938
N	-1.753934	-0.410800	1.736453
C	-2.469694	0.570739	2.322725
C	-2.280492	0.950247	3.648345
C	-1.311789	0.293402	4.408341
C	-0.570948	-0.725131	3.806706
C	-0.820474	-1.045337	2.475805
N	-1.782606	1.324741	-0.641242
C	-2.729388	2.292993	-0.782096
C	-2.404515	3.649006	-0.766709
C	-1.079923	4.073813	-0.607946
C	-0.061742	3.094260	-0.479320
C	-0.488470	1.723489	-0.511219
C	0.482511	0.713051	-0.406785
N	0.141071	-0.632701	-0.445594
C	1.314290	-1.318144	-0.345681
C	1.547643	-2.707860	-0.336504
C	2.847375	-3.169850	-0.217212
C	3.962574	-2.273379	-0.105211
C	3.724095	-0.863933	-0.111542
C	2.429851	-0.407199	-0.230150
C	1.859374	0.930874	-0.273294
C	2.299957	2.255880	-0.222935
C	3.624289	2.834322	-0.080401
O	4.728505	2.286641	0.039280
N	3.408467	4.224501	-0.101087
C	2.056901	4.587428	-0.246149
O	1.635337	5.744578	-0.287192
C	1.331534	3.316561	-0.325332
H	-3.202155	1.066979	1.697371
H	-2.881843	1.751540	4.065420
H	-1.136901	0.570126	5.444100
H	0.196745	-1.265537	4.351120
H	-0.256037	-1.819427	1.969577
H	-3.750076	1.949024	-0.909215
H	-0.812899	5.124586	-0.590766
H	0.722600	-3.407578	-0.429078
H	3.011768	-4.240131	-0.218408
H	4.520977	-0.133553	-0.029211
H	4.154567	4.903093	-0.024156
C	-2.106773	-1.168308	-2.369297
O	-2.149474	-1.320144	-3.517803
H	-3.201807	4.377092	-0.883571
N	5.243254	-2.750988	0.005074
C	5.503780	-4.185820	-0.006341
C	6.370389	-1.822554	0.096625
H	4.996709	-4.688804	0.827318
H	5.169166	-4.645033	-0.945983
H	6.575704	-4.357613	0.094601
H	6.433378	-1.184189	-0.793006
H	6.281374	-1.176292	0.977859
H	7.296124	-2.392673	0.179493

1e

Re	0.384947	-2.091178	-0.439281
C	-0.788063	-3.628691	-0.294802
O	-1.511508	-4.529985	-0.184329
C	1.945922	-3.202158	-0.262328
O	2.908978	-3.843259	-0.129987
N	0.277507	-1.698038	1.784336
C	-0.894495	-1.773650	2.448775
C	-1.013383	-1.465025	3.800590
C	0.121534	-1.059411	4.504251
C	1.336931	-0.983382	3.821947
C	1.373198	-1.309260	2.469757
N	-1.274184	-0.593375	-0.449982
C	-2.616967	-0.776254	-0.477207
C	-3.520853	0.278618	-0.365105
C	-3.056842	1.609115	-0.225927
C	-1.665134	1.835630	-0.206279
C	-0.815476	0.687327	-0.325560
C	0.573959	0.864037	-0.319481
N	1.438059	-0.203112	-0.439270
C	2.708791	0.333386	-0.422529
C	3.940176	-0.323004	-0.523728
C	5.112757	0.437142	-0.474967
C	5.053932	1.838913	-0.328031
C	3.810933	2.516555	-0.224834
C	2.655085	1.765617	-0.274070
C	1.236000	2.107311	-0.209083
C	0.438903	3.234459	-0.080064
C	0.768095	4.651177	0.067086
O	1.865623	5.206463	0.116521
N	-0.476776	5.296612	0.150699
C	-1.579413	4.426226	0.071507
O	-2.758615	4.769124	0.125649
C	-0.997220	3.087763	-0.078220
H	-1.756079	-2.081821	1.868474
H	-1.983196	-1.540261	4.281628
H	0.060195	-0.807572	5.559087
H	2.248880	-0.671396	4.320684
H	2.295021	-1.251065	1.902964
H	-2.974785	-1.793310	-0.590114
H	-3.745640	2.439504	-0.139077
H	3.988286	-1.400327	-0.645101
H	6.067232	-0.068609	-0.558012
H	3.780425	3.596365	-0.113286
H	-0.574358	6.298429	0.255487
C	0.432354	-2.260877	-2.365807
O	0.457866	-2.343937	-3.520912
C	-4.966418	-0.069910	-0.392353
O	-5.400862	-1.210047	-0.449731
O	-5.753556	1.022849	-0.341038
C	-7.183836	0.793463	-0.363987
C	-7.855637	2.152679	-0.304563
H	-7.448792	0.160276	0.489642
H	-7.433415	0.245354	-1.278683
H	-8.944711	2.027734	-0.319935
H	-7.564575	2.769237	-1.161571
H	-7.579737	2.685107	0.611914
O	6.142265	2.644101	-0.273703
C	7.444135	2.073321	-0.389547
H	7.567235	1.564856	-1.354239
H	7.641243	1.369795	0.429561
H	8.138053	2.912444	-0.326363

1f

Re	-0.042913	-2.202300	-0.444894
C	-1.411397	-3.566667	-0.314556

O	-2.249097	-4.365152	-0.211725
C	1.354045	-3.512034	-0.270772
O	2.224957	-4.275027	-0.139525
N	-0.104138	-1.805398	1.780974
C	-1.278473	-1.723286	2.440066
C	-1.360723	-1.406806	3.792860
C	-0.184474	-1.163522	4.503343
C	1.033084	-1.251439	3.826369
C	1.030816	-1.572689	2.472482
N	-1.487716	-0.494104	-0.449542
C	-2.839061	-0.498203	-0.479916
C	-3.595573	0.670987	-0.366650
C	-2.961262	1.924829	-0.224073
C	-1.549574	1.969245	-0.200608
C	-0.860896	0.715738	-0.320172
C	0.536740	0.706195	-0.308501
N	1.264251	-0.473256	-0.426797
C	2.575815	-0.106051	-0.403847
C	3.734429	-0.903789	-0.495653
C	4.975994	-0.293475	-0.443178
C	5.117308	1.127047	-0.299928
C	3.941097	1.934714	-0.203789
C	2.707633	1.324941	-0.256849
C	1.349511	1.846812	-0.195631
C	0.709673	3.076610	-0.067979
C	1.229877	4.430849	0.079551
O	2.398545	4.831720	0.129770
N	0.088176	5.240958	0.162367
C	-1.124634	4.525158	0.080319
O	-2.245142	5.031421	0.133184
C	-0.727505	3.123850	-0.068861
H	-2.170905	-1.908381	1.854213
H	-2.334129	-1.350219	4.269202
H	-0.215939	-0.910459	5.559248
H	1.976731	-1.069750	4.330696
H	1.954027	-1.637398	1.908812
H	-3.329675	-1.457798	-0.596273
H	-3.534297	2.839084	-0.136410
H	3.656358	-1.980086	-0.614065
H	5.856345	-0.919017	-0.522203
H	3.977476	3.012407	-0.093272
H	0.125987	6.246402	0.266882
C	-0.009963	-2.361344	-2.370378
O	0.011279	-2.435632	-3.526896
C	-5.074119	0.517370	-0.396405
O	-5.657052	-0.554668	-0.455986
O	-5.711501	1.704582	-0.344496
C	-7.158532	1.664427	-0.368741
C	-7.646627	3.100016	-0.308164
H	-7.477849	1.154889	-1.284162
H	-7.505710	1.070653	0.483850
H	-8.742735	3.119259	-0.324319
H	-7.276171	3.673972	-1.164144
H	-7.303536	3.590673	0.608954
N	6.356031	1.712829	-0.256515
C	7.563878	0.903145	-0.369922
C	6.480655	3.165483	-0.132523
H	7.625844	0.166159	0.441240
H	7.598423	0.370022	-1.329224
H	8.438449	1.551147	-0.307966
H	6.006062	3.678985	-0.977447
H	6.017356	3.525744	0.793616
H	7.537645	3.432435	-0.115516

1g

Re	-0.848755	-2.235926	-0.454040
C	-2.482626	-3.271250	-0.310405
O	-3.475756	-3.861668	-0.200163
C	0.227714	-3.825976	-0.306228
O	0.900576	-4.767644	-0.191739
N	-0.793340	-1.867062	1.772977
C	-1.912033	-1.543328	2.454871
C	-1.904379	-1.247978	3.814736
C	-0.693755	-1.285460	4.508320
C	0.465732	-1.622512	3.807938
C	0.374923	-1.905034	2.448604
N	-1.885699	-0.253191	-0.441737
C	-3.212495	0.041009	-0.461559
C	-3.693220	1.340477	-0.344867
C	-2.793049	2.428449	-0.207474
C	-1.412969	2.154895	-0.195790
C	-1.015094	0.785887	-0.320384
C	0.354965	0.466244	-0.324052
N	0.786617	-0.821095	-0.449244
C	2.179922	-0.761533	-0.431584
C	3.093595	-1.812272	-0.529938
C	4.456096	-1.499119	-0.468619
C	4.900292	-0.173619	-0.316586
C	3.976924	0.892437	-0.221986
C	2.624605	0.593023	-0.278921
C	1.416060	1.407102	-0.211942
C	1.057888	2.735772	-0.077462
C	1.853940	3.953378	0.072689
O	3.073233	4.099776	0.120563
N	0.904967	4.987927	0.162026
C	-0.428984	4.554543	0.085384
O	-1.419283	5.279154	0.144682
C	-0.346669	3.096454	-0.069961
H	-2.831814	-1.516260	1.882520
H	-2.835128	-0.991751	4.310380
H	-0.654731	-1.057263	5.569547
H	1.432916	-1.665077	4.298235
H	1.254497	-2.161208	1.869671
H	-3.899741	-0.789832	-0.573152
H	-3.150378	3.446112	-0.116874
H	2.760470	-2.836965	-0.656503
H	5.201320	-2.284673	-0.541830
H	4.307299	1.919204	-0.106566
H	1.160017	5.961608	0.270287
C	-0.879156	-2.391803	-2.383843
O	-0.895811	-2.466045	-3.539136
C	-5.171180	1.517411	-0.366420
O	-5.973153	0.598794	-0.423385
O	-5.526336	2.815004	-0.310753
C	-6.947957	3.100270	-0.329337
C	-7.102399	4.608164	-0.266399
H	-7.374534	2.675267	-1.243955
H	-7.413997	2.597178	0.524608
H	-6.617157	5.086220	-1.123883
H	-8.166619	4.870905	-0.278441
H	-6.656063	5.008991	0.649899
C	6.373000	0.061005	-0.259939
O	7.210035	-0.824496	-0.313967
O	6.678084	1.367466	-0.140930
C	8.086385	1.704235	-0.082280
C	8.179602	3.213167	0.045799
H	8.570142	1.332140	-0.991674
H	8.532402	1.184499	0.772484
H	7.714566	3.707160	-0.813786
H	9.232343	3.515377	0.093657
H	7.676330	3.559788	0.954581

1h

Re	-1.714914	-1.111303	-0.448136
C	-3.647008	-1.141989	-0.316089
O	-4.804337	-1.118006	-0.215035
C	-1.608364	-3.012713	-0.170805
O	-1.509749	-4.156776	0.021100
N	-1.485015	-0.669677	1.757517
C	-2.260438	0.250394	2.367982
C	-2.096492	0.604955	3.703705
C	-1.090137	-0.011630	4.448568
C	-0.287270	-0.966280	3.822142
C	-0.515106	-1.265644	2.482596
N	-1.588598	1.116489	-0.580812
C	-2.587860	2.043939	-0.688625
C	-2.293346	3.399622	-0.637481
C	-1.004638	3.903853	-0.483249

C	0.047337	2.956402	-0.389939
C	-0.317368	1.569472	-0.450352
C	0.702331	0.600445	-0.374687
N	0.416706	-0.745243	-0.439730
C	1.630657	-1.394567	-0.363770
C	1.896875	-2.767908	-0.393227
C	3.223282	-3.197332	-0.292327
C	4.271885	-2.261371	-0.164557
C	4.014823	-0.865526	-0.133207
C	2.705533	-0.443229	-0.234040
C	2.075899	0.874310	-0.245143
C	2.450180	2.213771	-0.169659
C	3.747552	2.859384	-0.021998
O	4.872603	2.368984	0.079713
N	3.460358	4.239978	-0.011904
C	2.096532	4.538149	-0.139331
O	1.608715	5.666143	-0.153091
C	1.429881	3.230282	-0.242183
H	-3.022491	0.716879	1.755086
H	-2.747295	1.355368	4.140685
H	-0.934182	0.246881	5.491962
H	0.511351	-1.473081	4.354162
H	0.095812	-1.991133	1.958403
H	-3.602735	1.689156	-0.819094
H	-0.808984	4.968821	-0.445834
H	1.092387	-3.488658	-0.499564
H	3.432973	-4.259785	-0.320013
H	4.832568	-0.157593	-0.034996
H	4.174194	4.951861	0.075791
C	-1.801222	-1.357120	-2.365278
O	-1.839935	-1.490415	-3.515222
F	-3.330739	4.268089	-0.742179
O	5.580380	-2.596337	-0.063121
C	5.959373	-3.970784	-0.105078
H	5.664936	-4.431769	-1.056392
H	7.046359	-3.977173	-0.015490
H	5.517427	-4.527843	0.730983

1i

Re	1.722959	-0.119089	-0.022584
C	3.184082	-1.373903	-0.075631
O	4.036297	-2.173272	-0.127272
C	2.897359	1.349609	0.265347
O	3.600681	2.272275	0.437225
N	1.311851	-0.449952	2.141839
C	1.325056	-1.687971	2.678149
C	0.997878	-1.936395	4.007871
C	0.637234	-0.866215	4.827984
C	0.620968	0.416525	4.277535
C	0.962883	0.582538	2.939037
N	0.153627	-1.705392	-0.298455
C	0.276000	-3.053780	-0.478641
C	-0.830269	-3.884222	-0.616885
C	-2.134960	-3.363459	-0.581553
C	-2.299790	-1.968237	-0.406217
C	-1.102405	-1.193190	-0.268236
C	-1.210294	0.202463	-0.108918
N	-0.091144	0.994133	0.007655
C	-0.569311	2.299885	0.123919
C	0.162213	3.481596	0.264672
C	-0.552228	4.683488	0.365608
C	-1.955627	4.703691	0.325375
C	-2.697060	3.517508	0.182466
C	-2.002558	2.316189	0.081770
C	-2.418220	0.926673	-0.074561
C	-3.598022	0.193474	-0.196324
C	-4.998251	0.590418	-0.202411
O	-5.514684	1.704958	-0.093679
N	-5.709637	-0.616315	-0.367223
C	-4.892084	-1.751053	-0.469845
O	-5.290197	-2.906839	-0.620098
C	-3.520324	-1.240220	-0.359446
H	1.599791	-2.493893	2.008190
H	1.024323	-2.954527	4.382895
H	0.373521	-1.027916	5.869253
H	0.344491	1.283908	4.868391
H	0.953520	1.560855	2.473309
H	1.289838	-3.438734	-0.512517
H	-3.008406	-3.996391	-0.690156
H	1.246970	3.468249	0.292499
H	-0.006140	5.616649	0.475269
H	-3.782980	3.517167	0.149642
H	-6.719588	-0.659246	-0.410573
P	1.852703	0.188995	-2.395336
C	3.546353	0.342937	-3.099080
C	1.097535	-1.155597	-3.404748
C	0.997843	1.693994	-3.025999
H	4.051885	1.195215	-2.634006
H	3.515191	0.486366	-4.185092
H	4.119199	-0.560875	-2.868609
H	1.592432	-2.105328	-3.179246
H	1.189006	-0.943277	-4.475867
H	0.039831	-1.256059	-3.143693
H	1.428854	2.583588	-2.556933
H	1.093475	1.773063	-4.114764
H	-0.061291	1.649953	-2.755914
H	-0.671385	-4.949346	-0.757125
H	-2.478600	5.652739	0.405368

1j

Re	-0.467092	1.590988	-0.080542
C	-1.525375	2.637218	-1.311662
O	-2.155165	3.237286	-2.093117
C	-1.219366	2.475201	1.427146
O	-1.663094	3.009574	2.372085
N	1.303745	2.938568	-0.208913
C	1.720642	3.439772	-1.390047
C	2.867431	4.218365	-1.514751
C	3.625512	4.494532	-0.375864
C	3.199109	3.976245	0.848225
C	2.039785	3.208059	0.890504
N	0.596510	0.458754	-1.707550
C	0.468475	0.520965	-3.066754
C	1.231585	-0.271001	-3.917342
C	2.173346	-1.181665	-3.409226
C	2.331684	-1.276201	-2.004571
C	1.508062	-0.415466	-1.207305
C	1.616886	-0.476773	0.193922
N	0.825092	0.308331	1.006215
C	1.180544	-0.035472	2.311715
C	0.670977	0.468640	3.511499
C	1.190843	-0.042737	4.708079
C	2.189200	-1.031062	4.707566
C	2.704274	-1.542832	3.504987
C	2.199779	-1.044183	2.306844
C	2.478449	-1.332027	0.905323
C	3.295061	-2.176344	0.150086
C	4.285110	-3.173826	0.531966
O	4.670297	-3.533588	1.646981
N	4.757724	-3.693262	-0.690635
C	4.157642	-3.119648	-1.821448
O	4.409429	-3.417663	-2.989879
C	3.208896	-2.134947	-1.288244
H	1.113001	3.193553	-2.252627
H	3.155049	4.592536	-2.492106
H	4.527576	5.096207	-0.440944
H	3.753029	4.155653	1.764154
H	1.681689	2.780082	1.819383
H	-0.271422	1.219491	-3.443194
H	2.772321	-1.809703	-4.058911
H	-0.103376	1.228878	3.515133
H	0.810564	0.333660	5.654129
H	3.475272	-2.308032	3.487593
H	5.463918	-4.415412	-0.747377
P	-2.165667	-0.102640	-0.065401
C	-3.813393	0.339806	0.726239
N	-4.893857	-0.598814	0.560462
C	-5.035003	-1.702216	1.496030

C	-3.720629	-2.332238	2.033200
N	-2.684719	-2.651403	1.071164
C	-1.655186	-1.673941	0.852202
C	-5.468064	-0.732856	-0.764341
C	-4.913258	-1.851818	-1.687140
N	-3.467485	-1.950836	-1.794990
C	-2.823647	-3.194876	-1.420580
C	-2.951517	-3.656986	0.056671
C	-2.667289	-0.752488	-1.760803
H	-4.089818	1.312691	0.298262
H	-3.625128	0.524689	1.790225
H	-5.612815	-1.389072	2.384027
H	-3.276218	-1.657179	2.773884
H	-4.004311	-3.241004	2.586354
H	-1.247223	-1.329938	1.809651
H	-1.761705	-3.099818	-1.675475
H	-0.812451	-2.112603	0.307017
H	-5.351843	0.235149	-1.265987
H	-6.553504	-0.898728	-0.681209
H	-5.631254	-2.478698	1.003676
H	-5.368134	-1.686541	-2.680565
H	-5.287170	-2.821985	-1.340680
H	-3.209721	-4.015111	-2.045351
H	-3.963319	-4.040591	0.229583
H	-2.270735	-4.518425	0.174841
H	-3.177274	0.062568	-2.288621
H	-1.726599	-0.918757	-2.298894
H	1.084905	-0.178660	-4.989543
H	2.569308	-1.406790	5.653646

1k

Re	-0.894707	-1.756947	-0.045677
C	-0.832502	-3.257422	-1.247633
O	-0.788966	-4.134003	-2.021014
C	-0.812437	-2.886469	1.486543
O	-0.764486	-3.555168	2.447685
N	-3.112143	-1.631162	-0.044400
C	-3.824116	-1.790165	-1.179668
C	-5.207211	-1.644312	-1.227679
C	-5.895045	-1.318364	-0.057758
C	-5.162807	-1.151255	1.118920
C	-3.781563	-1.314800	1.084967
N	-1.051193	-0.236231	-1.696335
C	-1.066196	-0.404243	-3.054201
C	-1.122637	0.671451	-3.930294
C	-1.157826	1.992991	-3.451834
C	-1.137605	2.203271	-2.053240
C	-1.093393	1.034281	-1.226718
C	-1.056765	1.192669	0.175598
N	-0.983669	0.109383	1.011893
C	-0.908986	0.636178	2.304013
C	-0.806832	-0.051087	3.514960
C	-0.721409	0.708269	4.691139
C	-0.736415	2.111461	4.656345
C	-0.840551	2.808172	3.439217
C	-0.928001	2.068499	2.263808
C	-1.026316	2.431203	0.854551
C	-1.070287	3.580760	0.072718
C	-1.052528	4.995893	0.413542
O	-0.997261	5.551424	1.512248
N	-1.114887	5.661529	-0.827859
C	-1.166128	4.803949	-1.936354
O	-1.227340	5.158712	-3.112762
C	-1.135031	3.449506	-1.368277
H	-3.254615	-2.034153	-2.068442
H	-5.726052	-1.782988	-2.170781
H	-6.974318	-1.195903	-0.063176
H	-5.646071	-0.894163	2.056067
H	-3.177714	-1.184317	1.975090
H	-1.019681	-1.429376	-3.405992
H	-1.186824	2.844830	-4.121572
H	-0.785712	-1.135956	3.543659
H	-0.638320	0.197102	5.646493
H	-0.851422	3.893687	3.395602
H	-1.115745	6.670006	-0.912573
P	1.460809	-1.516974	-0.113390
C	2.182897	-0.953513	-1.738197
N	3.629555	-0.768183	-1.710400
C	4.114634	0.529616	-1.742013
O	3.347716	1.490963	-1.780941
C	5.620229	0.724928	-1.760482
C	4.396727	-1.925052	-1.218379
N	4.022141	-2.392319	0.113848
C	4.338724	-1.490930	1.213022
N	3.578032	-0.226234	1.218787
C	4.246154	0.962039	1.454770
O	5.473380	0.985221	1.564770
C	3.421782	2.234195	1.539749
C	2.126230	-0.324447	1.161089
C	2.652025	-2.911661	0.181216
H	1.922926	-1.732630	-2.468682
H	1.709625	-0.022518	-2.049201
H	6.120649	0.049816	-2.462740
H	6.043277	0.580447	-0.760301
H	5.808201	1.758281	-2.056557
H	5.456783	-1.677443	-1.213388
H	4.239640	-2.760813	-1.911574
H	5.394747	-1.225340	1.183724
H	4.141223	-2.033595	2.147765
H	2.958624	2.455429	0.571303
H	4.102031	3.046993	1.797540
H	2.633053	2.172169	2.298262
H	1.710461	-0.659035	2.123222
H	1.684187	0.645384	0.940153
H	2.471144	-3.365144	1.162136
H	2.504461	-3.690715	-0.575596
H	-1.128502	0.477517	-4.998681
H	-0.665250	2.669718	5.585663

1l

Re	1.348730	-1.391193	-0.017786
C	0.909713	-3.274302	0.044734
O	0.611775	-4.402380	0.065794
C	3.212821	-1.762103	0.058626
O	4.366791	-1.965724	0.099752
N	1.117896	-1.119710	2.180332
C	0.099697	-1.693967	2.854651
C	-0.115716	-1.484775	4.213591
C	0.750765	-0.645336	4.915306
C	1.804913	-0.048120	4.221650
C	1.954499	-0.309168	2.863247
N	-0.793747	-0.717178	-0.059772
C	-1.945740	-1.428334	-0.071171
C	-3.202801	-0.826753	-0.092462
C	-3.321512	0.586633	-0.108240
C	-2.141293	1.358181	-0.103655
C	-0.898485	0.646130	-0.076869
C	0.302780	1.362302	-0.089402
N	1.524468	0.718246	-0.086526
C	2.479236	1.734892	-0.130218
C	3.871447	1.615315	-0.149946
C	4.628089	2.794398	-0.193136
C	4.013554	4.056311	-0.217922
C	2.613323	4.183245	-0.199887
C	1.848138	3.022463	-0.156123
C	0.411024	2.769882	-0.131481
C	-0.779496	3.487220	-0.147990
C	-1.050950	4.924298	-0.188751
O	-0.272088	5.877531	-0.213815
N	-2.452282	5.020639	-0.194786
C	-3.109124	3.776877	-0.164732
O	-4.328993	3.620199	-0.166979
C	-2.034315	2.779813	-0.132871
H	-0.557652	-2.331504	2.275419
H	-0.952839	-1.973121	4.702037
H	0.607412	-0.459882	5.975958
H	2.506039	0.615825	4.717146
H	2.754842	0.141227	2.288049

H	-1.858458	-2.509233	-0.068440
H	-4.289667	1.070051	-0.128521
H	4.350057	0.641775	-0.134313
H	5.712609	2.726704	-0.209538
H	2.122849	5.152275	-0.219187
H	-2.946122	5.903323	-0.222045
P	1.370840	-1.466888	-2.417126
C	2.265214	-2.887038	-3.171221
C	-0.291556	-1.546729	-3.207607
C	2.138176	-0.001244	-3.226623
H	3.307628	-2.874263	-2.837556
H	2.232165	-2.841848	-4.265616
H	1.809593	-3.823365	-2.833734
H	-0.822650	-2.437155	-2.856739
H	-0.875519	-0.668394	-2.917298
H	-0.206602	-1.581719	-4.299479
H	3.176564	0.098355	-2.896659
H	1.599964	0.902132	-2.924634
H	2.108987	-0.095294	-4.317940
C	-4.379258	-1.733133	-0.101143
O	-4.314599	-2.953718	-0.075730
O	-5.544768	-1.055289	-0.140515
C	-6.755813	-1.848517	-0.154069
C	-7.923824	-0.880979	-0.202871
H	-6.772417	-2.475556	0.743954
H	-6.728669	-2.511906	-1.025258
H	-7.921182	-0.221603	0.671586
H	-7.877325	-0.258489	-1.102747
H	-8.867055	-1.439750	-0.214623
H	4.629762	4.950486	-0.252379

H	3.652655	3.839465	-0.140091
H	-0.751608	6.471730	-0.179369
P	0.438137	-1.981790	-2.409167
C	0.710932	-3.633747	-3.173171
C	-1.109211	-1.395397	-3.220911
C	1.730558	-0.933540	-3.200753
H	1.669855	-4.034834	-2.829909
H	0.711826	-3.572050	-4.267354
H	-0.080469	-4.317135	-2.849388
H	-1.953254	-2.006997	-2.886940
H	-1.027916	-1.454249	-4.312087
H	-1.305195	-0.359928	-2.927258
H	2.720121	-1.251697	-2.858887
H	1.681256	-1.005510	-4.293083
H	1.587210	0.108056	-2.898205
C	-4.999756	-0.032726	-0.150122
O	-5.409224	-1.184694	-0.138582
O	-5.816035	1.040896	-0.188695
C	-7.238114	0.773222	-0.218571
C	-7.944960	2.115446	-0.261473
H	-7.503090	0.192125	0.671418
H	-7.459505	0.158561	-1.097802
H	-9.030299	1.961898	-0.285202
H	-7.654181	2.680960	-1.153154
H	-7.697704	2.714924	0.621125
O	6.042014	2.935780	-0.159359
C	7.353762	2.381655	-0.174200
H	7.513305	1.763064	-1.067200
H	7.542836	1.784469	0.727605
H	8.032975	3.235180	-0.195530

1m

Re	0.416074	-1.921186	-0.014792
C	-0.707042	-3.488887	0.030460
O	-1.412999	-4.419529	0.043963
C	1.994228	-2.976051	0.075708
O	2.987870	-3.598956	0.125750
N	0.280628	-1.584452	2.183141
C	-0.888106	-1.728735	2.842297
C	-1.026086	-1.453413	4.199607
C	0.084942	-1.007223	4.916737
C	1.296234	-0.856461	4.239183
C	1.353111	-1.154065	2.881177
N	-1.300493	-0.471726	-0.068961
C	-2.635278	-0.686244	-0.097465
C	-3.566026	0.352741	-0.123169
C	-3.134047	1.702202	-0.125408
C	-1.747674	1.963511	-0.102973
C	-0.872883	0.828436	-0.072890
C	0.510126	1.031700	-0.066314
N	1.398680	-0.033067	-0.057910
C	2.658868	0.537429	-0.080728
C	3.906038	-0.094487	-0.090651
C	5.060092	0.695892	-0.116112
C	4.968045	2.103407	-0.133642
C	3.711630	2.755249	-0.125491
C	2.571623	1.974529	-0.099678
C	1.145428	2.289787	-0.091722
C	0.321133	3.409736	-0.111951
C	0.622799	4.840766	-0.139487
O	1.709361	5.420870	-0.146934
N	-0.633648	5.467166	-0.157364
C	-1.717505	4.568830	-0.146879
O	-2.904104	4.893070	-0.161752
C	-1.106857	3.236603	-0.116198
H	-1.730253	-2.069372	2.251774
H	-1.992602	-1.586787	4.674944
H	0.008730	-0.781621	5.976577
H	2.190688	-0.510040	4.747093
H	2.272161	-1.042442	2.318107
H	-2.969306	-1.717929	-0.105238
H	-3.842300	2.520319	-0.148451
H	3.982367	-1.176874	-0.081094
H	6.026859	0.206572	-0.124379

1n

Re	0.945731	0.144313	1.551985
C	0.977854	-1.330736	2.808877
O	0.969562	-2.234723	3.546486
C	2.646996	0.762680	2.136149
O	3.698000	1.159834	2.472997
N	-0.226719	1.458350	2.916990
C	-1.265513	0.979968	3.633083
C	-2.062595	1.790919	4.435547
C	-1.785418	3.157050	4.504503
C	-0.713203	3.656684	3.762871
C	0.038493	2.780409	2.986477
N	-1.078278	-0.380764	0.732322
C	-1.914645	-1.403176	1.026843
C	-3.134753	-1.589029	0.379055
C	-3.550125	-0.693433	-0.639894
C	-2.699287	0.379846	-0.977281
C	-1.469397	0.488002	-0.250628
C	-0.576995	1.517591	-0.560112
N	0.636698	1.642459	0.093835
C	1.250183	2.757891	-0.479521
C	2.494104	3.317054	-0.172850
C	2.899475	4.446961	-0.894702
C	2.088545	5.004490	-1.897024
C	0.837572	4.448059	-2.210242
C	0.418627	3.325760	-1.500510
C	-0.783156	2.501328	-1.550922
C	-1.974731	2.425804	-2.266414
C	-2.514370	3.250989	-3.348426
O	-2.038834	4.243151	-3.901602
N	-3.750942	2.663457	-3.660272
C	-4.055772	1.533010	-2.879154
O	-5.080459	0.859981	-2.980426
C	-2.912111	1.375096	-1.975824
H	-1.456969	-0.082992	3.545776
H	-2.885759	1.349832	4.988463
H	-2.391200	3.816627	5.119279
H	-0.456564	4.711152	3.776322
H	0.872786	3.129856	2.389629
H	-1.594714	-2.096979	1.796450
H	-4.491807	-0.826181	-1.156584
H	3.127321	2.886300	0.595732
H	3.863585	4.897628	-0.674098

H	0.197862	4.866683	-2.981911
H	-4.366663	3.017670	-4.380639
P	1.960162	-1.240694	-0.125886
C	3.349497	-2.377043	0.436523
N	3.835211	-3.336837	-0.521443
C	4.877840	-2.951919	-1.458799
C	4.838536	-1.490945	-1.983833
N	3.567074	-0.977578	-2.454950
C	2.756883	-0.246933	-1.519442
C	2.956854	-4.436983	-0.871013
C	2.056594	-4.273854	-2.125346
N	1.317226	-3.027995	-2.236059
C	1.526337	-2.191514	-3.400772
C	2.945594	-1.596456	-3.613717
C	0.793872	-2.400598	-1.050399
H	4.167264	-1.738277	0.790651
H	2.965990	-2.894929	1.325334
H	5.877285	-3.092347	-1.009507
H	4.827729	-3.640895	-2.309475
H	5.185869	-0.816124	-1.192596
H	5.586243	-1.420720	-2.788986
H	3.355671	0.523403	-1.018913
H	1.953202	0.288290	-2.036486
H	2.322841	-4.634255	0.001421
H	3.551500	-5.351267	-1.022065
H	2.672508	-4.374225	-3.026247
H	1.367769	-5.137640	-2.127309
H	1.277327	-2.755689	-4.312778
H	0.798693	-1.373369	-3.347806
H	3.618713	-2.385240	-3.967927
H	2.858252	-0.870706	-4.441341
H	0.438079	-3.156095	-0.340062
H	-0.082546	-1.794692	-1.306412
C	-3.947217	-2.755713	0.805681
O	-3.624774	-3.543119	1.684131
O	-5.098947	-2.857615	0.109965
C	-5.965146	-3.966031	0.449766
C	-7.181817	-3.875966	-0.452813
H	-6.228185	-3.891711	1.510556
H	-5.410186	-4.899654	0.307261
H	-7.869932	-4.700051	-0.230314
H	-6.891096	-3.940498	-1.506747
H	-7.711542	-2.929639	-0.300084
H	2.434493	5.880334	-2.439023

1o

Re	-0.412900	0.774480	1.626584
C	0.530472	2.167612	2.576006
O	1.129920	3.001985	3.131784
C	-2.075198	1.293233	2.386987
O	-3.119613	1.591675	2.833026
N	0.045800	-0.772960	3.164661
C	1.268378	-0.859593	3.729138
C	1.610155	-1.859747	4.634702
C	0.655711	-2.818928	4.976531
C	-0.610210	-2.734045	4.393848
C	-0.874743	-1.702214	3.498697
N	1.433039	-0.043819	0.638111
C	2.719051	0.369831	0.690951
C	3.741441	-0.252332	-0.025843
C	3.459443	-1.364099	-0.858450
C	2.126304	-1.818787	-0.943385
C	1.149358	-1.116240	-0.164852
C	-0.190043	-1.508258	-0.232101
N	-1.178455	-0.839810	0.477702
C	-2.361295	-1.488132	0.159303
C	-3.657623	-1.212533	0.602774
C	-4.711474	-2.004917	0.134817
C	-4.472203	-3.060734	-0.769391
C	-3.165698	-3.349793	-1.225721
C	-2.123229	-2.567638	-0.761879
C	-0.685403	-2.570189	-1.015230
C	0.243025	-3.276945	-1.774126
C	0.092520	-4.408646	-2.689574
O	-0.916983	-5.034296	-3.016364
N	1.391985	-4.662072	-3.156293
C	2.365275	-3.793587	-2.626162
O	3.565008	-3.829119	-2.896483
C	1.628051	-2.897001	-1.731455
H	1.985640	-0.103446	3.432776
H	2.610538	-1.879102	5.055162
H	0.892723	-3.613648	5.678135
H	-1.388543	-3.455597	4.621070
H	-1.841576	-1.606653	3.018630
H	2.936096	1.227829	1.317680
H	4.239502	-1.855143	-1.425731
H	-3.849686	-0.396854	1.292214
H	-5.716692	-1.789602	0.477496
H	-2.991925	-4.164436	-1.922344
H	1.610866	-5.398612	-3.814332
P	-0.751574	2.224878	-0.252035
C	-1.135009	4.026257	0.127943
N	-1.150222	4.942079	-0.984294
C	-2.369942	5.113138	-1.756163
C	-3.253147	3.851182	-1.955188
N	-2.602053	2.626816	-2.377880
C	-2.192203	1.704007	-1.352052
C	0.119848	5.252538	-1.612141
C	0.540327	4.415344	-2.850410
N	0.414020	2.972921	-2.732590
C	-0.428226	2.264939	-3.675682
C	-1.946313	2.593745	-3.674850
C	0.684501	2.333513	-1.470691
H	-2.094675	4.048575	0.657907
H	-0.380172	4.339141	0.861033
H	-3.021277	5.875467	-1.291859
H	-2.087398	5.512442	-2.736845
H	-3.777557	3.630343	-1.017858
H	-4.035887	4.122380	-2.680823
H	-3.025328	1.510031	-0.665203
H	-1.933969	0.733007	-1.788330
H	0.892665	5.161550	-0.839802
H	0.130143	6.308790	-1.923269
H	-0.053228	4.729780	-3.716403
H	1.584363	4.698509	-3.075644
H	-0.065308	2.438667	-4.700632
H	-0.293629	1.193023	-3.489414
H	-2.104486	3.568302	-4.150498
H	-2.425962	1.848303	-4.333737
H	1.520967	2.825935	-0.961083
H	1.005729	1.299533	-1.640391
C	5.105617	0.312156	0.127561
O	5.387790	1.263956	0.841657
O	6.017741	-0.347175	-0.617276
C	7.382517	0.127248	-0.539829
C	8.214745	-0.753909	-1.453156
H	7.712787	0.073658	0.503292
H	7.404815	1.179322	-0.844138
H	9.262026	-0.430396	-1.426767
H	7.857369	-0.691718	-2.486547
H	8.165962	-1.800914	-1.135519
O	-5.445070	-3.870436	-1.268582
C	-6.796411	-3.658121	-0.875337
H	-7.144735	-2.659736	-1.171731
H	-7.378242	-4.417665	-1.399850
H	-6.920234	-3.785144	0.208431

1p

Re	1.740478	-0.068018	-0.004699
C	3.153059	-1.385026	0.013457
O	3.971461	-2.218118	0.009985
C	2.973207	1.362877	0.223235
O	3.712918	2.262421	0.357727
N	1.312454	-0.292583	2.169581
C	1.275814	-1.506327	2.758097
C	0.932304	-1.684871	4.094848
C	0.607531	-0.567574	4.865622
C	0.642783	0.690183	4.260948

C	0.998827	0.785807	2.919348
N	0.111517	-1.603417	-0.211059
C	0.187441	-2.963669	-0.333012
C	-0.969236	-3.724624	-0.424502
C	-2.253447	-3.183830	-0.407800
C	-2.351909	-1.772877	-0.296133
C	-1.121875	-1.041577	-0.199041
C	-1.174325	0.362561	-0.110055
N	-0.020717	1.114907	-0.039953
C	-0.447764	2.443009	0.014857
C	0.329516	3.601146	0.095234
C	-0.337206	4.833310	0.142210
C	-1.738900	4.905522	0.108747
C	-2.526393	3.743294	0.027368
C	-1.879659	2.512539	-0.019628
C	-2.349915	1.134107	-0.104837
C	-3.559054	0.440077	-0.184566
C	-4.944647	0.890823	-0.202899
O	-5.414657	2.027938	-0.145424
N	-5.705313	-0.292751	-0.306192
C	-4.932126	-1.460141	-0.358262
O	-5.370586	-2.605355	-0.450638
C	-3.538363	-0.996273	-0.278852
H	1.522655	-2.350512	2.125421
H	0.918166	-2.686256	4.513054
H	0.331707	-0.674461	5.910793
H	0.395703	1.591783	4.812251
H	1.029172	1.742916	2.412134
H	1.169522	-3.420617	-0.357413
H	-3.136487	-3.806872	-0.484399
H	1.412987	3.547371	0.117026
H	0.245223	5.748662	0.203824
H	-3.611665	3.784199	0.001292
H	-6.716413	-0.297839	-0.342806
P	1.862140	0.154080	-2.393879
C	3.426590	-0.376784	-3.204742
C	0.565628	-0.778204	-3.312905
C	1.637443	1.875917	-3.005807
H	4.259700	0.201600	-2.792879
H	3.380777	-0.226639	-4.289218
H	3.608799	-1.435007	-2.993125
H	0.657376	-1.847231	-3.098686
H	0.653032	-0.612288	-4.392369
H	-0.422359	-0.449328	-2.975774
H	2.435778	2.509162	-2.606063
H	1.658040	1.912875	-4.100854
H	0.681103	2.266154	-2.645720
F	-0.825190	-5.070977	-0.538583
H	-2.224681	5.876697	0.145933

1q

Re	-1.694159	-0.787103	-0.008634
C	-3.607190	-0.572427	0.024710
O	-4.763116	-0.394448	0.029797
C	-1.831892	-2.673779	0.173543
O	-1.883177	-3.841599	0.279116
N	-1.459189	-0.403221	2.174329
C	-2.146771	0.580169	2.791592
C	-1.965986	0.897560	4.134607
C	-1.035210	0.174649	4.882182
C	-0.321384	-0.843860	4.248241
C	-0.560874	-1.100722	2.901873
N	-1.288948	1.419285	-0.168924
C	-2.156235	2.470557	-0.251715
C	-1.676185	3.771989	-0.328749
C	-0.323205	4.096922	-0.333035
C	0.596078	3.016967	-0.256603
C	0.039360	1.696475	-0.173943
C	0.916279	0.597486	-0.111770
N	0.435930	-0.699925	-0.049368
C	1.557133	-1.508151	-0.024657
C	1.631333	-2.903384	0.030423
C	2.891640	-3.509140	0.044334
C	4.065291	-2.726126	0.001658
C	4.003671	-1.312891	-0.055504
C	2.758069	-0.714250	-0.068879
C	2.317003	0.676273	-0.127702
C	2.879710	1.953537	-0.198474
C	4.261965	2.410660	-0.231455
O	5.314753	1.770412	-0.200380
N	4.172326	3.815878	-0.311313
C	2.856146	4.298261	-0.334883
O	2.530648	5.482668	-0.403684
C	2.010699	3.097708	-0.260915
H	-2.853306	1.125391	2.177338
H	-2.546717	1.701366	4.575702
H	-0.869228	0.400168	5.931712
H	0.416655	-1.435195	4.780794
H	-0.021069	-1.876872	2.372489
H	-3.218432	2.257549	-0.262008
H	0.018077	5.123224	-0.397181
H	0.730707	-3.508332	0.059473
H	2.951127	-4.590196	0.086143
H	4.914518	-0.722364	-0.088396
H	4.982767	4.420082	-0.351048
P	-1.717068	-0.968544	-2.396375
C	-3.052737	-2.010938	-3.115374
C	-1.892674	0.625323	-3.305056
C	-0.187033	-1.682883	-3.134079
H	-2.970908	-3.026078	-2.714038
H	-2.987549	-2.044846	-4.208826
H	-4.026498	-1.605879	-2.822369
H	-2.828783	1.112100	-3.014438
H	-1.888522	0.463834	-4.388851
H	-1.067603	1.290906	-3.034405
H	-0.019411	-2.685758	-2.729802
H	-0.265850	-1.738324	-4.225641
H	0.671179	-1.061092	-2.862786
F	-2.593818	4.771699	-0.406218
O	5.322322	-3.241095	0.011198
C	5.498502	-4.653512	0.062598
H	5.050955	-5.141312	-0.813278
H	6.577445	-4.815078	0.058747
H	5.067466	-5.074169	0.980604

1r

Re	-0.486989	1.593943	-0.018754
C	-1.513802	2.618997	-1.299855
O	-2.122878	3.204923	-2.106232
C	-1.279991	2.509371	1.448714
O	-1.750343	3.063771	2.368373
N	1.285120	2.941677	-0.134997
C	1.731060	3.415388	-1.316973
C	2.880884	4.191050	-1.431256
C	3.610506	4.493519	-0.280536
C	3.153742	4.003904	0.944455
C	1.993411	3.236806	0.975990
N	0.618234	0.427779	-1.592999
C	0.523067	0.465217	-2.958027
C	1.323253	-0.356728	-3.738516
C	2.249707	-1.257168	-3.216670
C	2.361242	-1.313610	-1.802349
C	1.514389	-0.434388	-1.048672
C	1.584553	-0.465156	0.355527
N	0.768773	0.338656	1.128463
C	1.088296	0.023672	2.450655
C	0.546146	0.553332	3.625020
C	1.033154	0.068844	4.846219
C	2.031298	-0.918465	4.894414
C	2.579013	-1.455873	3.717830
C	2.107536	-0.984109	2.495508
C	2.424757	-1.302151	1.108843
C	3.261885	-2.162872	0.392044
C	4.242359	-3.152787	0.822265
O	4.595747	-3.486700	1.954107
N	4.749116	-3.699920	-0.374648
C	4.179352	-3.150740	-1.531528
O	4.458912	-3.470635	-2.685825

C	3.215123	-2.153192	-1.043621
H	1.145450	3.149153	-2.188828
H	3.192719	4.542318	-2.409656
H	4.514199	5.093567	-0.337234
H	3.684970	4.204716	1.869326
H	1.612207	2.830405	1.905401
H	-0.195865	1.143149	-3.401985
H	2.855263	-1.888402	-3.855887
H	-0.228058	1.312934	3.591400
H	0.627198	0.465652	5.773027
H	3.350353	-2.220545	3.738973
H	5.456380	-4.422914	-0.396103
P	-2.189330	-0.098548	-0.023683
C	-3.846574	0.338097	0.751769
N	-4.920567	-0.605221	0.573740
C	-5.067626	-1.709109	1.507772
C	-3.756598	-2.334811	2.058266
N	-2.710003	-2.648403	1.105887
C	-1.681446	-1.668679	0.899810
C	-5.480817	-0.740839	-0.757186
C	-4.911897	-1.857263	-1.674384
N	-3.464556	-1.950694	-1.764898
C	-2.819701	-3.192767	-1.386362
C	-2.961137	-3.656142	0.089150
C	-2.669786	-0.749688	-1.724723
H	-4.122544	1.310246	0.321849
H	-3.669848	0.522509	1.817811
H	-5.655214	-1.397908	2.389896
H	-5.656308	-2.487377	1.009342
H	-3.322311	-1.659071	2.804256
H	-4.042075	-3.245608	2.606882
H	-1.286168	-1.322086	1.861563
H	-0.831187	-2.103205	0.363302
H	-5.363218	0.227857	-1.257132
H	-6.566301	-0.910730	-0.685038
H	-5.355439	-1.693068	-2.672911
H	-5.286051	-2.829047	-1.332829
H	-3.196353	-4.013571	-2.015909
H	-1.755632	-3.093552	-1.630552
H	-3.972928	-4.044124	0.251522
H	-2.277574	-4.514004	0.215686
H	-3.177566	0.062801	-2.258445
H	-1.722692	-0.912184	-2.252685
F	1.179624	-0.270035	-5.087846
H	2.385656	-1.273157	5.858421

1s

Re	-0.743140	-0.835971	-1.377064
C	-2.078451	-2.163130	-1.793156
O	-2.873047	-2.995062	-2.003474
C	-1.275956	0.266307	-2.830126
O	-1.584767	0.974461	-3.714317
N	0.866944	-1.887546	-2.506664
C	1.019455	-3.225987	-2.431156
C	2.059931	-3.902194	-3.061535
C	2.990704	-3.172846	-3.802875
C	2.837821	-1.787448	-3.882751
C	1.769338	-1.186013	-3.225070
N	0.082576	-1.992703	0.366560
C	-0.317788	-3.182558	0.903794
C	0.338048	-3.708148	2.009863
C	1.410952	-3.085417	2.639973
C	1.836840	-1.841353	2.102410
C	1.124856	-1.353371	0.955626
C	1.498066	-0.113052	0.406377
N	0.826684	0.421335	-0.682666
C	1.445754	1.633564	-0.937051
C	1.156635	2.575069	-1.928762
C	1.920239	3.745232	-1.988009
C	2.961449	3.971640	-1.062411
C	3.264248	3.026925	-0.054720
C	2.509918	1.868843	0.003184
C	2.530803	0.706420	0.885226
C	3.244250	0.244031	1.995875
C	4.350775	0.815480	2.751497
O	4.959975	1.875818	2.596129
N	4.616853	-0.127799	3.765440
C	3.776894	-1.249893	3.727294
O	3.818356	-2.199002	4.509180
C	2.884582	-1.017117	2.582548
H	0.283803	-3.756444	-1.838409
H	2.132547	-4.980623	-2.963130
H	3.815595	-3.671141	-4.304091
H	3.534768	-1.171370	-4.441988
H	1.621622	-0.112926	-3.255064
H	-1.159189	-3.691716	0.449358
H	1.896919	-3.520773	3.504945
H	0.351815	2.408757	-2.637687
H	1.695355	4.476712	-2.755105
H	4.067830	3.212118	0.651904
H	5.344391	-0.005383	4.457557
P	-2.246335	0.306047	0.100883
C	-4.055228	0.369740	-0.410099
N	-4.991970	0.883186	0.556722
C	-5.231659	2.314871	0.634032
C	-4.013510	3.241219	0.370199
N	-2.770662	2.938025	1.051730
C	-1.810880	2.123730	0.353370
C	-5.258416	0.069771	1.727495
C	-4.428168	0.350108	3.009176
N	-2.990651	0.470221	2.838525
C	-2.338101	1.698502	3.246062
C	-2.732543	3.004433	2.503286
C	-2.316191	-0.352194	1.867657
H	-4.108688	0.946367	-1.341215
H	-4.321200	-0.660656	-0.679812
H	-6.012727	2.618640	-0.086044
H	-5.637128	2.528752	1.629430
H	-3.797863	3.250421	-0.704881
H	-4.332298	4.264928	0.621525
H	-1.640655	2.523344	-0.654122
H	-0.838252	2.158812	0.855786
H	-5.118464	-0.976887	1.432261
H	-6.319102	0.162393	2.008552
H	-4.670619	-0.461231	3.719119
H	-4.784962	1.276399	3.473592
H	-2.515773	1.875100	4.318224
H	-1.257989	1.540895	3.144796
H	-3.721588	3.329508	2.845588
H	-2.020801	3.779607	2.838566
H	-2.761693	-1.353335	1.835180
H	-1.272277	-0.499982	2.167485
F	-0.110378	-4.896995	2.493859
O	3.743539	5.084277	-1.055960
C	3.517245	6.100926	-2.026370
H	2.507342	6.521823	-1.932955
H	4.256096	6.876202	-1.817697
H	3.664845	5.718397	-3.045062

References related to Discussion 1:

1) Gabrielsson, A.; Busby, M.; Matousek, P.; Towrie, M.; Hevia, E.; Cuesta, L.; Pérez, J.; Záliš, S.; Vlček Jr., A. Electronic structure and excited states of rhenium(I) amido and phosphido carbonyl-bipyridine complexes studied by picosecond time-resolved IR spectroscopy and DFT calculations. *Inorg. Chem.* **2006**, *45*, 9789-9797.

2) Cannizzo, A.; Blanco-Rodríguez, A.M.; El Nahhas, A.; Šebera, J.; Záliš, S.; Vlček Jr., A.; Chergui, M. *J. Am. Chem. Soc.* **2008**, *130*, 8967-8974.

3) Li, G.; Parimal, K.; Vyas, S.; Hadad, C.M.; Flood, A.H.; Glusac, K.D. Pinpointing the extent of electronic delocalization in the Re(I)-to-tetrazine charge-separated excited state using time-resolved infrared spectroscopy. *J. Am. Chem. Soc.* **2009**, *131*, 11656-11657.

4) Zhang, T.T.; Jia, J.F.; Wu, H.S. Substituent and solvent effects on electronic structure and spectral property of $ReCl(CO)_3(N\wedge N)(N\wedge N$ = Glyoxime): DFT and TDDFT theoretical studies. *J. Phys. Chem. A* **2010**, *114*, 12251-12257.

5) Záliš, S.; Consani, C.; El Nahhas, A.; Cannizzo, A.; Chergui, M.; Hartl, F.; Vlček Jr., A. Origin of electronic absorption spectra of MLCT-excited and one-electron reduced 2,2'-bipyridine and 1,10-phenanthroline complexes. *Inorg. Chim. Acta.* **2011**, *374*, 578-585.

6) Zhao, F.; Wang, J.X.; Liu, W.Q.; Wang, Y.B. Electronic structures and spectral properties of rhenium(I) tricarbonyl diimine complexes with phosphine ligands: DFT/TDDFT theoretical investigations, *Comput. Theor. Chem.* **2012**, *985*, 90-96.

7) Machura, B.; Wolff, M.; Benoist, E.; Coulais, Y. Tricarbonyl rhenium(I) complex of benzothiazole: Synthesis, spectroscopic characterization, X-ray crystal structure and DFT calculations. *J. Organomet. Chem.* **2012**, *724*, 82-87.

8) Chartrand, D.; Castro-Ruiz, C.A.; Hanan, G.S. Diimine triscarbonyl Re(I) of isomeric pyridyl-fulvene ligands: An electrochemical, spectroscopic, and computational investigation. *Inorg. Chem.* **2012**, *51*, 12738-12747.

9) Xia, H.; Zhao, F.; Liu, W.; Wang, Y. Electronic structures and spectral properties of rhenium (I) tricarbonyl cyclopenta[b]dipyridine complexes containing different aromatic ring groups. *J. Organomet. Chem.* **2013**, *727*, 10-18.

10) Baranovskii, V.I.; D.A. Maltsev, DFT study of potential energy surfaces and conical intersection structures of rhenium(I) tricarbonyl diimine complexes. *Comput. Theor. Chem.* **2014**, *1043*, 71-78.

11) Blanco-Rodríguez, A.M.; Kvapilová, Sýkora, J; Towrie, M.; Nervi, C.; Volpi, G.; Záliš, S.; Vlček Jr., A. Photophysics of singlet and triplet intraligand excited states in $[ReCl(CO)_3$(1-(2-pyridyl)-imidazo[1,5-α]pyridine)] complexes. *J. Am. Chem. Soc.* **2014**, *136*, 5963-5973.

12) van der Salm, H.; Fraser, M.G.; Horvath, R.; Cameron, S.A.; Barnsley, J.E.; Sun, X.Z.; George, M.W.; Gordon, K.C. Re(I) complexes of substituted dppz: A computational and spectroscopic study. *Inorg. Chem.* **2014**, *53*, 3126-3140.

13) Kondrasenko, I.; Kisel, K.S.; Karttunen, A.J.; Jänis, J.; Grachova, E.V.; Tunik, S.P.; Koshevoy, I.O. Rhenium(I) complexes with alkynylphosphane ligands: Structural, photophysical, and theoretical studies. *Eur. J. Inorg. Chem.* **2015**, 864-875.

14) Sarkar, R.; Rajak, K.K. Synthesis and characterization of rhenium(I) complexes based on *O*, *N*, *N* coordinating ligands: DFT/TDDFT studies on the electronic structures and spectral properties. *J. Organomet. Chem.* **2015**, *729*, 1-13.

15) Yang, X.Z.; Zhang, T.T.; Wei, J.; Jia, J.F.; Wu, H.S. DFT/TDDFT studies of the ancillary ligand effects on structures and photophysical properties of rhenium(I) tricarbonyl complexes with the imidazo[4,5-f]-1,10-phenanthroline ligand. *Int. J. Quantum Chem.* **2015**, *115*, 1467-1474.

16) Eng, J.; Daniel, D. Structural properties and UV−visible absorption spectroscopy of retinal-pyridyl-CN Re(I) carbonyl bipyridine complex: A theoretical study. *J. Phys. Chem. A* **2015**, *119*, 10645-10653.

17) Fumanal, M.; Daniel, C. Description of excited states in $[Re(imidazole)(CO)_3(phen)]^+$ including solvent and spin-orbit coupling effects: Density functional theory versus multiconfigurational wavefunction approach. *J. Comput. Chem.* **2016**, *37*, 2454-2466.

18) Hasheminasab, A.; Dawadi, M.B.; Mehr, H.S.; Herrick, R.S.; Ziegler, C.J. *Macromolecules*, **2016**, *49*, 3016-3027.

19) Yang, X.Z.; Wang, Y.L.; Guo, J.Y.; Zhang, T.T.; Jia, J.F.; Wu, H.S. The effect of group-substitution on structures and photophysical properties of rhenium(I) tricarbonyl complexes with pyridyltetrazole ligand: A DFT/TDDFT study. *Mater. Chem. & Phys.* **2016**, *178*, 173-181.

20) Kullapa Chanawanno, K.; Rhoda, H.M.; Hasheminasab, A.; Crandall, L.A.; King, A.J.; Herrick, R.S.; Nemykin, V.N.; Ziegler, C.J. Using hydrazine to link ferrocene with $Re(CO)_3$: A modular approach. *J. Organomet. Chem.* **2016**, *818*, 145-153.

21) Carreño, A.; Gacitúa, M.; Fuentes, J.A.; Páez-Hernández, D.; Peñaloza, J.P.; Otero, C.; Preite, M.; Molins, E.; Swords, W.B.; Meyer, G.J.; Manríquez, J.M.; Polanco, R.; Chávez, I.; Arratia-Pérez, R. Fluorescence probes for both prokaryotic and eukaryotic cells using new rhenium (I) tricarbonyl complexes with an electron withdrawing ancillary ligand. *New J. Chem.* **2016**, *40*, 7687-7700.

22) Harabuchi, Y.; Eng, J.; Gindensperger, E.; Taketsugu, T.; Maeda, S.; Daniel, C. Exploring the mechanism of ultrafast intersystem crossing in rhenium(I) carbonyl bipyridine halide complexes: Key vibrational modes and spin−vibronic quantum dynamics. *J. Chem. Theory Comput.* **2016**, *12*, 2335-2345.

23) Laramée-Milette, B.; Zaccheroni, N.; Palomba, F.; Hanan, G.S. Visible and near-IR emissions from k^2N- and k^3N-terpyridine rhenium(I) assemblies obtained by an [*n* x 1] head-to-tail bonding strategy. *Chem. Eur. J.* **2017**, *23*, 6370-6379.

24) Zúñiga, C.; Oyarzún, D. P.; Martin-Transaco, R.; Yáñez-S, M.; Tello, A.; Fuentealba, M.; Cantero-López, P.; Arratia-Pérez, R. Synthesis, characterization and relativistic DFT studies of *fac*-$Re(CO)_3$(isonicotinic acid)$_2$Cl complex. *Chem. Phys. Lett.* **2017**, *688*, 66-73.

25) Ramdass, A.; Sathish, V.; Velayudham, M.; Thanasekaran, P.; Umapathy, S.; Rajagopal, S. Luminescent sensor for copper(II) ion based on imine functionalized monometallic rhenium(I) complexes. *Sensors & Actuators B* **2017**, *240*, 1216-1225.

26) Chakraborty, I.; Jimenez, J.; Sameera, W.M.C.; Kato, M.; Mascharak, P.K. Luminescent Re(I) carbonyl complexes as trackable photoCORMs for CO delivery to cellular targets. *Inorg. Chem.* **2017**, *56*, 2863-2873.

27) Mukuta, T.; Peter V. Simpson, P.V.; Vaughan, J.G.; Brian W. Skelton, B.W.; Stagni, S.; Massi, M.; Koike, K.; Ishitani, O.; Onda, K. Photochemical processes in a rhenium(I) tricarbonyl *N*–heterocyclic carbene complex studied by time-resolved measurements. *Inorg. Chem.* **2017**, *56*, 3404-3413.
28) Muñoz-Osses, M.; Siegmund, D.; Gómez, A.; Godoy, F.; Fierro, A.; Llanos, L.; Aravena, D.; Metzler-Nolte, N. Influence of the substituent on the phosphine ligand in novel rhenium (I) aldehydes. Synthesis, computational studies and first insights into antiproliferative activity. *Dalton Trans.* **2018**, *47*, 13861-13869.
29) Shillito, G.E.; Hall, T.B.J.; Preston, D.; Traber, P.; Wu, L.; Reynolds, K.E.A.; Horvath, R.; Sun, X.Z.; Lucas, N.T.; Crowley, J.D.; George, M.W.; Kupfer, S.; Gordon, K.C. Dramatic alteration of ^{3}ILCT lifetimes using ancillary ligands in $[Re(L)(CO)_3(phen\text{-}TPA)]^{n+}$ complexes: An integrated spectroscopic and theoretical study. *J. Am. Chem. Soc.* **2018**, *140*, 4534-4542.
30) Whang, D.R.; Apaydin, D.H.; Park; S.Y.; Sariciftci, N.S. An electron-reservoir Re(I) complex for enhanced efficiency for reduction of CO_2 to CO. *J. Catal.* **2018**, *363*, 191-196.
31) Fumanal, M.; Gindensperger, E.; Daniel, C. Ligand substitution and conformational effects on the ultrafast luminescent decay of $[Re(CO)_3(phen)(L)]^+$ (L = imidazole, pyridine): non-adiabatic quantum dynamics. *Phys. Chem. Chem. Phys.* **2018**, *20*, 1134-1141.
32) Marker, S.C.; MacMillan, S.N.; Zipfel, W.R.; Li, Z.; Ford, P.C.; Wilson, J.J. Photoactivated in vitro anticancer activity of rhenium(I) tricarbonyl complexes bearing water-soluble phosphines. *Inorg. Chem.* **2018**, *57*, 1311-1331.
33) Klemens, T.; Świtlicka, A.; Szlapa-Kula, A.; Łapok, Ł.; Obłoza, M.; Siwy, M.; Szalkowski, M.; Maćkowski, S.; Libera, M.; Schab-Balcerzak, E.; Machura, B. Tuning optical properties of Re(I) carbonyl complexes by modifying push-pull ligands structure. *Organometallics* **2019**, *38*, 4206-4223.
34) Favale Jr., J.M.; Danilov, E.O.; Yarnell, J.E.; Castellano, F.N. Photophysical processes in rhenium(I) diiminetricarbonyl arylisocyanides featuring three interacting triplet excited states. *Inorg. Chem.* **2019**, *58*, 8750-8762.
35) Suntrup, L.; Stein, F.; Klein, J.; Wilting, A.; Parlane, F.G.L.; Brown, C.M.; Fiedler, J.; Berlinguette, C.P.; Siewert, I.; Sarkar, B. Rhenium complexes of pyridyl-mesoionic carbenes: Photochemical properties and electrocatalytic CO_2 reduction. *Inorg. Chem.* **2020**, *59*, 4215-4227.
36) Shillito, G.E.; Preston, D.; Traber, P.; Steinmetzer, J.; McAdam, C.J.; Crowley, J.D.; Wagner, P.; Kupfer, S.; Keith C. Gordon, K.C. Excited-state switching frustrates the tuning of properties in triphenylamine-donor-ligand rhenium(I) and platinum(II) complexes. *Inorg. Chem.* **2020**, *59*, 6736-6746.
37) Auvray, T.; Del Secco, B.; Dubreuil, A.; Zaccheroni, N.; Hanan, G.S. In-depth study of the electronic properties of NIR-emissive κ^3N-terpyridine rhenium(I) dicarbonyl complexes. *Inorg. Chem.* **2021**, *60*, 70-79.
38) Fernández-Terán, R.J.; Sévery, L. Coordination environment prevents access to intraligand charge-transfer states through remote substitution in rhenium(I) terpyridinedicarbonyl complexes. *Inorg. Chem.* **2021**, *60*, 1325-1333.
39) Fernández-Terán, R.J.; Sévery, L. Living long and prosperous: Productive intraligand charge-transfer states from a rhenium(I) terpyridine photosensitizer with enhanced light absorption. *Inorg. Chem.* **2021**, *60*, 1334-1343.

40) Kastl, A.; Dieckmann, S.; Wähler, K.; Völker, T.; Kastl, L.; Merkel, A. L.; Vultur, A.; Shanna, B.; Harms, K.; Ocker, M.; Parak, W. J.; Herlyn, M.; Meggers, E. Rhenium Complexes with Visible-Light-Induced Anticancer Activity. *ChemMedChem* **2013**, *8*, 924-927.

41) Kukovec, B.M.; Kodrin, I.; Mihalić, Z.; Furić, K.; Popović, Z. *Cis-trans* isomerism in cobalt(II) complexes with 3-hydroxypicolinic acid. Structural, DFT and thermal studies. *Inorg. Chim. Acta* **2010**, *363*, 1887-1896.

42) Smrečki, N.; Jović, O.; Molčanov, K.; Kukovec, B.M.; Kekez, I.; Matković-Čalogović, D.; Popović, Z. Influence of the non-coordinating alkyl chain type in *N*-alkylated iminodiacetamides on the stability and structure of their complexes with nickel(II) and cobalt(II). *Polyhedron* **2017**, *130*, 115-126.

43) Smrečki, N.; Rončević, T.; Jović, O.; Kukovec, B.M.; Maravić, A.; Gajski, G.; Čikeš-Čulić, V. Copper(II) complexes with *N'*-methylsarcosinamide selective for human bladder cancer cells. *Inorg. Chim. Acta* **2019**, *488*, 312-320.

References related to Discussion 2:

1) Becke, A. D. Density-functional exchange-energy approximation with correct asymptotic behavior. *Phys. Rev. A: At., Mol., Opt. Phys.* **1988**, *38*, 3098-3100.

2) Lee, C.; Yang, W.; Parr, R.G. Development of the Colle-Salvetti correlation-energy formula into a functional of the electron density. *Phys. Rev. B: Condens. Matter Mater. Phys.* **1988**, *37*, 785-789.

3) Becke, A. D. Density-functional thermochemistry. III. The role of exact exchange. *J. Chem. Phys.* **1993**, *98*, 5648-5652.

4) Grimme, S.; Antony, J.; Ehrlich, S.; Krieg, H. A consistent and accurate ab initio parameterization of density functional dispersion correction (DFT-D) for the 94 elements H-Pu. *J. Chem. Phys.* **2010**, *132*, 154104.

5) Yanai, T.; Tew, D. P.; Handy, N. C. A new hybrid exchange-correlation functional using the Coulomb-attenuating method (CAM-B3LYP). *Chem. Phys. Lett.* **2004**, *393*, 51-57.

6) Zhao, Y.; Schultz, N. E.; Truhlar, D. G. Exchange-correlation functional with broad accuracy for metallic and nonmetallic compounds, kinetics, and noncovalent interactions. *J. Chem. Phys.* **2005**, *123*, 161103.

7) Zhao, Truhlar, D. G. The M06 suite of density functionals for main group thermochemistry, thermochemical kinetics, noncovalent interactions, excited states, and transition elements: Two new functionals and systematic testing of four M06-class functionals and 12 other functionals. *Theor. Chem. Acc.* **2008**, *120*, 215-241.

8) MN15: Yu, H. S.; He, X.; Li, S. L.; Truhlar, D. G. MN15: A Kohn-Sham global-hybrid exchange-correlation density functional with broad accuracy for multi-reference and single-reference systems and noncovalent interactions. *Chem. Sci.* **2016**, *7*, 5032-5051.

9) Perdew, J. P.; Burke, K.; Ernzerhof, M. Generalized gradient approximation made simple. *Phys. Rev. Lett.* **1996**, *77*, 3865-3868.

10) Perdew, J. P.; Burke, K.; Ernzerhof, M. Errata: Generalized gradient approximation made simple. Phys. Rev. Lett. **1997**, *78*, 1396-1399.

11) PBE0: Adamo, C.; Barone, V. Toward reliable density functional methods without adjustable parameters: The PBE0 model. *J. Chem. Phys.* **1999**, *110*, 6158-6170.

12) Tao, J.; Perdew, J. P.; Staroverov, V. N.; Scuseria, G. E. Climbing the density dunctional ladder: Nonempirical meta-generalized gradient approximation designed for molecules and solids. *Phys. Rev. Lett.* **2003**, *91*, 146401-146405.

13) Staroverov, V. N.; Scuseria, G. E.; Tao, J.; Perdew, J. P. Comparative assessment of a new nonempirical density functional: Molecules and hydrogen-bonded complexes. *J. Chem. Phys.* **2003**, *119*, 12129-12137.

14) Chai, J.-D.; Head-Gordon, M. Systematic optimization of long-range corrected hybrid density functionals. *J. Chem. Phys.* **2008**, *128*, 084106.

15) wB97xD: Chai, J. D.; Head-Gordon, M. Long-range corrected hybrid density functionals with damped atom-atom dispersion corrections. *Phys. Chem. Chem. Phys.* **2008**, *10*, 6615-6620.

16) Mennucci, B.; Tomasi, J. Continuum solvation models: A new approach to the problem of solute's charge distribution and cavity boundaries. *J. Chem. Phys.* **1997**, *106*, 5151-5158.

17) Barone, V.; Cossi, M.; Tomasi, J. A new definition of cavities for the computation of solvation free energies by the polarizable continuum model. *J. Chem. Phys.* **1997**, *107*, 3210-3221.

18) Cancès, M. T.; Mennucci, B.; Tomasi, J. A new integral equation formalism for the polarizable continuum model: Theoretical background and applications to isotropic and anisotropic dielectrics. *J. Chem. Phys.* **1997**, *107*, 3032-3041.

19) Barone, V.; Cossi, M.; Tomasi, J. Geometry optimization of molecular structures in solution by the polarizable continuum model. *J. Comput. Chem.* **1998**, *19*, 404-417.

20) Tomasi, J.; Mennucci, B.; Cancès, M. T. The IEF version of the PCM solvation method: an overview of a new method addressed to study molecular solutes at the QM ab initio level. *J. Mol. Struct.: THEOCHEM* **1999**, *464*, 211-226.

21) Scalmani, G.; Frisch, M. J. Continuous surface charge polarizable continuum models of solvation.
I. General formalism. *J. Chem. Phys.* **2010**, *132*, 114110.

22) Lipparini, F.; Scalmani, G.; Mennucci, B.; Cances, E.; Caricato, M.; Frisch, M. J. A variational formulation of the polarizable continuum model. J. Chem. Phys. **2010**, *133*, 014106.

23) Rappé, A. K.; Casewit, C. J.; Colwell, K. S.; Goddard, W. A., III; Skiff, W. M. UFF, a full periodic table force field for molecular mechanics and molecular dynamics simulations. *J. Am. Chem. Soc.* **1992**, *114*, 10024-10039.

24) Pierotti, R. A. A scaled particle theory of aqueous and nonaqueous solutions. *Chem. Rev.* **1976**, *76* 717-726.

25) Floris, F.; Tomasi, J. Evaluation of the dispersion contribution to the solvation energy. A simple computational model in the continuum approximation. *J. Comp. Chem.* **1989**, *10*, 616-627.

26) Floris, F.; Tomasi, J.; Pascual-Ahuir, J. L. Dispersion and repulsion contributions to the solvation energy: Refinements to a simple computational model in the continuum approximation. *J. Comp. Chem.* **1991**, *12*, 784-791.

27) Kastl, A.; Dieckmann, S.; Wähler, K.; Völker, T.; Kastl, L.; Merkel, A. L.; Vultur, A.; Shanna, B.; Harms, K.; Ocker, M.; Parak, W. J.; Herlyn, M.; Meggers, E. Rhenium complexes with visible-light-induced anticancer activity. *ChemMedChem* **2013**, *8*, 924-927.
